# Availability and use of rapid diagnostic tests for the management of acute childhood infections in Europe: A cross-sectional survey of paediatricians

**DOI:** 10.1371/journal.pone.0275336

**Published:** 2022-12-20

**Authors:** Juan Emmanuel Dewez, Lucy Pembrey, Ruud G. Nijman, Stefano del Torso, Zachi Grossman, Adamos Hadjipanayis, Diego Van Esso, Emma Lim, Marieke Emonts, James Burns, Christèle Gras-LeGuen, Daniela Kohlfuerst, Hans Jürgen Dornbusch, Karen Brengel-Pesce, Francois Mallet, Ulrich von Both, Maria Tsolia, Irini Eleftheriou, Dace Zavadska, Ronald de Groot, Michiel van der Flier, Henriëtte Moll, Nienke Hagedoorn, Dorine Borensztajn, Rianne Oostenbrink, Taco Kuijpers, Marko Pokorn, Katarina Vincek, Federico Martinón-Torres, Irene Rivero, Philipp Agyeman, Enitan D. Carrol, Stéphane Paulus, Aubrey Cunnington, Jethro Herberg, Michael Levin, Aida Mujkić, Karin Geitmann, Liviana Da Dalt, Arūnas Valiulis, Risto Lapatto, Garyfallia Syridou, Péter Altorjai, Paul Torpiano, Ketil Størdal, Károly Illy, Artur Mazur, Mateja Vintar Spreitzer, Joana Rios, Corinne Wyder, Ivanna Romankevych, Romain Basmaci, Salvador Ibanez-Mico, Shunmay Yeung

**Affiliations:** 1 Clinical Research Department, London School of Hygiene & Tropical Medicine, London, United Kingdom; 2 Personalised Risk Assessment in Febrile Illness to Optimise Real-Life Management Across the European Union (PERFORM); 3 Department of Medical Statistics, London School of Hygiene and Tropical Medicine, London, United Kingdom; 4 Department of Infectious Diseases, Section of Paediatric Infectious Diseases, Imperial College London, London, United Kingdom; 5 Research in European Paediatric Emergency Medicine (REPEM); 6 ChildCare WorldWide, Padova, Italy; 7 European Academy of Paediatrics Research in Ambulatory Settings network (EAPRASnet); 8 Adelson School of Medicine, Ariel University, Ariel, Israel; 9 Pediatric Clinic, Maccabi Healthcare Services, Tel Aviv, Israel; 10 Paediatric Department, Larnaca General Hospital, Larnaca, Cyprus; 11 Medical School, European University Cyprus, Nicosia, Cyprus; 12 Primary Care Paediatrician, Health Care Centre Pere Grau, Barcelona, Spain; 13 Great North Children’s Hospital, Paediatric Immunology, Infectious Diseases & Allergy, Newcastle upon Tyne Hospitals NHS Foundation Trust, Newcastle upon Tyne, United Kingdom; 14 Population Health Sciences Institute, Newcastle University, Newcastle upon Tyne, United Kingdom; 15 Translational and Clinical Research Institute, Newcastle University, Newcastle upon Tyne, United Kingdom; 16 Centre d’Investigation Clinique CIC1413, INSERM-Nantes University Hospital, Nantes, France; 17 Medical University of Graz, Graz, Austria; 18 BioMérieux, Lyon, France; 19 University Hospital, Ludwig Maximilians University (LMU), Munich, Germany; 20 P. and A. Kyriakou Children’s Hospital, Athens, Greece; 21 Children Clinical University Hospital, Riga, Latvia; 22 Radboud University Medical Center, Nijmegen, the Netherlands; 23 Wilhelmina Children’s Hospital, University Medical Center Utrecht, Utrecht, the Netherlands; 24 Department of General Paediatrics, Erasmus MC-Sophia Children’s Hospital, Rotterdam, the Netherlands; 25 Amsterdam University Medical Center, Amsterdam, the Netherlands; 26 Department of Infectious Diseases, University Medical Centre Ljubljana, Ljubljana, Slovenia; 27 Hospital Clínico Universitario de Santiago, Santiago de Compostela, Spain; 28 Bern University Hospital, University of Bern, Bern, Switzerland; 29 Alder Hey Children’s Hospital, Liverpool, United Kingdom; 30 Department of Paediatrics, Oxford Vaccine Group, University of Oxford, Oxford, United Kingdom; 31 University of Zagreb, School of Medicine, Andrija Štampar School of Public Health, Zagreb, Croatia; 32 Primary Care Paediatrician, BVKJ, Hagen, Germany; 33 Department of Woman’s and Child’s Health Padova University Hospital, Padua, Italy; 34 Vilnius University Medical Faculty, Institute of Clinical Medicine, Clinic of Children’s Diseases, Vilnius, Lithuania; 35 Department of Paediatrics, Helsinki University Hospital, Helsinki, Finland; 36 Attiko University Hospital, Chaidari, Greece; 37 Association of Hungarian Primary Care Paediatricians, Budapest, Hungary; 38 Department of Paediatrics and Adolescent Health at Mater Dei Hospital, Valletta, Malta; 39 Paediatric Research Institute, University of Oslo, Oslo, Norway; 40 Dutch Society of Paediatrics NVK, Utrecht, the Netherlands; 41 Medical College of Rzeszow University, Rzeszów, Poland; 42 Zdravstveni dom Domžale, Slovenian Paediatric Society, Burnaby, Slovenia; 43 Hospital Beatriz Ângelo, Loures, Portugal; 44 Kinderärzte KurWerk, Burgdorf, Switzerland; 45 Ukrainian Academy of Pediatric Specialties, Ukraine; 46 Service de Pédiatrie-Urgences, AP-HP, Hôpital Louis-Mourier, Colombes, France; 47 Hospital Universitario Virgen de la Arrixaca, Murcia, Spain; 48 Department of Paediatrics, St Mary’s Imperial College Hospital, London, United Kingdom; McGill University Health Centre, CANADA

## Abstract

**Background:**

Point-of-care-tests (POCTs) have been advocated to optimise care in patients with infections but their actual use varies. This study aimed to estimate the variability in the adoption of current POCTs by paediatricians across Europe, and to explore the determinants of variability.

**Methods and findings:**

A cross-sectional survey was conducted of hospital and primary care paediatricians, recruited through professional networks. Questions focused on the availability and use of currently available POCTs. Data were analysed descriptively and using Median Odds Ratio (MOR) to measure variation between countries. Multilevel regression modelling using changes in the area under the receiver operating characteristic curve of models were used to assess the contribution of individual or workplace versus country level factors, to the observed variation.

The commonest POCT was urine dipsticks (UD) which were available to >80% of primary care and hospital paediatricians in 68% (13/19) and 79% (23/29) countries, respectively. Availability of all POCTs varied between countries. In primary care, the country (MOR) varied from 1.61 (95%CI: 1.04–2.58) for lactate to 7.28 (95%CI: 3.04–24.35) for UD. In hospitals, the country MOR varied from 1.37 (95%CI:1.04–1.80) for lactate to 11.93 (95%CI:3.35–72.23) for UD. Most paediatricians in primary care (69%, 795/1154) and hospital (81%, 962/1188) would use a diagnostic test in the case scenario of an infant with undifferentiated fever. Multilevel regression modelling showed that the country of work was more important in predicting both the availability and use of POCTs than individual or workplace characteristics.

**Conclusion:**

There is substantial variability in the adoption of POCTs for the management of acute infections in children across Europe. To inform future implementation of both existing and innovative tests, further research is needed to understand what drives the variation between countries, the needs of frontline clinicians, and the role of diagnostic tests in the management of acute childhood infections.

## Introduction

Fever is one of the commonest reasons for children to be presented to healthcare services [1]. Although most febrile children have self-limiting infections [2,3], the consequences of severe infections can be catastrophic. The clinical features of infections in children are often non-specific making it difficult to identify which children require antibiotics and which children may deteriorate and therefore require referral or admission [2]. Diagnostic uncertainty and avoidance of risk contributes to unnecessary admissions, and over-prescription of antibiotics [4], which may contribute to antimicrobial resistance [5].

Point-of-care tests (POCT) have the potential to improve patient care and antibiotic use depending on their accuracy, how quickly the results are available, how much they are trusted by the healthcare worker, and other factors. They have been widely advocated to reduce antibiotic resistance [5].

Existing POCTs which can be performed by clinicians at the point of care are available in a number of formats including dipsticks, lateral flow tests, and table-top or handheld devices into which samples are introduced directly (e.g. blood gas analysers) or using a cartridge or test strip. Some tests can detect the presence of a pathogen, e.g. the Group A Streptococcus (GAS) rapid test; others measure the host reaction to infection, such as C-reactive protein (CRP) POCTs or White Blood Cell count; or do both, for example urine dipsticks (UD), which detect nitrites produced by bacteria, and the host’s leucocyte-esterase.

Studies of their clinical effectiveness in the management acute childhood infections have mainly focused on their impact in reducing the use of antibiotics. A recent systematic review concluded that the use of GAS POCTs in primary care can reduce antibiotic prescription [6]. Other systematic reviews have found that the use of Influenza POCTs reduced the use of chest radiographs in children with respiratory infections in ambulatory care [7] and in emergency departments [8], and that CRP POCTs may allow reducing the use of antibiotics in children presenting with acute infections in primary care [9,10]. Another review found that urine dipsticks were effective in identifying children with urinary infections [11], but it is unclear if their use leads to improved clinical outcomes. Evidence about the clinical effectiveness of using other POCTs in children with acute infections is lacking. In terms of cost-effectiveness, one study [12] showed that using urine dipsticks in primary care was not cost-effective in the United Kingdom, and, to the best of our knowledge, there are no cost-effectiveness studies assessing the use of other POCTs in children with acute infections in European settings.

There is evidence of wide variation in how children with acute infections are managed in selected emergency departments across Europe [13,14], but limited data from a broader range of settings or focusing specifically on the availability and use of POCTs in this population. The use of POCTs in the management of adults in primary care varies [15–21], but few studies aimed at understanding the reason behind this variation.

The landscape for rapid diagnostic tests is changing rapidly. In order to inform implementation of future and existing tests, this study aimed to estimate the variability in the availability and use of existing POCTs for the management of acute childhood infections across Europe by paediatricians working in primary care and in hospitals, and to explore the determinants of variability.

## Materials and methods

### Study design and setting

An online cross-sectional survey of paediatricians from across Europe was conducted between September and November 2019.

### Participants

The inclusion criteria were: clinically active paediatricians working in primary care or in a hospital in one of 29 countries in Europe in which the research team had collaborators (S1 Supplementary materials).

Paediatricians were approached through research networks including the Personalised Risk assessment in febrile illness to optimise Real-life Management across Europe (PERFORM) consortium, European Academy of Paediatrics Research in Ambulatory Settings network (EAPRASnet), European Society of Paediatric Infectious Diseases, Research in European Paediatric Emergency Medicine, and national associations of paediatrics.

Within each network, an email invitation with a web-link was sent to all members. Clicking on the link directed participants to a participant information sheet and consent form. After providing electronic consent, participants were directed to the start of the questionnaire. Three reminders were sent two weeks apart. Participation was monitored weekly, and in countries with low participation, national coordinators further disseminated the survey through professional networks. No incentives were offered.

### POCTs and outcome measures

For this study we focused on the availability and use of POCTs that can help frontline paediatricians to make clinical decisions within the timeframe of the consultation, i.e. usually performed (including sampling and processing of the sample) by the doctor or nurse close to the patient and with results available within around 15 minutes. The nine POCTs were selected by consensus of experts from 11 European countries based on their knowledge of current paediatric practice in their countries and the results of a first pilot study (see below). We did not restrict the inclusion of POCTs to those for which there is evidence of clinical and cost-effectiveness because we are interested in the current “real world” availability and use of POCTs.

The following nine POCTs were included:

- POCTs that detect the presence of a pathogen (antigen-based tests only):
  ○ Group A Streptococcus (GAS) rapid POCTs
  ○ Respiratory syncytial virus (RSV) antigen-based POCTs
  ○ Influenza antigen-based POCTs
- POCTs that measure the host reaction to infection:
  ○ Full blood count or white blood cell count POCTs
  ○ C-reactive protein (CRP) POCTs
  ○ Procalcitonin POCTs
  ○ Blood gas POCTs to measure acid base status +/- glucose +/- lactate
  ○ POCTs measuring lactate alone
- POCTs that detect the presence of pathogens and host reaction:
  ○ Urine dipsticks (for nitrites and leucocyte esterase)

More recent (PCR) based POCTs were not included because to our knowledge they were not being routinely performed by frontline clinicians in any setting; most have a turnaround of > 30 minutes and are performed in laboratories by technicians at fixed times of the day. Thus they were felt to be outside the scope of the study.

The main outcomes of interest were the availability and use of existing POCTs used in the management of acute childhood infections in European settings. “Availability” was defined as the proportion of paediatricians reporting that the POCT was available in their workplace, and “use” was defined as the proportion of respondents who reported that they would use a POCT, if available, in a clinical scenario of an infant with undifferentiated fever (described below).

### Questionnaires

The questionnaires were developed to estimate the outcomes and their association with different factors. To investigate the use of POCTs we used a clinical vignette, with the aim of replicating a common scenario in which there is diagnostic uncertainty. We therefore based the vignette on a 4-month-old infant not severely unwell, but febrile without a focus (S2 Supplementary materials). Two similar questionnaires were developed in English, one for primary care and another for hospital paediatricians. These drafts were shared with paediatricians from 11 countries and adapted according to their feedback. The questionnaires were then piloted with 58 paediatricians at the 2017 European Academy of Paediatrics conference. Further revisions were made to improve the clarity and relevance of questions. The questionnaires were then translated into French, German, Greek, Hungarian, Italian, Latvian, Polish, Spanish, Slovenian, and Ukrainian, and then back translated into English by another blinded translator. The on-line English and Slovenian versions were piloted in Norway and Slovenia in June-July 2019 with 115 paediatricians. After correcting a few typographical errors and formatting, the final electronic formats were uploaded on the websites of the participating networks.

### Sample size

A sample size of 1064 primary care paediatricians and 1787 hospital paediatricians was computed allowing for the estimation of the main outcomes with 90% confidence, a margin of error below 10%, and an expected proportion of the outcomes of 50% in each country. We also assessed and confirmed that these sample sizes would be sufficient to identify determinants of the main outcomes in multivariable logistic regression analyses (S1 Supplementary materials).

### Analysis

Descriptive statistics were used to derive individual and workplace (primary care practice or hospital) characteristics and estimates of the availability and use of POCTs per country.

Multilevel logistic regressions were performed to assess the associations of variables at different levels of determinants (namely at the level of the workplace and at the level of the country) and the availability of POCTs. Assessing the separate effects of these two levels is important because workplace characteristics vary across workplaces, while country characteristics (e.g., laws, health regulations) are assumed to be the same across all workplaces within a country (S3 Supplementary materials for more explanations). For each POCT, we used a stepwise approach including three models based on the approach developed by Merlo and colleagues [22]: Model 1 was a simple logistic regression that included only workplace characteristics. This model was a base that was used as a comparator of Model 2. Model 2 was a multilevel logistic analysis which extended Model 1 by adding a second level, the country where the primary care practice or hospital was based. This multilevel analysis allows understanding the relative contributions of workplace characteristics and of country as a whole to the availability of POCTs. We compared the area under the receiver operating characteristic curve (AUC) of each model. Unlike clinical studies in which the change in AUC is often used to describe the increase in the accuracy of a diagnostic test, in this analysis the change in AUC between Models 1 and 2 is a measure of how the country as a whole contributes to predicting the availability of POCTs. If there is no change in AUC, the country as a whole does not contribute more than workplace characteristics; if there is an increase in AUC, this means that the country predicts better the availability of POCTs, and thus is a more important determinant. We also derived median odds ratios (MOR) [22] to assess the magnitude of the variation of availability between countries. The MOR is the median value of the distribution of ORs obtained when randomly picking two workplaces with the same characteristics from two countries and comparing the one with the higher availability of POCTs to the one with the lower availability. The MOR reflects the change of odds of availability of a POCT if a workplace would be in another country. If the MOR = 1, there is no change in odds. If the MOR>1, there is a variation across countries, and the larger the MOR the larger the variation is. Model 3 is an extension of Model 2 in which two country-specific characteristics (health expenditure per capita and financing scheme, see below) were added. While Model 1 and 2 assessed how much workplace characteristics and country as a whole explain the availability of POCTs, Model 3 sought to examine the extent to which the effect of country as a whole could be explained by the two measured country-specific characteristics. With regards the two country characteristics, we used 80% interval odds ratio (IOR80) [22]. IOR80 were used because in multilevel analyses the ORs of the higher level variables (here the two country characteristics) only allow comparison of workplaces within a country but not across countries. The IOR80 overcomes this limitation. When the IOR80 includes one, the variable effect is considered as minor (S3 Supplementary materials for more information). Model 3 was also used to assess the effect of workplace characteristics adjusting for county as a whole and for the two measured country characteristics, as these combined adjustments were not done in Models 1 and 2.

Directed acyclic graphs (DAGs) were developed to identify the variables that could be safely included in regression models to minimize confounding between the independent and dependent variables [23] (S4 Supplementary materials). The workplace variables in primary care were: sector of activity (private/public), practice size (solo/ group practice), turnaround time for routine tests such as C-reactive protein or full blood count from the external laboratory (continuous), distance to this laboratory (continuous), and who takes bloods (doctor/another person). In hospitals the variables were: sector of activity (private/public), level of care (secondary/tertiary hospital), hospital specialty (general hospital/paediatric or women’s and children’s hospital), turnaround time from the hospital laboratory for routine tests such as C-reactive protein or full blood count (continuous), and who takes blood (doctor/another person). The country-specific variables were: health expenditure per capita (continuous), and main financing scheme (government/mandatory health insurance/voluntary insurance or out-of-pocket).

A similar stepwise approach was used to identify determinants of POCT use in the clinical scenario, but only the two first steps were used as the two country-specific variables were not considered to potentially be associated with the clinicians’ decision to use POCTs. Model 4 included both workplace and clinicians’ characteristics, and Model 5 was again an extension of Model 4 in which the country as a whole was included as a second level (S3 Supplementary materials).

All participant questionnaires were included in the descriptive analyses. For the regression analyses, only questionnaires that provided data on the outcomes and potential explanatory variables were analysed (data flow chart in S5 Supplementary materials).

Continuous variables were not categorised to avoid arbitrary cut-offs. Quadratic transformations were used when the relationship between continuous variables and the outcome was not linear, and when the quadratic variable improved the fit of the models. All continuous variables were centred at the mean value of the observations. The models were estimated using Markov chain Monte Carlo methods (MCMC) to obtain robust parameter estimates with 95% Credible Intervals. The deviance information criterion (DIC) was used to assess and compare the goodness of fit of the models. A difference of more than ten in DIC is considered to show significant differences between models [24]. Analyses were performed with Stata 16® and MLwin 3.05®.

Ethical approval was obtained from the London School of Hygiene and Tropical Medicine Ethics Committee (Ref: 15977).

## Results

### Participant characteristics

**Fig 1:**
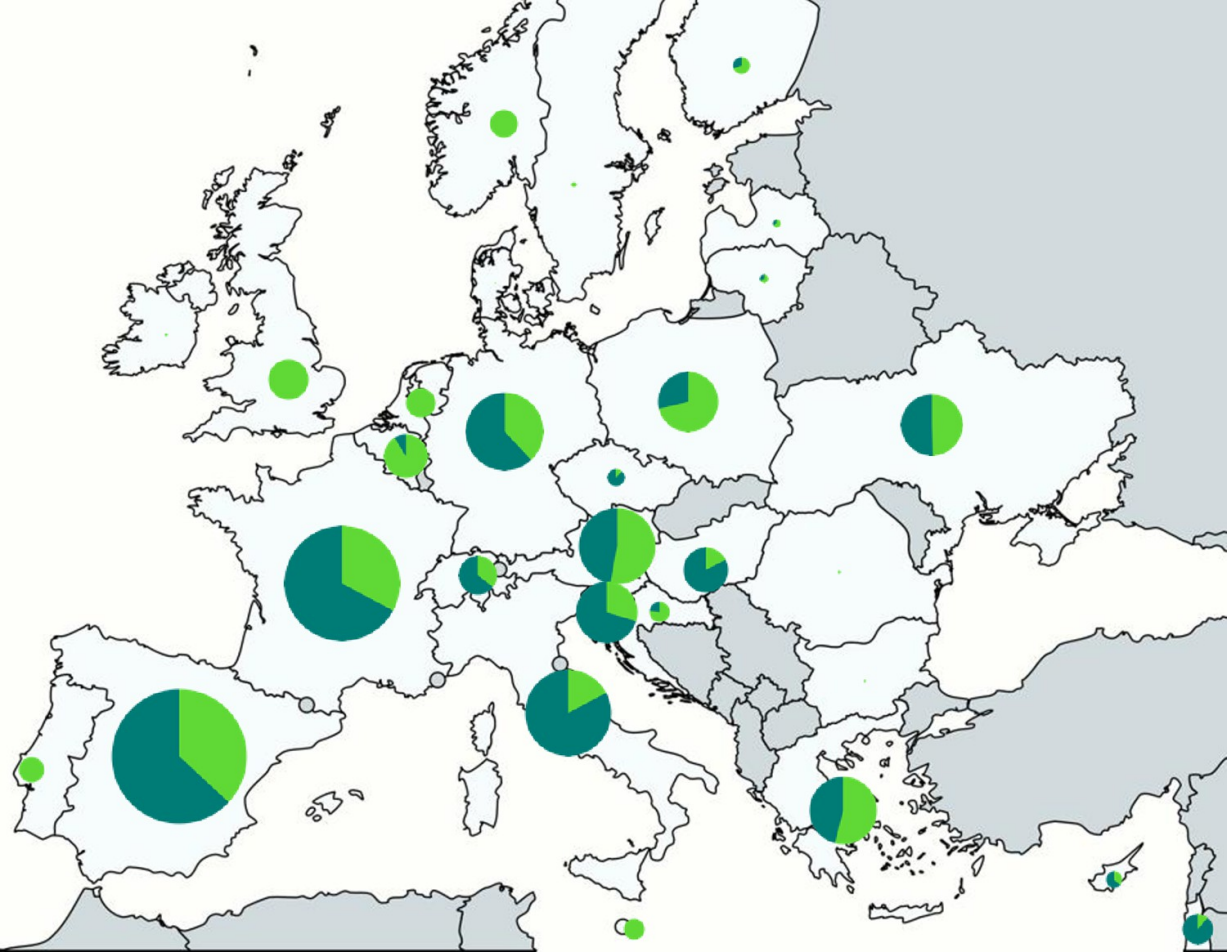
Origin of participants. Countries in light grey indicate the origin of participants. Total number of participants: Primary care participants n = 1,154; hospital participants n = 1,188. Number of participants is proportional to the pie size. The dark green slices indicate the share of primary care participants and the light green slices the share of hospital participant for a given country.

The study included 1154 primary care paediatricians from 19 countries, and 1188 hospital paediatricians from 504 unique hospitals from 29 countries (Fig 1 and data workflow in S5 Supplementary materials). The response rate per network cannot be estimated because paediatricians might have been members of several networks. Almost half (46.4%, 535/ 1154) of the primary care paediatricians had practiced for 30 or more years, and only 7.6% (88/1154) had practised for less than ten years. This compares with almost a quarter (22.4%, 266/1188) of the hospital paediatricians being trainees and 26.7% (317/1188) reporting less than ten years’ experience. Of the hospital paediatricians, a quarter were general paediatricians (24.8%, 295/1188), and 14.6% (173/1188) were infectious disease specialists. Around one third of primary care paediatricians worked in solo practices (34.7%, 400/1154) and in the private sector (37.1%, 428/1154). Most of the hospital paediatricians (91.9%, 463/1154) worked in public hospitals (Table 1).

**Table 1:** Characteristics of participants and workplaces.

**Characteristics of participants**					
	**Primary care paediatricians n = 1154** **n (%)**		**Hospital paediatricians n = 1188** **n (%)**		
**Expertise**	Paediatric trainee	22 (1.9)	Paediatric trainee	266 (22.4)	
	General paediatrician	1132 (98.1)	General paediatrician	295 (24.8)	
			Emergency medicine paediatrician	71 (6.0)	
			Infectious diseases paediatrician	173 (14.6)	
			Other paediatric subspecialty	383 (32.2)	
**Years of clinical**	<10	88 (7.6)	<10	317 (26.7)	
**practice**	10-<20	227(19.7)	10-<20	380 (32.0)	
	20-<30	304 (26.3)	20-<30	264 (22.2)	
	30-<40	418 (36.2)	30-<40	176 (14.8)	
	≥40	117 (10.1)	≥40	51 (4.3)	
**Typical duration of consultation in busy periods of the year (minutes)**	Median: 10 (IQR: 8–15)		Median: 15 (IQR: 15–20)		
**Characteristics of workplaces**					
	**Primary care practices n = 1154** **n (%)**		**Hospitals n = 504** **n (%)**		
**Sector of activity**	Private sector	428 (37.1)	**Sector of activity**	Private sector	41 (8.1)
	Public sector	726 (62.9)		Public sector	463 (91.9)
**Group or solo practice**	Solo	400 (34.7)	**Hospital level of care**	Secondary care	244 (48.4)
	Group	754 (65.3)		Secondary and tertiary care	260 (51.6)
**Distance to closest external**	Median: 1 (IQR: <1–5)		**Hospital specialty**	General hospital	334 (66.3)
**laboratory (km)**				Paediatric or women’s-and-children’s hospital	170 (33.7)
**Who takes bloods**	Doctors	176 (15.3)	**Who takes bloods**	Doctors	128 (25.4)
	Other healthcare worker	682 (59.1)		Other healthcare worker	376 (74.6)
	Bloods not taken	296 (25.7)			
**Shortest turnaround time for blood tests results from external lab (days)**	Median: 1 (IQR: <1–5)		**Shortest turnaround time for blood tests results from hospital lab (minutes)**	Median: 60 (IQR:45–90)	

### Availability of POCTs

**Fig 2:**
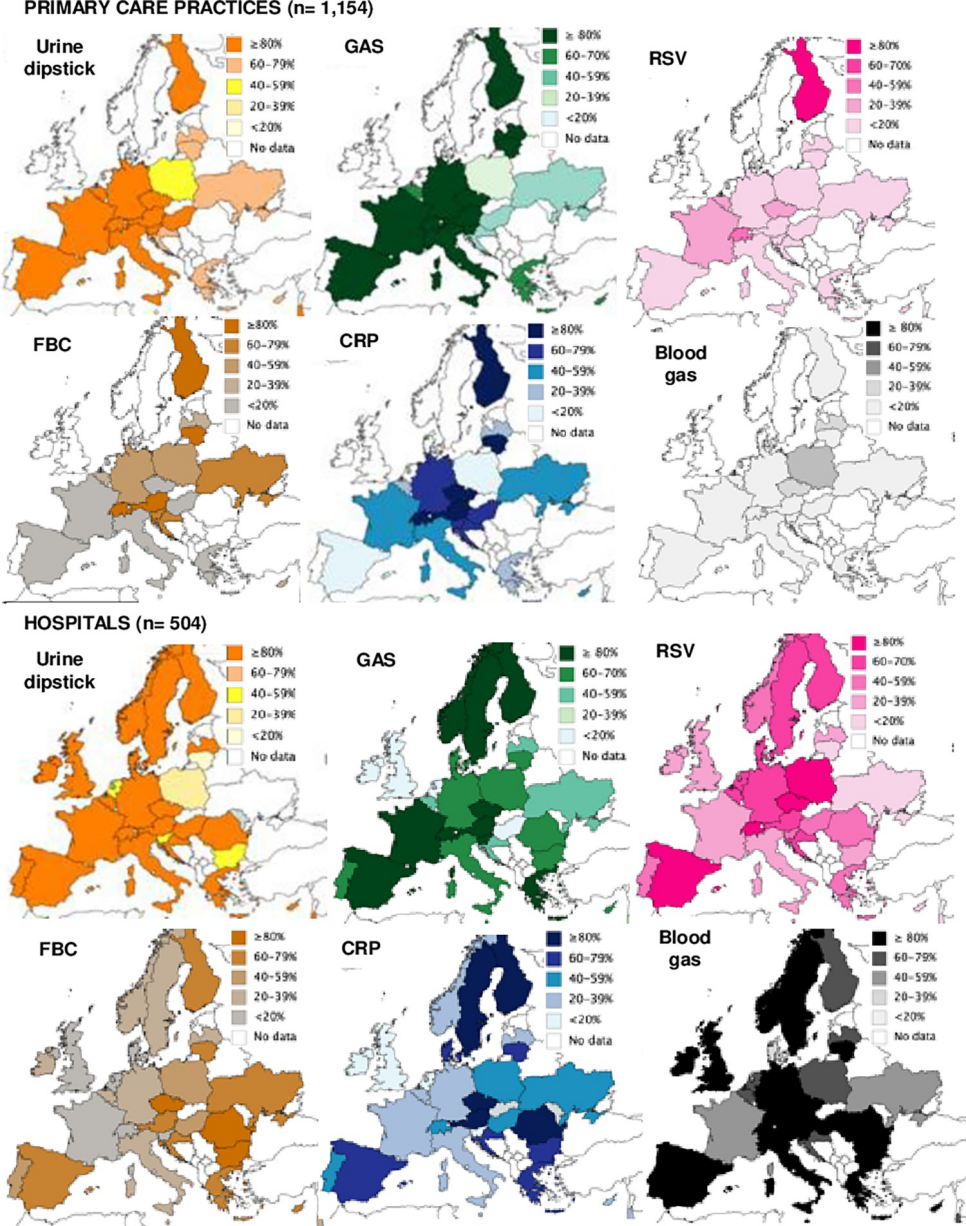
Percentage of paediatricians reporting availability of selected POCTs in each country. GAS: Group A Strep.; CRP: C-Reactive protein; FBC: Full blood count; RSV: Respiratory Syncytial Virus.

There was large variation in the reported availability of different POCTs across countries with the variation being greater for some POCTs than others (Fig 2). In primary care, urine dipsticks and GAS POCTs were the most available test and were available to over 80% of paediatricians in 68% (13/19) and 63% (12/19) of countries, respectively. Availability of other tests varied more, especially for CRP which was available to 40–79% of paediatricians in 42% (8/19) of countries, but to over 80% in 26% (5/19) of countries, and to under 20% of paediatricians in 10% (2/19) of countries. In hospitals, urine dipsticks were again the most available POCTs: in more than 80% of hospitals in 79% (23/29) of countries. Point-of-care blood gas analysis was also widely available with over 80% of paediatricians reporting it available in 65% (19/29) of countries. For other POCTs, availability varied greatly across different countries (S6 Supplementary materials for estimates per country).

### Importance of country of work on the availability of POCTs

**Fig 3:**
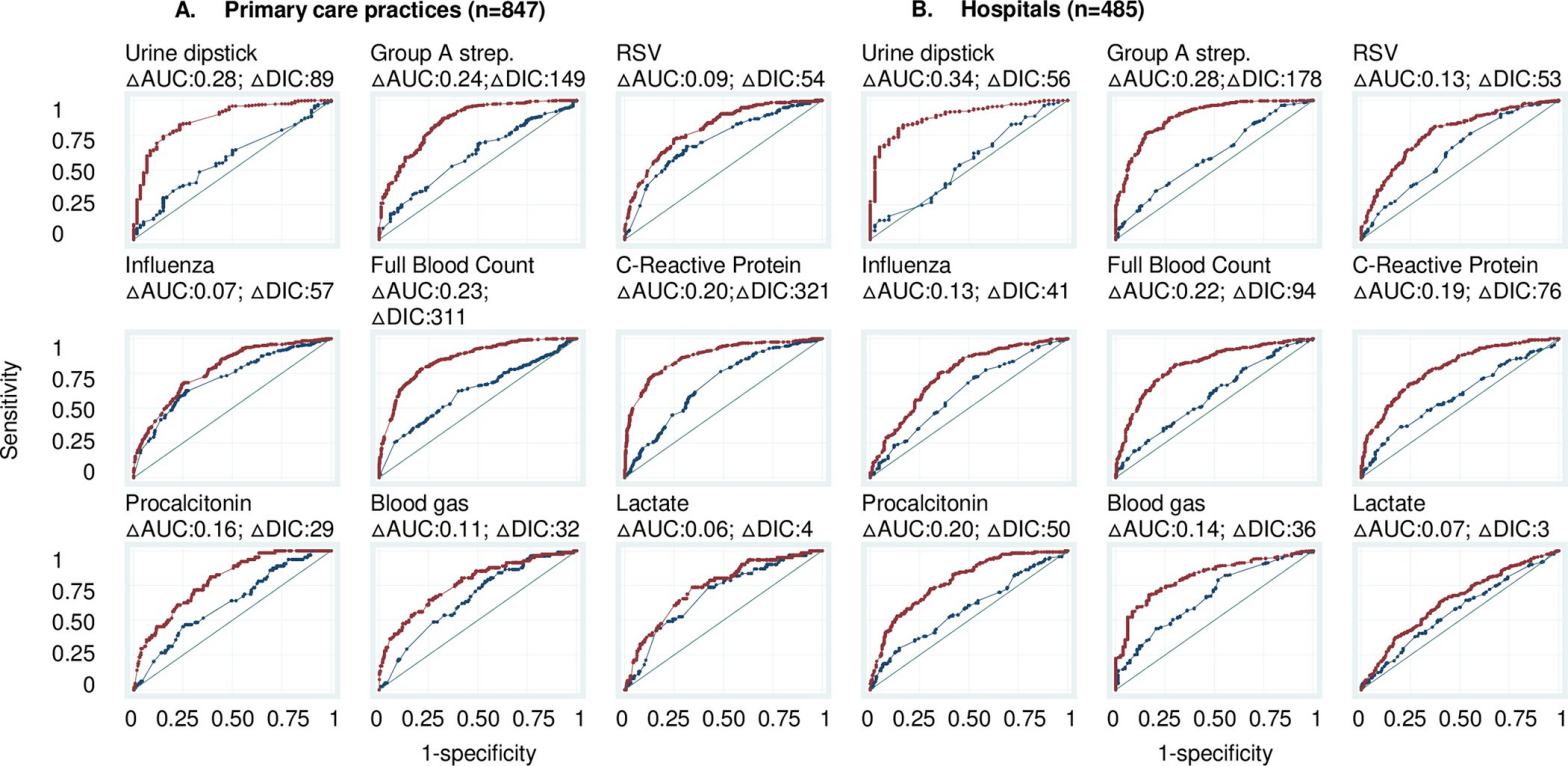
Area under the receiver operating curves (AUC) for the availability of POCTs in primary care practices and hospitals (Models 1 and 2). Blue curve: Model which adjusts for workplace characteristics (primary care practice or hospital characteristics (Model 1). Red curve: Model 1 + adjustment for country of work (Model 2). The greater AUC when including country of work indicates that country of work predicts better the availability of POCTs than workplace characteristics. The diagonal line represents an AUC of 0.50, i.e., a model with no predictive power. △AUC: Change in AUC when adding country of work (i.e., change in AUC when moving from Model 1 to Model 2). △DIC: Difference in Deviance Information Criterion. A △DIC>10 is considered a significant difference between models.

**Fig 4:**
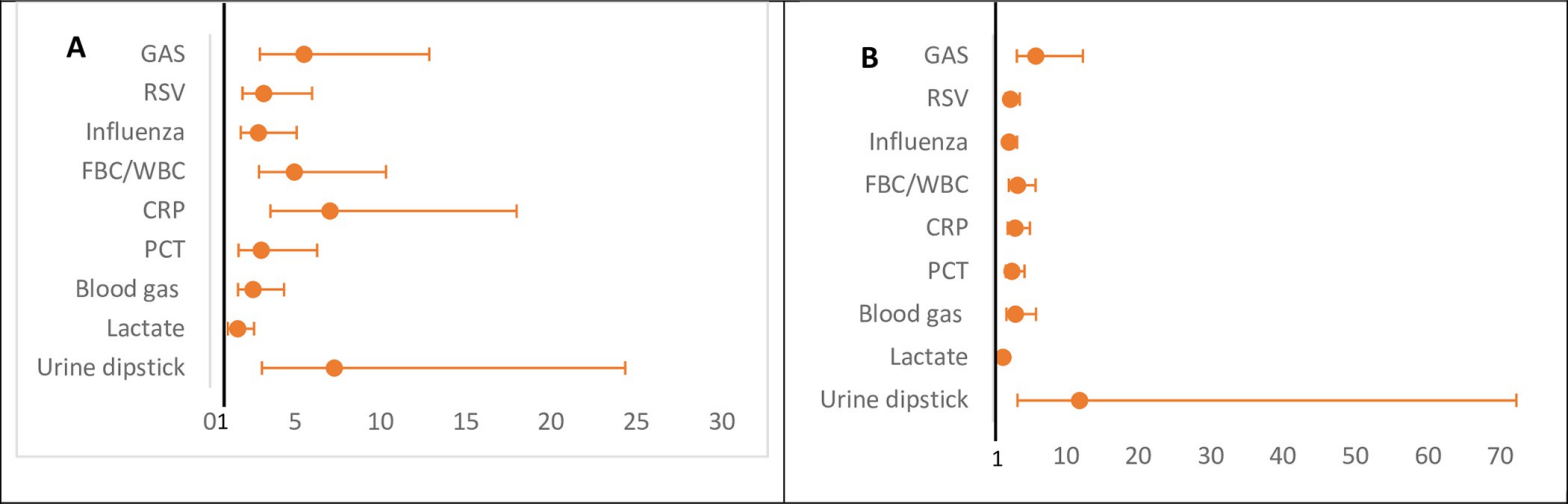
Variation in the availability of POCTs between the included countries expressed as median odds ratio (MORs). GAS: Group A streptococcus; RSV: Respiratory syncytial virus; FBC/WBC: Full blood count/White blood count; CRP: C-reactive protein; PCT: Procalcitonin. A: Country MORs for the availability of POCTs in primary care. B: Country MORs for the availability of POCTs in hospitals. MOR>1 indicate variation in the availability of POCTs across countries.

The country of work was more important in predicting the availability of all POCTs in primary care and hospitals (except for lactate POCT) than workplace characteristics, as shown by the comparison of the AUC of Models 1 and 2 (AUC increase: 0.06–0.34) (Fig 3). The country median odds ratios (MOR) was greater than one for all POCTs (Fig 4A and 4B). For primary care it varied from 1.61 (95%CI: 1.04–2.58) for lactate to 7.28 (95%CI: 3.04–24.35) for urine dipsticks. This indicates that the median change in odds of availability would increase by a factor of 1.61 for lactate POCT and a factor of 7.28 for urine dipsticks when moving from a country with lower odds to a country with higher odds chosen at random and shows that there is substantial between-country variation. For hospitals, the MOR varied from 1.37 (95%CI:1.04–1.80) for lactate to 11.93 (3.35–72.23) for urine dipsticks.

### Effect of specific country and workplace characteristics on the availability of POCTs

In Model 3 the two measured specific country characteristics (health expenditure per capita and main financing scheme) showed no significant effects either in primary care or in hospitals (Table 2).

**Table 2:** Effect of specific workplace and country level variables on the availability of POCTs in primary care practices and hospitals (Model 3).

**A. Primary care practices (observations: 847) **										
**Primary care practice variables**	**Statistic**	**Pathogen-based POCTs**			**Host-based POCTs**					**Pathogen and host-based tests**
		**GAS**	**RSV**	**Influenza**	**Full or white blood count**	**C-reactive protein**	**PCT**	**Blood gas**	**Lactate**	**Urine dipstick**
Private vs public primary care practice	OR (95% CrI)	2.29 (1.07–4.39)	2.36 (1.30–3.98)	2.76 (1.71–4.22)	0.80 (0.47–1.26)	1.64 (0.93–2.70)	2.32 (1.04–4.49)	0.99 (0.19–1.80)	1.75 (0.87–3.14)	1.91 (0.75–4.03)
Solo practice vs group practice	OR (95% CrI)	0.50 (0.24–0.91)	1.13 (0.66–1.80)	1.00 (0.64–1.49)	0.71 (0.42–1.11)	0.61 (0.36–0.95)	0.66 (0.25–1.37)	0.53 (0.24–1.00)	0.52 (0.21–1.04)	0.90 (0.37–1.86)
Distance to external laboratory (km)	OR (95% CrI)	1.00 (0.97–1.01)	0.95 (0.91–0.99)	0.97 (0.94–1.00)	0.97 (0.95–0.98)	0.99 (0.97–1.00)	0.96 (0.91–099)	0.93 (0.88–0.97)	0.93 (0.87–0.98)	0.97 (0.95–1.00)
Square distance to external laboratory*	OR (95% CrI)	NA	1.00 (1.00–1.00)	1.00 (1.00–1.00)	NA	NA	NA	NA	NA	NA
External laboratory turnaround time for routine tests**	OR (95% CrI)	0.76 (0.50–1.11)	1.47 (0.95–2.14)	1.11 (0.78–1.53)	1.01 (0.72–1.37)	1.20 (0.82–1.70)	1.32 (0.79–2.03)	0.88 (0.50–1.39)	1.81 (1.12–2.75)	1.47 (0.69–2.94)
Doctors take blood vs other health workers	OR (95% CrI)	NA	NA	NA	0.53 (0.30–0.85)	1.03 (0.57–1.73)	1.20 (0.43–2.60)	1.53 (0.70–2.89)	1.24 (0.49–2.50)	NA
**Country level variables**										
Health expenditure per capita	OR (95% CrI)	1.00 (1.00–1.00)	1.00 (1.00–1.00)	1.00 (1.00–1.00)	1.00 (1.00–1.00)	1.00 (1.00–1.00)	1.00 (1.00–1.00)	1.00 (1.00–1.00)	1.00 (1.00–1.00)	1.00 (0.99–1.00)
	IOR80	0.06–16.89	0.12–8.17	0.11–9.32	0.03–30.86	0.02–48.18	0.12–8.50	0.16–6.38	0.45–2.22	0.04–24.82
Government funding vs compulsory health insurance scheme	OR (95% CrI)	4.30 (0.33–18.07)	2.23 (0.35–7.94)	3.31 (0.22–18.76)	1.37 (0.95–6.36)	0.58 (0.02–2.94)	4.14 (0.66–16.42)	1.21 (0.26–3.97)	1.08 (0.41–2.65)	21.49 (0.67–129.46)
	IOR80	0.15–42.28	0.20–13.09	0.26–22.24	0.03–23.82	0.01–13.27	0.33–23.78	0.15–5.94	0.43–2.11	0.32–196.12
VHI-OOP vs compulsory health insurance scheme	OR (95% CrI))	4.15 (0.68–6.53)	7.50 (0.40–34.04)	4.04 (0.22–18.76)	4.07 (0.04–21.16)	1.87 (0.00–6.29)	4,18 (0.16–20.35)	2.55 (0.18–11.36)	1.25 (0.16–4.30)	4.74 (0.03–23.49)
	IOR80	0.06–17.93	0.44–29.40	0.22–19.36	0.03–28.35	0.00–8.52	0.21–14.89	0.23–9.17	0.40–1.98	0.04–26.62
	Country variance (95% CrI)	2.43 (0.68–6.53)	1.34 (0.33–3.70)	1.52 (0.46–3.81)	3.58 (1.42–8.26)	4.57 (1.71–11.01)	1.39 (0.19–4.48)	1.04 (0.23–2.89)	0.19 (0.00–0.97)	3.14 (0.49–10.79)
**B. Hospitals (observations: 485) **										
**Hospital variables**	**Statistic**	**GAS**	**RSV**	**Influenza**	**Full or white blood count**	**C-reactive protein**	**PCT**	**Blood gas**	**Lactate**	**Urine dipstick**
Private vs public primary hospital	OR (95% CrI)	3.38 (1.21–8.00)	1.76 (0.72–3.72)	1.91 (0.80–4.01)	2.57 (1.07–5.40)	3.05 (1.29–6.31)	2.83 (1.15–5.95)	2.88 (0.91–7.60)	1.82 (0.88–3.38)	4.41 (0.81–16.09)
Secondary vs tertiary level hospital	OR (95% CrI)	1.56 (0.84–2.66)	0.83 (0.51–1.28)	1.21 (0.75–1.86)	0.86 (0.51–1.38)	0.86 (0.52–1.32)	1.11 (0.63–1.84)	0.70 (0.35–1.23)	0.67 (0.43–1.00)	0.74 (0.29–1.56)
Paediatric/mother and child hospital vs general hospital	OR (95% CrI)	1.10 (0.55–1.88)	1.37 (0.79–2.20)	1.70 (1.01–2.73)	1.36 (0.77–2.24)	1.16 (0.67–1.87)	1.05 (0.57–1.78)	1.06 (0.52–1.95)	0.94 (0.58–1.43)	1.09 (0.38–2.48)
Hospital lab turnaround time for routine tests**	OR (95% CrI)	0.98 (0.96–0.99)	1.00 (1.00–1.00)	1.00 (0.99–1.00)	0.99 (0.98–1.00)	1.00 (1.00–1.00)	1.00 (1.00–1.00)	1.00 (1.00–1.00)	1.00 (1.00–1.00)	1.00 (1.00–1.01)
Square Hospital lab turnaround time for tests*	OR (95% CrI)	1.00 (1.00–1.01)	NA	NA	1.00 (1.00–1.01)	NA	NA	NA	NA	NA
Doctors take blood vs other health workers	OR (95% CrI)	NA	NA	NA	1.28 (0.57–2.53)	0.95 (0.40–1.96)	0.90 (0.34–1.89)	1.18 (0.34–2.96)	1.15 (0.65–1.84)	NA
**Country level variables**										
Health expenditure per capita	OR (95% CrI))	1.03 (0.97–3.89)	1.00 (1.00–1.00)	1.00 (1.00–1.00)	1.00 (1.00–1.00)	1.00 (1.00–1.00)	0.29 (0.02–4.39)	1.00 (1.00–1.00)	1.00 (1.00–1.00)	1.00 (1.00–1.00)
	IOR80	0.03–39.01	0.21–4.75	0.2–4.63	0.14–7.30	0.11–9.50	0.16–6.07	0.13–7.83	0.58–1.73	0.01–80.75
Government funding vs compulsory health insurance scheme	OR (95% CrI)	8.78 (0.10–56.66)	0.62 (0.23–1.36)	0.62 (0.23–1.32)	1.08 (0.29–2.72)	0.99 (0.26–2.57)	13.17 (0.68–254.08)	2.07 (0.50–5.71)	1.19 (0.69–1.89)	2.36 (0.11–65.63)
	IOR80	0.02–26.42	0.12–2.67	0.12–2.63	0.13–6.78	0.09–7.96	0.12–4.45	0.22–13.42	0.67–1.99	0.54–3498.27
VHI-OOP vs compulsory health insurance scheme	OR (95% CrI)	0.46 (0.03–6.99)	0.76 (0.10–2.69)	1.32 (0.19–4.68)	5.60 (0.40–25.72)	2.64 (0.19–11.83)	0.46 (0.03–6.99)	32.33 (0.85–182.54)	0.76 (0.23–1.91)	33.91 (4.23–1057.84)
	IOR80	0.06–96.36	0.11–2.55	0.20–4.38	0.42–22.46	0.16–14.35	0.07–2.73	1.28–78.46	0.38–1.14	0.03–202.12
	Country variance (95%CrI)	4.10 (1.73–8.61)	0.74 (0.26–1.67)	0.71 (0.23–1.67)	1.20 (0.42–2.79)	1.54 (0.58–3.40)	0.99 (0.31–2.42)	1.29 (0.31–3.44)	0.09 (0.00–0.36)	5.86 (1.03–20.61)

GAS: Group A Streptococcus; RSV: Respiratory syncytial virus; PCT: Procalcitonin; OR: Odds ratio; 95% CrI: 95% Credible Interval; IOR80: 80% Interval odds ratio; MOR: Median odds ratio; VHI-OOP: Voluntary health insurance-out of pocket; NA: Not applicable.

*Quadratic transformations were used when the relationship between continuous variables and the outcome was not linear, and when the quadratic variable improved the fit of the final models.

** such as C-reactive protein or full blood count.

In terms of specific workplace characteristics, few variables showed a significant effect. In primary care, compared to public facilities, private practices had higher odds of availability of several POCTs including GAS (OR: 2.29, 95%CI: 1.07–4.39), RSV (OR: 2.36, 95%CI: 1.30–3.98), influenza (OR: 2.76, 95%CI: 1.71–4.22), and procalcitonin (OR: 2.32, 95%CI: 1.04–4.49) POCTs. Solo practices had lower odds of availability of GAS (OR: 0.50, 95%CI: 0.24–0.91), and CRP (OR: 0.61, 95%CI: 0.36–0.95) POCTs compared to group practices. A longer distance to the external laboratory was associated with lower odds of availability of FBC (OR per each km increase: 0.97, 95%CI: 0.95–0.98), procalcitonin (OR: 0.96, 95%CI: 0.91–0.99), blood gas (OR: 0.93, 95%CI: 0.88–0.97), and lactate (OR: 0.93, 95%CI: 0.87–0.98) POCTs. In hospitals, private hospitals had higher odds of availability of GAS (OR: 3.38, 95%CI: 1.21–8.00), FBC (OR: 2.57, 95%CI: 1.07–5.40), CRP (OR: 3.05, 95%CI: 1.29–6.31), and procalcitonin (OR: 2.83, 95%CI: 1.15–5.95) POCTs.

### Use of POCTS in the clinical scenario

69% (795/1154) of primary care paediatricians and 81% (962/1188) of hospital paediatricians reported they would use at least one diagnostic in the clinical scenario. Urine dipsticks, CRP, and influenza were the most commonly cited POCTs by primary care paediatricians (90%, 65%, and 51%, respectively), and hospital paediatricians (61%, 48%, and 50%, respectively) (S7 Supplementary materials for estimates per country).

### Importance of country as a whole on the reported use of POCTs in the clinical scenario

**Fig 5:**
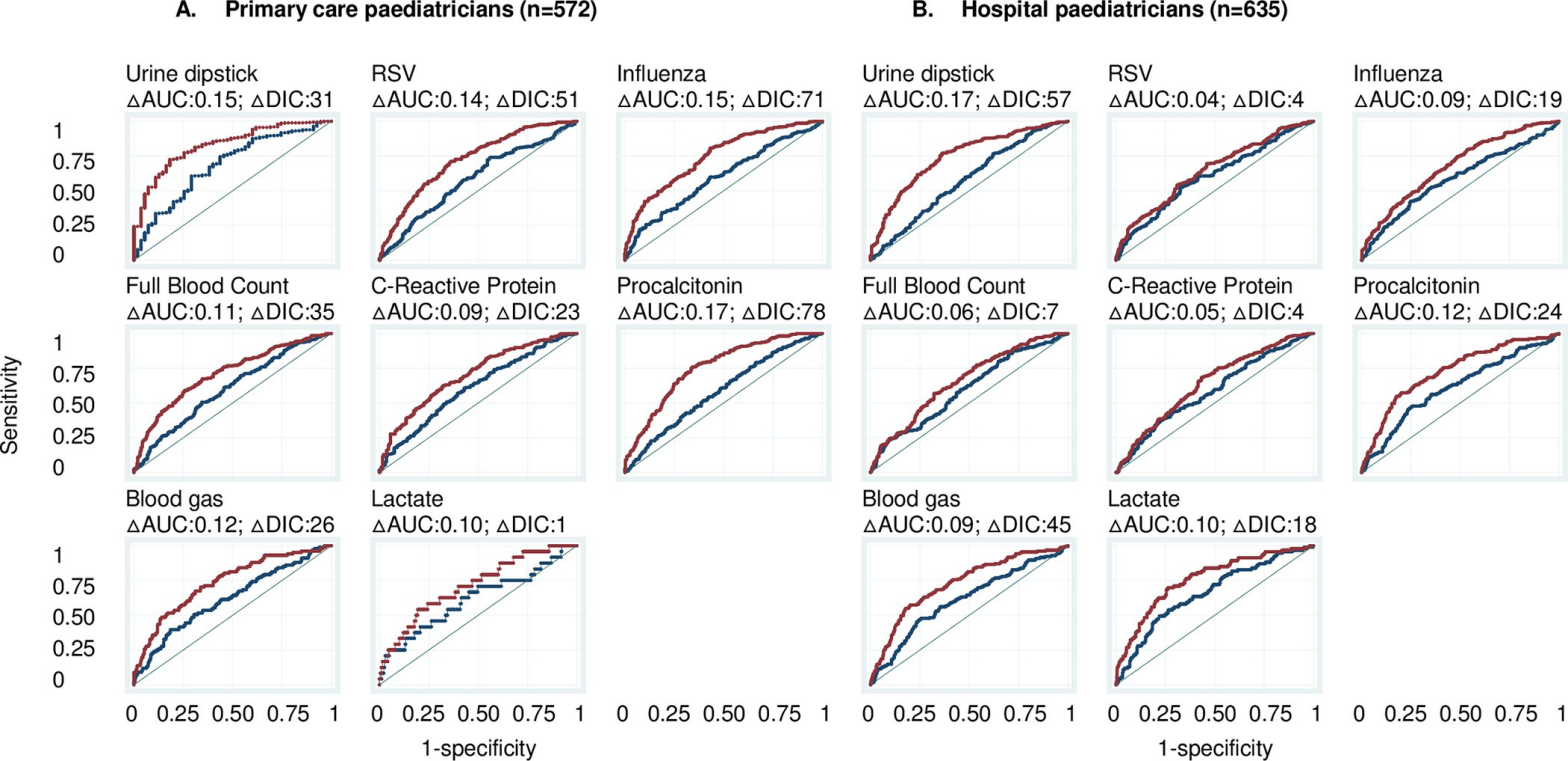
Area under the receiver operating characteristic curves (AUC) for the use of POCTs in the clinical scenario by primary care and hospital paediatricians (Models 4 and 5). Blue curve: Model which adjusts for paediatrician and workplace characteristics (Model 4). Red curve: Model 4 + adjustment for country of work (Model 5). The greater AUC when including country of work indicates that country of work predicts better the use of POCTs than workplace characteristics. The diagonal line represents an AUC of 0.50, i.e., a model with no predictive power. △AUC: Change in AUC when adding country of work (i.e., change in AUC when moving from Model 4 to Model 5). △DIC: Difference in Deviance Information Criterion. A △DIC>10 is considered a significant difference between models.

The comparison of Models 4 and 5 showed that adding the country improved the prediction of the use of all POCTs in the clinical scenario (AUC increase: 0.04–0.17), but this was not significant for lactate by primary care paediatricians, and for RSV, FBC, and CRP by hospital paediatricians (Fig 5). This shows again that the country as a whole is overall more important to predict the use of most POCTs than workplace and clinician characteristics, although the increases in AUC between Models 4 and 5 were smaller overall compared to the increases in AUC between models 1 and 2.

**Fig 6:**
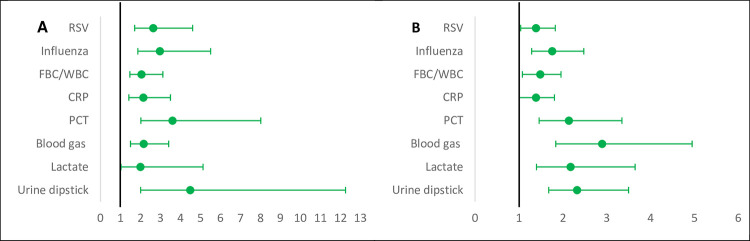
Variation in the use of POCTs between the included countries expressed as median odds ratio (MORs). GAS: Group A streptococcus; RSV: Respiratory syncytial virus; FBC/WBC: Full blood count/White blood count; CRP: C-reactive protein; PCT: Procalcitonin. A: Country MORs for the use of POCTs in primary care. B: Country MORs for the use of POCTs in hospitals. MOR>1 indicate variation in the use of POCTs across countries.

The country MOR was greater than one for all POCTs (Fig 6B and 6C). It varied from 2.00 (95%CI:1.03–5.13) for lactate to 4.49 (95%CI:2.01–12.26) for urine dipsticks use in primary care, and from 1.39 (95%CI:1.01–1.81) for CRP to 2.90 (95%CI:1.84–4.95) for blood gas use in hospitals.

### Effect of specific workplace and clinician characteristics on the use of POCTs in the clinical scenario

Few specific workplace or clinician characteristics showed a significant effect on the use of POCTs. In primary care, a longer turnaround time for diagnostics results by the external laboratory was associated with higher odds of using CRP POCT (OR: 6.20; 95%CI:1.55–18.01) and lactate POCTs (OR: 2.07; 95%CI:1.13–3.43) (Table 3A). In hospitals, clinicians working in paediatric or women and children hospitals had higher odds of using FBC (OR: 3.38, 95%CI: 1.21–8.00), and procalcitonin POCTs (OR: 3.38, 95%CI: 1.21–8.00) compared to those working in general hospitals. Working in a hospital where doctors take bloods was associated with higher odds of using bloods gas (OR: 2.44, 95%CI: 1.13–4.56), and lactate POCTs (OR: 2.47, 95%CI: 1.05–4.76) (Table 3B). No clinicians’ characteristic was significant in either primary care or hospitals.

**Table 3:** Effect of clinician, workplace variables, and country of work on the use of POCTs in the clinical scenario (Model 5).

**A. Primary care (observations: 572)**									
**Clinician and primary care practice variables**	**Statistic**	**Pathogen-based POCTs**		**Host-based POCTs**					**Pathogen and host-based tests**
		**RSV**	**Influenza**	**Full or white blood count**	**C-reactive protein**	**PCT**	**Blood gas**	**Lactate**	**Urine dipstick**
Years of clinical practice	OR (95% CrI)	1.00 (0.98–1.02)	0.99 (0.97–1.01)	1.13 (1.04–1.22)	1.01 (0.99–1.03)	1.08 (1.00–1.17)	0.90 (0.82–0.99)	1.01 (0.98–1.06)	1.05 (1.02–1.08)
Square years of clinical practice*	OR (95% CrI)	NA	NA	1.00 (1.00–1.00)	NA	1.00 (1.00–1.00)	1.00 (1.00–1.00)	NA	NA
Average consultation time	OR (95% CrI)	1.00 (0.97–1.03)	1.01 (0.97–1.04)	0.98 (0.95–1.01)	0.98 (0.95–1.01)	1.00 (0.96–1.04)	1.00 (0.96–1.04)	0.97 (0.88–1.04)	0.98 (0.94–1.03)
Private practice vs public practice	OR (95% CrI)	0.90 (0.52–1.46)	0.86 (0.49–1.39)	1.36 (0.79–2.21)	1.78 (1.02–2.93)	1.22 (0.67–2.05)	0.71 (0.36–1.26)	1.30 (0.34–3.34)	2.72 (0.95–6.58)
Solo practice vs group practice	OR (95% CrI)	1.36 (0.83–2.13)	0.93 (0.56–1.44)	0.95 (0.58–1.49)	0.88 (0.51–1.41)	0.98 (0.57–1.57)	1.11 (0.58–1.91)	0.70 (0.16–1.85)	2.04 (0.74–4.79)
Distance to external laboratory	OR (95% CrI)	1.00 (0.99–1.02)	1.00 (0.98–1.02)	0.99 (0.97–1.00)	0.99 (0.97–1.01)	1.00 (0.98–1.02)	0.99 (0.97–1.01)	0.99 (0.95–1.03)	1.00 (0.97–1.03)
External laboratory turnaround time for routine tests**	OR (95% CrI)	1.23 (0.88–1.68)	1.34 (0.94–1.85)	1.30 (0.93–1.78)	6.20 (1.55–18.01)	0.85 (0.59–1.18)	1.46 (0.97–2.11)	2.07 (1.13–3.43)	0.92 (0.47–1.65)
Square external laboratory turnaround time*	OR (95% CrI)	NA	NA	NA	0.71 (0.51–0.93)	NA	NA	NA	NA
Doctors take blood vs other health workers	OR (95% CrI)	NA	NA	0.67 (0.38–1.10)	1.06 (0.58–1.78)	1.15 (0.64–1.92)	1.94 (0.99–3.42)	1.14 (0.23–3.22)	NA
**Country**									
	Country variance (95%CrI)	1.03 (0.32–2.57)	1.30 (0.42–3.20)	0.57 (0.16–1.42)	0.64 (0.13–1.72)	1.80 (0.54–4.76)	0.65 (0.18–1.65)	0.52 (0.00–2.94)	2.48 (0.54–6.90)
**B. Hospitals (observations: 635) **									
**Clinician and hospital variables**	**Statistic**	**RSV**	**Influenza**	**Full or white blood count**	**C-reactive protein**	**PCT**	**Blood gas**	**Lactate**	**Urine dipstick**
Years of clinical practice	OR (95% CrI)	1.01 (0.99–1.03)	1.01 (0.99–1.03)	1.00 (0.98–1.03)	1.02 (1.00–1.04)	1.02 (0.99–1.04)	0.98 (0.95–1.00)	1.00 (097–1.03)	1.00 (0.98–1.02)
Trainee doctor vs general paediatrician	OR (95% CrI)	1.80 (0.99–3.08)	1.03 (0.56–1.74)	1.21 (0.65–2.11)	1.52 (0.85–2.56)	1.23 (0.57–2.34)	0.35 (0.17–0.66)	0.73 (0.29–1.54)	1.31 (0.67–2.30)
Specialist vs general paediatrician	OR (95% CrI)	1.56 (0.99–2.39)	1.29 (0.81–1.95)	0.62 (0.38–0.95)	0.93 (0.60–1.40)	1.45 (0.83–2.39)	0.92 (0.53–1.49)	0.96 (0.49–1.72)	1.25 (0.76–1.94)
Average consultation time	OR (95% CrI)	1.00 (0.98–1.02)	1.00 (0.98–1.01)	0.99 (0.97–1.01)	1.01 (1.00–1.03)	1.01 (0.99–1.03)	1.02 (1.00–1.05)	1.03 (1.00–1.05)	0.99 (0.97–1.01)
Private vs public primary hospital	OR (95% CrI)	1.52 (0.66–3.06)	1.34 (0.58–2.69)	1.57 (0.64–3.20)	1.23 (0.54–2.39)	2.09 (0.82–4.37)	0.82 (0.26–1.89)	1.47 (0.38–3.58)	1.17 (0.47–2.48)
Secondary vs tertiary level hospital	OR (95% CrI)	0.91 (0.60–1.32)	1.14 (0.75–1.66)	1.03 (0.65–1.55)	1.12 (0.74–1.61)	1.15 (0.69–1.81)	0.85 (0.51–1.32)	1.22 (0.66–2.07)	0.66 (0.41–1.01)
Paediatric/women’s and children’s hospital vs general hospital	OR (95% CrI)	1.31 (0.87–1.92)	1.49 (0.97–2.19)	1.67 (1.05–2.56)	1.25 (0.82–1.81)	1.74 (1.04–2.77)	1.09 (0.65–1.74)	1.29 (0.68–2.22)	0.96 (0.58–1.49)
Hospital lab turnaround time for routine tests**	OR (95% CrI)	1.01 (1.00–1.02)	1.00 (1.00–1.00)	1.00 (1.00–1.00)	1.00 (1.00–1.00)	1.00 (1.00–1.00)	1.00 (0.99–1.00)	1.00 (1.00–1.00)	1.00 (1.00–1.00)
Square hospital lab turnaround time for tests*	OR (95% CrI)	1.00 (1.00–1.00)	NA	NA	NA	NA	NA	NA	NA
Doctors take blood vs other health workers	OR (95% CrI)	NA	NA	1.30 (0.75–2.04)	1.20 (0.73–1.81)	1.47 (0.73–2.71)	2.44 (1.13–4.56)	2.47 (1.05–4.76)	NA
**Country**									
	Country Variance (95%CrI)	0.12 (00–0.41)	0.35 (0.07–0.91)	0.17 (0.01–0.49)	0.12 (0.00–0.38)	0.64 (0.16–1.60)	1.25 (0.41–2.81)	0.67 (0.12–1.84)	0.79 (0.29–1.72)

RSV: Respiratory syncytial virus; PCT: Procalcitonin; OR: Odds ratio; 95%; CrI: 95% Credible Interval; MOR: Median odds ratio; NA: Not applicable.

*Quadratic transformations were used when the relationship between continuous variables and the outcome was not linear, and when the quadratic variable improved the fit of the final models.

** such as C-reactive protein or full blood count.

## Discussion

### Summary of findings

The availability and use of POCTs in the management of children with suspected infections varies substantially across Europe. By far the strongest determinant of whether or not POCTs are available to and used by paediatricians is the country in which they work. The reasons for this are likely to be complex and could not be simply explained by differences in how health care is financed within countries, nor overall healthcare expenditure per capita. The latter finding contrasts with the other reports which have found that the availability of in vitro diagnostics overall, and of expensive diagnostics such as CT-scans or MRIs, are associated with health expenditure per capita [25].

We found that operating as a private facility is associated with an increased odds of availability of some POCTs but was not associated with use in the clinical scenario. POCTs can be a source of income in these settings [26,27], however in our study, the clinical scenario was a febrile infant which is unlikely to be the type of patient on which diagnostics would be overused to increased income.

The pattern of availability and use varied by type of POCT, but overall urine dipsticks and GAS were the most commonly available POCTs in primary care, while in hospitals it was urine dipsticks and blood gas POCTs. Previous studies have been limited in either the range of POCTs examined or the number of countries included. For urine dipsticks, previous studies have also reported their wide availability: a survey of paediatricians from eight countries found that 74% of paediatricians use UD [28], and a survey of GPs from Belgium, Holland, and the UK showed that they were available in 87%, 96%, and 90% of practices, respectively [18]. Reports on the availability of GAS POCT varied more. The above survey of GPs found that GAS POCTs were only available in 4%, 1%, and 15% of Belgian, Dutch, and British practices, respectively. By contrast, surveys of French GPs [29], and of Spanish primary care paediatricians found that GAS POCT were available to 88% and 79% of participants, respectively [30], which is in line with our findings for those countries.

In a clinical scenario of an infant with undifferentiated fever most paediatricians reported that they would use some kind of diagnostic tests with urine dipsticks, CRP, and influenza being the most commonly cited POCTs in both settings. This is in line with a study of children attending EDs in eight countries which found that 78% of infants with undifferentiated fever were tested [14].

### Strength and limitations

Main strengths of this study are its size and breadth, that it addresses an important but under-researched question, and employed a robust approach to both the study design and analysis.

We included a large number of community- and hospital-based paediatricians from many countries across Europe and sought to be as complete in our sampling as possible. However, we did not manage to achieve our pre-defined sample size, and given the sampling approach there is a possibility of selection bias. Due to resource constraints we limited the scope of this study to paediatricians and did not extend it to General Practitioners, who in some countries are the main providers of medical care for children in the community.

The survey was developed through a robust process including the expertise of paediatricians from 11 countries, two pilot studies, the translation of the questionnaires into ten languages, and the use of software with quality-assurance checks. In order to maximise participation and completion rates we kept the questionnaire as short as possible. We therefore limited the clinical scenario to just one case, limiting generalisability of the findings about how paediatricians would use POCTs. We also omitted important topics or questions, for example the influence of evidence (or the lack of) on participants decision in the clinical scenario, or the perceptions about quality assurance. We used turnaround time for routine tests such as C-reactive protein or full blood count as a proxy for the time needed to obtain results from the closest laboratory in general, but turnaround times can vary substantially across different tests. We used the Bayesian MCMC methods to compute robust parameter estimates and direct acyclic graphs (DAGs) in order to identify the variables that could be safely included in the regression models while minimising confounding. However, the risk of confounding cannot be eliminated as DAG cannot entirely accurately represent of reality.

### Implication for the implementation of POCTs and future research

As mentioned earlier we found substantial variability in the availability and use of current POCTs between European countries that we were not able to explain but is likely to be due to complex interplay of different factors in each country. This might include differences in patient care pathways, the capacity and readiness to innovate, the processes to identify new technologies, the availability of resources including funding, the existence of alternative diagnostic tools, as well as diversity in policies and regulations, including quality assurance standards for the use of diagnostic tests. Moreover, the implementation of innovations, including POCTs, can be disruptive for healthcare services because they may impact on the roles of healthcare workers (because they may need to change their practice), on the routine organisations of health services, on the allocation of funding (funding for the innovation could be at the expense of funding other interventions), and the work needed to normalise the innovation can be substantial (e.g. training all staff that are already very busy) [31]. From a rational national health systems perspective, the choice of which medical interventions should be implemented, should ideally be based on an assessment of their clinical-effectiveness, cost-effectiveness, and the broader impacts on health services–part of the remit of Health Technology Assessment organisations. Most of the available evidence on current POCTs, however, focuses on diagnostic test accuracy [32] and as mentioned in the introduction, current evidence about the use of POCTs in children is available only for few POCTs. The lack of such evidence means that the decision to implement diagnostic tests is more likely to be influenced by other factors which are specific to each country and local HTA processes [33]. There are around 50 HTA agencies across Europe [34], and they may use different processes and criteria when assessing new diagnostic test. Additional in-depth studies are needed to understand how specific country factors influence the availability of POCTs in specific countries.

Once POCTs are made available, whether or not clinicians use them could vary depending on factors including clinicians’ perceptions of the added value of the tests in, for example, reducing diagnostic uncertainty [35], or their user friendliness. The practices and perception of paediatricians about the use of current POCTs in children with acute infections need also to be explored and compared across Europe through qualitative methods.

The study focussed on the POCTs that were being performed routinely by frontline line clinicians in some study countries at the time of the survey. Since then, change in diagnostic landscape has accelerated dramatically in part due to the impact of the COVID-19 pandemic. Use of POCTs has become much more widespread, as has the availability of multiplex tests that are able to identify the presence of multiple pathogens [36]. Innovative “-omics” based tests which focus on differentiating bacterial from viral infections [37,38], and multiclass tests [39] are under development. How these can impact on reducing diagnostic uncertainty and improving patient management is not clear, and further studies aimed at exploring how existing and new tests should be optimally deployed would be extremely useful. One drawback of pathogen-based tests is that the presence of microorganisms does not necessarily mean they are the cause of disease. Tests that measure the host’s response to specific pathogens [40] or combine the detection of microorganism with the measurement of host biomarkers may be most useful in this regard. In terms of the management of children with acute infections, we believe that the main diagnostic gaps that need to be addressed is the prediction of severity. This would be particularly useful in children with substantial diagnostic uncertainty, such as young infants with undifferentiated fever, children with low respiratory infections in the “grey zone” (i.e., not severely ill, but raising some concern), or vulnerable children (e.g., immunocompromised children) presenting an episode of acute fever. European paediatricians and other healthcare workers who see children in consultation may have additional needs in terms of diagnostic tools. Additional studies aiming at identifying these needs would be important to inform the development of new tests. Ideally, once new tests are developed, studies aiming to assess all the different steps in the evaluation of diagnostic tests suggested by Horvath and colleagues [41] should be undertaken. This includes, as mentioned above, diagnostic test accuracy studies, clinical effectiveness studies, cost-effectiveness studies, and operational research studies estimating the diffusion of new tests across European countries and evaluating the implementation of the tests in specific national contexts. Finally, given that diagnostics tests are most often developed for and tested in adults [42] it is important that all of the aforementioned research is conducted with a specific focus on the use of the tests in children.

## Conclusions

There is substantial variability in the adoption of POCTs for the management of acute infections in children across Europe. To inform future implementation of both existing and innovative tests, further research is needed to understand what drives the variation between countries, the needs of frontline clinicians, and the role of diagnostic tests in the management of acute childhood infections.

## Supporting information

S1 Checklist Strobe checklist. (DOCX)

S1 Supplementary materials List of included countries and sample size per country. (DOCX)

S2 Supplementary materials Questionnaire (hospital questionnaire). (DOCX)

S3 Supplementary materials Additional explanations of the multilevel regression modelling. (DOCX)

S4 Supplementary materials Directed acyclic graphs for the inclusion of explanatory variable in the regression models. (DOCX)

S5 Supplementary materials Flow diagram of included observations per analysis. (DOCX)

S6 Supplementary materials Availability of POCTs per country primary care. (DOCX)

S7 Supplementary materials Use of POCTs per country. (DOCX)

## References

[1] Hay AD, Heron J, Ness A. The prevalence of symptoms and consultations in pre-school children in the Avon Longitudinal Study of Parents and Children (ALSPAC): a prospective cohort study. *Family practice*. 2005;22(4):367–74. doi: 10.1093/fampra/cmi035 15897210

[2] Van den Bruel A, Haj-Hassan T, Thompson M, Buntinx F, Mant D. Diagnostic value of clinical features at presentation to identify serious infection in children in developed countries: a systematic review. *Lancet* (London, England). 2010;375(9717):834–45. doi: 10.1016/S0140-6736(09)62000-6 20132979

[3] Nijman RG, Vergouwe Y, Thompson M, van Veen M, van Meurs AHJ, van der Lei J, et al. Clinical prediction model to aid emergency doctors managing febrile children at risk of serious bacterial infections: diagnostic study. *BMJ*: British Medical Journal. 2013;346. doi: 10.1136/bmj.f1706 23550046 PMC3614186

[4] Lucas PJ, Cabral C, Hay AD, Horwood J. A systematic review of parent and clinician views and perceptions that influence prescribing decisions in relation to acute childhood infections in primary care. *Scandinavian journal of primary health care*. 2015;33(1):11–20. doi: 10.3109/02813432.2015.1001942 25716427 PMC4377734

[5] World Health Organization. Global action plan on antimicrobial resistance 2015. Available from https://www.who.int/antimicrobial-resistance/global-action-plan/en/. Accessed 17/05/21. 10.7196/samj.9644 26242647

[6] Cohen JF, Pauchard JY, Hjelm N, Cohen R, Chalumeau M. Efficacy and safety of rapid tests to guide antibiotic prescriptions for sore throat. Cochrane Database Syst Rev. 2020;6(6):Cd012431. doi: 10.1002/14651858.CD012431.pub2 32497279 PMC7271976

[7] Lee JJ, Verbakel JY, Goyder CR, Ananthakumar T, Tan PS, Turner PJ, et al. The Clinical Utility of Point-of-Care Tests for Influenza in Ambulatory Care: A Systematic Review and Meta-analysis. Clin Infect Dis. 2019;69(1):24–33. doi: 10.1093/cid/ciy837 30285232 PMC6579962

[8] Doan Q, Enarson P, Kissoon N, Klassen TP, Johnson DW. Cochrane Review: Rapid viral diagnosis for acute febrile respiratory illness in children in the Emergency Department. Evid Based Child Health. 2010;5(2):709–51. doi: 10.1002/ebch.543 32313519 PMC7163541

[9] Van Hecke O, Raymond M, Lee JJ, Turner P, Goyder CR, Verbakel JY, et al. In-vitro diagnostic point-of-care tests in paediatric ambulatory care: A systematic review and meta-analysis. *PLoS One*. 2020;15(7):e0235605. doi: 10.1371/journal.pone.0235605 32628707 PMC7337322

[10] Verbakel JY, Lee JJ, Goyder C, Tan PS, Ananthakumar T, Turner PJ, et al. Impact of point-of-care C reactive protein in ambulatory care: a systematic review and meta-analysis. BMJ Open. 2019;9(1):e025036. doi: 10.1136/bmjopen-2018-025036 30782747 PMC6361331

[11] Whiting P, Westwood M, Bojke L, Palmer S, Richardson G, Cooper J, et al. Clinical effectiveness and cost-effectiveness of tests for the diagnosis and investigation of urinary tract infection in children: a systematic review and economic model. Health Technol Assess. 2006;10(36):iii-iv, xi-xiii, 1–154. doi: 10.3310/hta10360 17014747

[12] Hollingworth W, Busby J, Butler CC, O’Brien K, Sterne JA, Hood K, et al. The Diagnosis of Urinary Tract Infection in Young Children (DUTY) Study Clinical Rule: Economic Evaluation. Value Health. 2017;20(4):556–66. doi: 10.1016/j.jval.2017.01.003 28407997 PMC5406157

[13] Borensztajn D, Yeung S, Hagedoorn NN, Balode A, von Both U, Carrol ED, et al. Diversity in the emergency care for febrile children in Europe: a questionnaire study. *BMJ Paediatr Open*. 2019;3(1):e000456. doi: 10.1136/bmjpo-2019-000456 31338429 PMC6613846

[14] Hagedoorn NN, Borensztajn DM, Nijman R, Balode A, von Both U, Carrol ED, et al. Variation in antibiotic prescription rates in febrile children presenting to emergency departments across Europe (MOFICHE): A multicentre observational study. *PLoS Med*. 2020;17(8):e1003208. 32813708 10.1371/journal.pmed.1003208 PMC7444592

[15] Engstrom S, Molstad S, Lindstrom K, Nilsson G, Borgquist L. Excessive use of rapid tests in respiratory tract infections in Swedish primary health care. *Scand J Infect Dis*. 2004;36(3):213–8. doi: 10.1080/00365540310018842 15119368

[16] Andre M, Vernby A, Odenholt I, Lundborg CS, Axelsson I, Eriksson M, et al. Diagnosis-prescribing surveys in 2000, 2002 and 2005 in Swedish general practice: consultations, diagnosis, diagnostics and treatment choices. *Scand J Infect Dis*. 2008;40(8):648–54. doi: 10.1080/00365540801932439 18979603

[17] Neumark T, Brudin L, Molstad S. Use of rapid diagnostic tests and choice of antibiotics in respiratory tract infections in primary healthcare—a 6-y follow-up study. *Scand J Infect Dis*. 2010;42(2):90–6. doi: 10.3109/00365540903352932 19902992

[18] Howick J, Cals JW, Jones C, Price CP, Pluddemann A, Heneghan C, et al. Current and future use of point-of-care tests in primary care: an international survey in Australia, Belgium, The Netherlands, the UK and the USA. *BMJ Open*. 2014;4(8):e005611. doi: 10.1136/bmjopen-2014-005611 25107438 PMC4127935

[19] Lindstrom J, Nordeman L, Hagstrom B. What a difference a CRP makes. A prospective observational study on how point-of-care C-reactive protein testing influences antibiotic prescription for respiratory tract infections in Swedish primary health care. *Scandinavian journal of primary health care*. 2015;33(4):275–82. doi: 10.3109/02813432.2015.1114348 26643196 PMC4750737

[20] Tyrstrup M, Beckman A, Molstad S, Engstrom S, Lannering C, Melander E, et al. Reduction in antibiotic prescribing for respiratory tract infections in Swedish primary care- a retrospective study of electronic patient records. *BMC infectious diseases*. 2016;16(1):709. doi: 10.1186/s12879-016-2018-9 27887585 PMC5124268

[21] Haldrup S, Thomsen RW, Bro F, Skov R, Bjerrum L, Sogaard M. Microbiological point of care testing before antibiotic prescribing in primary care: considerable variations between practices. *BMC family practice*. 2017;18(1):9. doi: 10.1186/s12875-016-0576-y 28125965 PMC5270219

[22] Merlo J, Wagner P, Ghith N, Leckie G. An Original Stepwise Multilevel Logistic Regression Analysis of Discriminatory Accuracy: The Case of Neighbourhoods and Health. *PLoS One*. 2016;11(4):e0153778. doi: 10.1371/journal.pone.0153778 27120054 PMC4847925

[23] Williams TC, Bach CC, Matthiesen NB, Henriksen TB, Gagliardi L. Directed acyclic graphs: a tool for causal studies in paediatrics. *Pediatr Res*. 2018;84 **(**4):487–93. doi: 10.1038/s41390-018-0071-3 29967527 PMC6215481

[24] Spiegelhalter DJ, Best NG, Carlin BP, van der Linde A. The deviance information criterion: 12 years on. *Journal of the Royal Statistical Society: Series B (Statistical Methodology)*. 2014;76(3):485–93.

[25] OECD. Organisation for Economic Co-operation and Development. Health at a glance. Chapter 9. Available from https://www.oecd-ilibrary.org/social-issues-migration-health/health-at-a-glance-2019_4dd50c09-en. Accessed 17/05/21.

[26] Wadge H., Roy R., Sripathy A., Prime M., Carter A., Fontana G., et al. Evaluating the impact of private providers on health and health systems. London, UK: Imperial College London, 2017. Available from https://assets.cdcgroup.com/wp-content/uploads/2017/06/25150846/Impact-of-private-providers-on-health-and-health-systems.pdf. Accessed 17/05/21.

[27] Lenzer J. Unnecessary care: are doctors in denial and is profit driven healthcare to blame? *Bmj*. 2012;345:e6230. doi: 10.1136/bmj.e6230 23033411

[28] Hadjipanayis A, Grossman Z, Del Torso S, van Esso D, Dornbusch HJ, Mazur A, et al. Current primary care management of children aged 1–36 months with urinary tract infections in Europe: large scale survey of paediatric practice. *Archives of disease in childhood*. 2015;100(4):341–7. doi: 10.1136/archdischild-2014-306119 25378379

[29] Cornaglia C, Robinet J, Partouche H. [Use of Rapid Antigen Detection Test (RADT) among general practitioner teachers at the Paris Descartes University: 2005–2007]. *Med Mal Infect*. 2009;39(6):375–81. 19345529 10.1016/j.medmal.2009.02.011

[30] Martín Peinador Y, Albañil Ballesteros MR, García Vera C, Jimenez Alés R, Muñoz Hiraldo E, Martínez Chamorro MJ. [Access to complementary tests for the diagnosis of infectious diseases in primary care paediatric clinics]. *An Pediatr (Barc)*. 2021;94(2):82–91. 10.1016/j.anpedi.2020.03.015 32430217

[31] Greenhalgh T, Wherton J, Papoutsi C, Lynch J, Hughes G, A’Court C, et al. Beyond Adoption: A New Framework for Theorizing and Evaluating Nonadoption, Abandonment, and Challenges to the Scale-Up, Spread, and Sustainability of Health and Care Technologies. J Med Internet Res. 2017;19(11):e367. doi: 10.2196/jmir.8775 29092808 PMC5688245

[32] Verbakel JY, Turner PJ, Thompson MJ, Plüddemann A, Price CP, Shinkins B, et al. Common evidence gaps in point-of-care diagnostic test evaluation: a review of horizon scan reports. *BMJ Open*. 2017;7(9):e015760. doi: 10.1136/bmjopen-2016-015760 28864692 PMC5588931

[33] World Health Organization. European observatory on Health Systems and Policies. Ensuring value for money in health care. 2008. Available from https://www.euro.who.int/__data/assets/pdf_file/0011/98291/E91271.pdf. Accessed 17/05/21.

[34] World Health Organization. European observatory on Health Systems and Policies. Health Technology Assessment and Health Policy-Making in Europe. 2008. Available from https://apps.who.int/iris/handle/10665/107911. Accessed 17/05/21.

[35] Li E, Dewez JE, Luu Q, Emonts M, Maconochie I, Nijman R, et al. Role of point-of-care tests in the management of febrile children: a qualitative study of hospital-based doctors and nurses in England. BMJ Open. 2021;11(5):e044510. doi: 10.1136/bmjopen-2020-044510 33972339 PMC8112413

[36] Esposito S, Mencacci A, Cenci E, Camilloni B, Silvestri E, Principi N. Multiplex Platforms for the Identification of Respiratory Pathogens: Are They Useful in Pediatric Clinical Practice? Frontiers in Cellular and Infection Microbiology. 2019;9. 31275863 10.3389/fcimb.2019.00196 PMC6593267

[37] Herberg JA, Kaforou M, Wright VJ, Shailes H, Eleftherohorinou H, Hoggart CJ, et al. Diagnostic Test Accuracy of a 2-Transcript Host RNA Signature for Discriminating Bacterial vs Viral Infection in Febrile Children. Jama. 2016;316(8):835–45. doi: 10.1001/jama.2016.11236 27552617 PMC5997174

[38] PERFORM. Personalised Risk assessment in febrile illness to optimise Real-life Management across the European Union. Available from https://www.perform2020.org. Accessed 12/08/22.

[39] DIAMONDS. Diagnosis and Management of Febrile Illness using RNA Personalised Molecular Signature Diagnosis. Available from https://www.diamonds2020.eu. Accessed 12/08/22.

[40] Mejias A, Cohen S, Glowinski R, Ramilo O. Host transcriptional signatures as predictive markers of infection in children. Curr Opin Infect Dis. 2021;34(5):552–8. doi: 10.1097/QCO.0000000000000750 34232136 PMC8446306

[41] Horvath AR, Lord SJ, StJohn A, Sandberg S, Cobbaert CM, Lorenz S, et al. From biomarkers to medical tests: The changing landscape of test evaluation. Clinica Chimica Acta. 2014;427:49–57. doi: 10.1016/j.cca.2013.09.018 24076255

[42] Caldwell CS, Denne SC. Rigorous and consistent evaluation of diagnostic tests in children: another unmet need. Pediatr Res. 2020;88(4):524–5. doi: 10.1038/s41390-020-01110-0 32892214 PMC7493055

