# Supplementary material for: Availability and use of rapid diagnostic tests for the management of acute childhood infections in Europe: A cross-sectional survey of paediatricians
Source: PLoS One. 2022 Dec 20;17(12):e0275336. doi: 10.1371/journal.pone.0275336 (PMC9767335; doi:10.1371/journal.pone.0275336)
Supplement: S1 Supplementary materials — (DOCX) [file pone.0275336.s002.docx]

# **S1 Supplementary Materials: List of included countries and sample size per country**

| **Table 1. Sample sizes to estimate the main outcomes (current availability of CRP POCT, and use of CRP POCT in a clinical scenario) with 90% confidence, a margin of error below 10%, and an expected proportion of the outcomes of 50%** | | | | |
| --- | --- | --- | --- | --- |
| **Country** | **Total population of primary care paediatricians^*^** | **Sample size of primary care paediatricians** | **Total population of hospital paediatricians^*^** | **Sample size of hospital paediatricians** |
| **Austria** | 585 | 61 | 774 | 62 |
| **Belgium** | 782 | 65 | 781 | 65 |
| **Bulgaria** | NA | NA | 1,475 | 65 |
| **Croatia** | 281 | 55 | 583 | 61 |
| **Cyprus** | 180 | 49 | 68 | 34 |
| **Czech Rep.** | 753 | 62 | 669 | 61 |
| **Denmark** | NA | NA | 469 | 59 |
| **Finland** | 73 | 35 | 623 | 61 |
| **France** | 1,453 | 65 | 6,622 | 67 |
| **Germany** | 5,991 | 67 | 7,924 | 67 |
| **Greece** | 2,128 | 65 | 2,130 | 65 |
| **Hungary** | 939 | 63 | 1,432 | 65 |
| **Ireland** | NA | NA | 451 | 59 |
| **Israel** | 501 | 60 | 1,699 | 65 |
| **Italy** | 6,000 | 67 | 11,354 | 67 |
| **Latvia** | 10 | 9 | 238 | 53 |
| **Lithuania** | 40 | 25 | 676 | 61 |
| **Malta** | NA | NA | 81 | 37 |
| **The Netherlands** | NA | NA | 1,751 | 65 |
| **Norway** | NA | NA | 875 | 63 |
| **Poland** | 5,040 | 67 | 9,905 | 67 |
| **Portugal** | NA | NA | 2,085 | 66 |
| **Romania** | NA | NA | 2,655 | 66 |
| **Slovenia** | 252 | 53 | 396 | 58 |
| **Spain** | 4,800 | 67 | 7,589 | 67 |
| **Sweden** | NA | NA | 1083 | 64 |
| **Switzerland** | 978 | 63 | 839 | 63 |
| **Ukraine** | 3,321 | 66 | 6,236 | 67 |
| **United Kingdom** | NA | NA | 10,464 | 67 |
| **TOTAL** | 34,107 | 1,064 | 81,927 | 1,787 |

*Source: Eurostat 2019 (available from [https://appsso·eurostat·ec·europa·eu/nui/show·do?dataset=hlth_rs_phys&lang=en](https://appsso.eurostat.ec.europa.eu/nui/show.do?dataset=hlth_rs_phys&lang=en), accessed 03/12/19) and European Confederation of Primary Care Paediatricians 2018 (ECPCP, available from: [https://www·ecpcp·eu](https://www.ecpcp.eu), accessed 10/10/19), except for Spain and Poland, where figures were not available and provided by local partners.

NA: not applicable

In addition to the sample size estimates provided in the main manuscript, we also assessed whether these sample sizes would also allow identification of determinants of the main outcomes of interest with sufficient statistical power in multiple logistic regression analyses. Based on a rule of thumb of doubling the sample size to allow for multivariable analyses, we considered that if half of the sample sizes in Table 1 would allow detection of a difference in the main outcomes of interest between categories of the main hypothesised explanatory variables (health expenditure per capita for CRP POCT availability, and years of clinical experience for CRP POCT use), with >90% power, then the full samples sizes presented in Table 1 would also be sufficient for the regression analyses. With regards the determinants of CRP POCT availability, we grouped countries into two categories of health expenditure per capita (HEC): category 1 grouped countries spending ≤2,800 Euros per capita and category 2 countries spending >2,800 Euros, as 2,800 Euros is the median HEC of the countries included in the study^1,2^ (Table 2)· We hypothesised that CRP POCT would be available to 50% of clinicians in the >2,800 Euros group based on the published availability of CRP POCT in the the Netherlands,^3^ compared to 25% in the ≤2,800 Euros. The power to detect a difference between the two groups (with 283 primary care paediatricians in the ≤2,800 Euros category versus 241 in the >2,800 category, and 407 hospital paediatricians in the ≤2,800 Euros category versus 475 in the >2,800category, Table 2) would be 100% in both primary care and hospital settings· With regards the determinants of CRP POCT use in the clinical scenario, we grouped participants into two categories: category 1 grouped participants with ≤ 10 years of experience, category 2 participants with >10 years of experience.^4^ We considered that 20% of the sample will have ≤10 years of experience, based on European figures of years of experience of medical doctors.^5^ We hypothesised that less experienced paediatricians would use CRP POCT in 45% of patients in the clinical scenario, while more experienced paediatricians will do so in 25% of patients, based on the rate of CRP use in febrile infants from 12 European hospitals members of the PERFORM consortium.^6^ The power to detect a difference between the two groups (with 105 primary care paediatricians in the ≤10 years of experience category versus 419 in the >10 years of experience category, and 176 hospital paediatricians in the ≤10 years of experience category versus 706 in the >10 years of experience category, Table 3) would be 97% in primary care and 100% in hospital settings. Thus, we were confident that the sample sizes in table 1 would ensure that the planned regression analyses have sufficient power.

| **Table 2· Expected number of participants and health expenditure per capita categories** | | | | | | |
| --- | --- | --- | --- | --- | --- | --- |
| **Country** | **Health expenditure per capita per year category (Euros)** | | **Half of primary care paediatricians’ sample size** | | **Half of hospital paediatricians’ sample size** | |
| Bulgaria | ≤2,800 | | NA | | 32 | |
| Croatia |  |  | 27 | | 30 | |
| Cyprus |  |  | 24 | | 17 | |
| Czech Rep· |  |  | 31 | | 30 | |
| Greece |  |  | 32 | | 32 | |
| Hungary |  |  | 31 | | 32 | |
| Israel |  |  | 30 | | 32 | |
| Latvia |  |  | 4 | | 26 | |
| Lithuania |  |  | 12 | | 30 | |
| Malta |  |  | NA | | 18 | |
| Poland |  |  | 33 | | 33 | |
| Romania |  |  | NA | | 33 | |
| Slovenia |  |  | 26 | | 29 | |
| Ukraine |  |  | 33 | | 33 | |
| **Sub total** |  |  | **283** | | **407** | |
| Austria | >2,800 | | 30 | | 31 | |
| Belgium |  |  | 32 | | 32 | |
| Denmark |  |  | NA | | 29 | |
| Finland |  |  | 17 | | 30 | |
| France |  |  | 32 | | 33 | |
| Germany |  |  | 33 | | 33 | |
| Italy |  |  | 33 | | 33 | |
| Ireland |  |  | NA | | 29 | |
| The Netherlands |  |  | NA | | 32 | |
| Norway |  |  | NA | | 31 | |
| Portugal |  |  | NA | | 33 | |
| Spain |  |  | 33 | | 33 | |
| Switzerland |  |  | 31 | | 31 | |
| Sweden |  |  | NA | | 32 | |
| United Kingdom |  |  | NA | | 33 | |
| **Subtotal** |  |  | **241** | | **475** | |
| **TOTAL** |  | | **524** | | **882** | |
| NA: not applicable |  | |  | |  | |
|  |  | |  | |  | |
| **Table 3· Expected number of participants and years of clinical experience** | | | | | |  |
| **Years of clinical experience** | | **Half of primary care paediatricians’ sample size (all countries)** | | **Half of hospital paediatricians’ sample size (all countries)** | |  |
| **Any experience** | | 524 | | 882 | |  |
| **<10 years of practice (20% of any experience)^37^** | | 105 | | 176 | |  |
| **>10 years of practice (80% of any experience)^37^** | | 419 | | 706 | |  |

**References**

1. OECD. Organisation for Economic Co-operation and Development. Health at a glance. Europe 2016· State of health in the EU cycle. Available from [http://www·oecd·org/health/health-at-a-glance-europe-23056088·htm](http://www.oecd.org/health/health-at-a-glance-europe-23056088.htm) · Accessed 03/12/19.
2. WHO. World Health Organization. Available from: [https://www·who·int/countries/en/](https://www.who.int/countries/en/) . Accessed 03/12/19.
3. Howick J, Cals JW, Jones C, Price CP, Pluddemann A, Heneghan C, et al. Current and future use of point-of-care tests in primary care: an international survey in Australia, Belgium, The The Netherlands, the UK and the USA· BMJ Open· 2014;4(8):e005611.
4. McGillivray DL, Roberts-Brauer R, Kramer MS· Diagnostic test ordering in the evaluation of febrile children. Physician and environmental factors· American journal of diseases of children (1960)· 1993;147(8):870-4.
5. Eurostat 2019· Available from [https://appsso·eurostat·ec·europa·eu/nui/show·do?dataset=hlth_rs_phys&lang=en](https://appsso.eurostat.ec.europa.eu/nui/show.do?dataset=hlth_rs_phys&lang=en)

Accessed 03/12/19.

1. Hagedoorn NN, Borensztajn DM, Nijman R, Balode A, von Both U, Carrol ED, et al· Variation in antibiotic prescription rates in febrile children presenting to emergency departments across Europe (MOFICHE): A multicentre observational study. PLoS Med· 2020;17(8):e1003208.
