# Supplementary material for: Availability and use of rapid diagnostic tests for the management of acute childhood infections in Europe: A cross-sectional survey of paediatricians
Source: PLoS One. 2022 Dec 20;17(12):e0275336. doi: 10.1371/journal.pone.0275336 (PMC9767335; doi:10.1371/journal.pone.0275336)
Supplement: S2 Supplementary materials — (DOCX) [file pone.0275336.s003.docx]

# **S2 Supplementary Materials: questionnaire (hospital questionnaire)**

| **THE USE OF RAPID POINT-OF-CARE TESTS FOR MANAGING ACUTE CHILDHOOD INFECTIONS IN EUROPE** | | | | |
| --- | --- | --- | --- | --- |
| **PARTICIPANT INFORMATION AND CONSENT FORM** | | | | |
| The aim of the study is to estimate and compare the availability and use of rapid point-of-care tests for the management of acute childhood infections across Europe. **Rapid point-of-care tests are tests that are carried out in the consultation room (or in an adjacent room), with results available within the consultation timeframe.** | | | | |
| The study is conducted by the European Academy of Paediatrics Research in Ambulatory Settings Network (EAPRASnet) and researchers from the London School of Hygiene and Tropical Medicine (LSHTM). EAPRASnet is a practice-based research network whose mission is to improve the health of European children n and enhance the quality of primary care paediatrics· The LSHTM is a university specialised in global and public health research. The study is part of PERFORM, a large research collaboration which aims to develop new tests to for management of children with febrile illness. | | | | |
| We are recruiting physicians from across Europe who regularly treat children with acute infections. | | | | |
| The survey should take approximately 10 minutes to complete. | | | | |
| Your participation will help understand how physicians use rapid point-of-care tests in children with infections. We will ask questions on the availability of these tests; we will then ask you to comment on a clinical scenario; and we will finish with questions regarding future diagnostic tests. | | | | |
| There are no risks in taking part in the survey. The aim of the survey is not to assess the quality of your work.  All information collected in this study will be anonymised and kept confidential. Your responses will be combined with those of other respondents in reports and potential publications in peer-reviewed medical journals. Your response may be used for future research use as well. LSHTM will keep the information you provided for 10 years after the study has finished· You can withdraw from the study at any time without providing a reason. | | | | |
| Please tick the box below to confirm that you consent to the data being used in the way described here. If you have any questions regarding this survey, please contact: [perform2020@lshtm·ac·uk](mailto:perform2020@lshtm.ac.uk) or [info@eaprasnet·org](mailto:info@eaprasnet.org) | | | | |
| □ YES, I have read and consent to the data being used in the way described above | | | | |
| **SECTION A: GENERAL INFORMATION ON RESPONDENT AND SETTING** | | | | |
| **A00 Which professional societies/networks are you a member of? TICK ALL THAT APPLY** | | | | |
| □ I am not a member of any professional societies/networks | | | | |
| □ The national society of paediatrics | | | | |
| □ A sub-national (e·g· regional) society of paediatrics | | | | |
| □ A national society of paediatric emergency medicine | | | | |
| □ A national society of paediatric infectious diseases | | | | |
| □ A national society of primary care paediatrics | | | | |
| □ A national society of junior paediatricians | | | | |
| □ A national society of general practice/family medicine | | | | |
| □ European Academy of Paediatrics-EAP | | | | |
| □ EAP Research in Ambulatory Settings Network-EAPRASnet | | | | |
| □ European Society of Paediatric Infectious Diseases-ESPID | | | | |
| □**Other(s), including other European societies, please specify:** | | | | |
| **-------------------------------------------------------------------------------------------------------------------** | | | | |
| **A01 What is your job? TICK ONE BOX** | | | | |
| □ Paediatric trainee **GO TO QUESTION A03** | | | | |
| □ General paediatrician **GO TO QUESTION A03** | | | | |
| □ Paediatrician with subspecialty or special interest **GO TO QUESTION A02** | | | | |
| □ Emergency doctor for adults AND children **GO TO QUESTION A03** | | | | |
|  | | | | |
| **A02 Please indicate your main subspecialty or special interest: TICK ONE BOX** | | | | |
| □ Allergy | | | | |
| □ Cardiology | | | | |
| □ Endocrinology | | | | |
| □ Emergency medicine | | | | |
| □ Gastroenterology | | | | |
| □ Immunology / Infectious diseases | | | | |
| □ Intensive care | | | | |
| □ Neonatology | | | | |
| □ Nephrology | | | | |
| □ Neurology | | | | |
| □ Neurodevelopment | | | | |
| □ Oncology | | | | |
| □ Respiratory paediatrics | | | | |
| □ Rheumatology | | | | |
| □ **Other, please specify:** | | | | |
| **-------------------------------------------------------------------------------------------------------------------** | | | | |
| **A03 What year did you qualify from medical school (before starting the paediatric or emergency medicine training)?** | | | | |
| **-------------------------------------------------------------------------------------------------------------------** | | | | |
| **About your main workplace** | | | | |
| **A04 Are most of your patients from an…? TICK ONE BOX** | | | | |
| □ Urban area | | | | |
| □ Rural area | | | | |
| □ Mixed urban-rural area | | | | |
| **A05 Is your work primarily in the private or public sector? TICK ONE BOX** | | | | |
| □ Private | | | | |
| □ Public | | | | |
| **A06 In which hospital department do you mainly work? TICK ONE BOX** | | | | |
| □ Outpatient department | | | | |
| □ Paediatric Emergency Department | | | | |
| □ Emergency Department (for adults & children) | | | | |
| □ Paediatrics ward | | | | |
| □ **Other, please specify**: | | | | |
| **-------------------------------------------------------------------------------------------------------------------** | | | | |
| **A07 Which of the following best describes your hospital? TICK ONE BOX** | | | | |
| □ Hospital providing secondary care only | | | | |
| □ Hospital providing secondary, and tertiary care | | | | |
| **A08 Which of the following best describes your hospital? TICK ONE BOX** | | | | |
| □ Paediatric or women and children hospital | | | | |
| □ General hospital | | | | |
| **A09 Who usually takes blood for routine tests, like C-reactive protein or full blood count? TICK ONE BOX** | | | | |
| □ Nurses | | | | |
| □ Doctors | | | | |
| □ Laboratory technicians | | | | |
| □ Phlebotomists or others | | | | |
| **A10 What is the shortest turnaround time to get results of blood tests such as C-reactive protein or full blood count sent to the hospital lab?** | | | | |
| **-------------------------------------------------------------------------------------------------------------------** | | | | |
| **A11 What is your hospital’s name and city?** | | | | |
| This question is not mandatory; it will help us knowing whether participants are from the same or different institutions· The name of the hospital will not be used in reports | | | | |
| **-------------------------------------------------------------------------------------------------------------------** | | | | |
| **A12 On average, how long are your consultations during busier times of the year (in minutes)?** | | | | |
| **-------------------------------------------------------------------------------------------------------------------** | | | | |
| **SECTION B: AVAILABILITY AND USE OF RAPID POINT-OF-CARE TESTS FOR THE CLINICAL MANAGEMENT OF INFECTIONS IN CHILDREN** | | | | |
| This section assesses the general availability and use of rapid point-of-care tests (POCTs), regardless of who pays for it. Rapid point-of-care tests are tests performed in the consultation room, or in another room in your department, with results available during the consultation timeframe. | | | | |
| **Which of the following diagnostic tests are usually available as POCTs in your workplace? If not available, please indicate if you would like the test for use in children TICK ONE BOX PER ROW** | | | | |
| **RAPID POINT-OF-CARE TESTS (POCT)** | This POCT is **available and I do use** it in children | This POCT is **available, but rarely used** in children | This POCT is **NOT available; I would like it** available | This POCT is  **NOT available; I don’t think I need it** |
| B01 **C-Reactive protein** | □ | □ | □ | □ |
| B02 **Procalcitonin** | □ | □ | □ | □ |
| B03 **Full blood cell count or white blood cell count** | □ | □ | □ | □ |
| B04 **Blood gas**  **(with or without lactate)** | □ | □ | □ | □ |
| B05 **Lactate alone** | □ | □ | □ | □ |
| B06 **Urine dipstick** | □ | □ | □ | □ |
| B06·2 **Microscopy in the clinical area*** | □ | □ | □ | □ |
| *In some hospitals, microscopes are available on the ward and clinicians look at urines directly using the microscope· In that case the microscope is a rapid point-of-care test· | | | | |
| B07 **Influenza virus rapid antigen** | □ | □ | □ | □ |
| B08 **Respiratory Syncytial Virus rapid antigen** | □ | □ | □ | □ |
| B09 **Group A streptococci rapid antigen (throat swab)** | □ | □ | □ | □ |

| **B10 Are there other rapid point-of-care tests that are available to you in your workplace, for the clinical management of infections in children? TICK ONE BOX** |
| --- |
| □ No |
| □ Yes, specify: |
| **SECTION C: CLINICAL SCENARIO** |
| A 4-month old infant is brought to see you in the early evening during busier times of the year. His parents report that he has had fever since the early morning with temperatures of 38° C measured on 2 occasions. He has not been feeding well. He has not been in contact with sick people. |
| He is up to date with vaccination· He has not received a vaccination in the last 48 hours. |
| His axillary temperature is 38·6° C, heart rate: 140/ min, respiratory rate 40/min. He appears well, he is alert, has warm extremities, and the rest of the physical examination is normal. There is no clear focus of infection. |
| **C01 What do you think is the probability that he has a bacterial infection? TICK ONE BOX** |
| □ I don't know |
| □ ≥80% probability |
| □ 60-79% probability |
| □ 40-59% probability |
| □ 20-39% probability |
| □ 10-19% probability |
| □ <10% probability |
| **C02 In your workplace, would you carry out any available diagnostic test (including blood, urine, respiratory, or other tests) in this patient, as part of your initial assessment? TICK ONE BOX** |
| In this question, we are interested in both rapid point-of-care tests and hospital lab-based tests |
| □ Yes **GO TO QUESTION C03** |
| □ No **GO TO QUESTION C09** |
| **C03 Would you prescribe antibiotics? TICK ONE BOX** |
| □ Yes, definitely |
| □ No, definitely |
| □ It will depend on the diagnostic test results |
| **C04 Would you admit the patient? TICK ONE BOX** |
| □ Yes, definitely |
| □ No, definitely |
| □ It will depend on the diagnostic test results |

| **How important to you are the following reasons for carrying out a diagnostic test in this patient?**  **TICK ONE BOX PER ROW** | | | | | | | |
| --- | --- | --- | --- | --- | --- | --- | --- |
|  | Not at all important | Slightly important | Moderately important | Important | Fairly important | Very important | Absolutely essential |
| C05 **To help deciding whether to prescribe antibiotics** | □ | □ | □ | □ | □ | □ | □ |
| C06 **To help deciding whether to admit the patient** | □ | □ | □ | □ | □ | □ | □ |
| C07 **To help deciding whether the patient needs urgent medical assessment or can be reassessed later on** | □ | □ | □ | □ | □ | □ | □ |
| C08 **To reassure parents** | □ | □ | □ | □ | □ | □ | □ |

**GO TO SECTION D**

| **C09 Would you prescribe antibiotics? TICK ONE BOX** |
| --- |
| □ Yes |
| □ No |
| **C10 Would you observe, admit or discharge the patient? TICK ONE BOX** |
| □ I would observe the patient for a few hours |
| □ I would admit the patient to the inpatient ward |
| □ I would discharge the patient |
| **C11 Why would you NOT use diagnostic tests in this patient as part of your initial assessment? TICK ONE BOX** |
| □ Tests that I would like to use are not available **GO TO QUESTION C12** |
| □ Tests would not change my clinical management **GO TO SECTION D** |
| □ I would first observe the patient for few hours **GO TO SECTION D** |
| □ **Other, please specify** |
| **-------------------------------------------------------------------------------------------------------------------**  **AND GO TO SECTION D** |

| **C12 Assuming all the following tests are available in your workplace, please indicate which test you would use in this clinical scenario, and which version (POCT and/or lab version).**  **TICK ONE BOX PER ROW** | | | | |
| --- | --- | --- | --- | --- |
| DIAGNOSTIC TESTS | I would not use this test | I would use the POCT version | I would use the lab version | I would use both the POCT and lab versions |
| C12·1 **C-Reactive protein** | □ | □ | □ | □ |
| C13 **Procalcitonin** | □ | □ | □ | □ |
| C14 **Full blood cell count or white blood cell count** | □ | □ | □ | □ |
| C15 **Blood gas**  **(with or without lactate)** | □ | □ | □ | □ |
| C16 **Lactate alone** | □ | □ | □ | □ |
| C16·2 **Blood culture** | □ |  | □ |  |
| C17 **Urine microscopy and/or nitrites** | □ | □ | □ | □ |
| C18 **Urine culture and sensitivity** | □ |  | □ |  |
| C19 **Urine dipstick** | □ | □ | □  Urine dipstick are sometime done in the lab | □ |
| C20 **Influenza** | □ | □ | □ | □ |
| C21 **Respiratory Syncytial Virus** | □ | □ | □ | □ |
| C21·2 **CSF metrics, culture, sensitivity (+- PCR for herpes simplex or other viruses)** | □ |  | □ |  |

| **C22 Other tests· Please specify:** |
| --- |
| **-------------------------------------------------------------------------------------------------------------------** |

| **SECTION D: PREFERENCE OF RAPID POCTS VS LAB TESTS, AND CHARACTERISTICS OF FUTURE TESTS** |
| --- |
| In the following section, we are interested in your opinion about using laboratory tests versus rapid point-of- care tests in general, i·e· NOT specifically related to the clinical scenario described earlier· We will use C-reactive protein (CRP) as an example. |
| **D01 Do you think C-reactive protein has any role in the management of children with a suspicion of infection? TICK ONE BOX** |
| □ Yes **GO TO QUESTION D02** |
| □ No **GO TO QUESTION D03** |
| **D02 If both rapid point-of-care and laboratory versions of C-reactive protein were available, which version would you prefer generally? TICK ONE BOX** |
| □ I would prefer the rapid point-of-care version |
| □ I would prefer the laboratory version |
| □ I would use both tests |
| □ I do not know |
| **The next three questions are about your opinion about the need for new blood-based diagnostic tests for the management of children with acute infection·** |
| **D03 Do you think new tests are needed? TICK ONE BOX** |
| □ Yes **GO TO QUESTION D04** |
| □ No **GO TO QUESTION D13** |

| **D04 How important to you would be the following purposes of the new tests?**  **TICK ONE BOX PER ROW** | | | | | | | |
| --- | --- | --- | --- | --- | --- | --- | --- |
|  | Not at all important | Slightly important | Moderately important | Important | Fairly important | Very important | Absolutely essential |
| D04·1 **To predict risk of developing severe disease (regardless of causative pathogen)** | □ | □ | □ | □ | □ | □ | □ |
| D05 **To indicate the presence/absence of any bacterial infection** | □ | □ | □ | □ | □ | □ | □ |
| D06 **To identify specific bacterial infections** | □ | □ | □ | □ | □ | □ | □ |
| D07 **To indicate sensitivity to antibiotics** | □ | □ | □ | □ | □ | □ | □ |
| D08 **To indicate the presence/absence of any viral infection** | □ | □ | □ | □ | □ | □ | □ |

| **D09 Are there other purposes that are important to you? TICK ONE BOX** |
| --- |
| □ No |
| □ Yes |
| **D10 If Yes, specify:** |
| **-------------------------------------------------------------------------------------------------------------------** |
| **D11 What is the maximum time to get results beyond which you would not want to use the new tests?** |
| **-------------------------------------------------------------------------------------------------------------------** |
| **D12 If a new finger prick test became available that differentiates bacterial from viral infections with high sensitivity and specificity and reasonable cost, how willing would you be to use the test? TICK ONE BOX** |
| □ I would be among the first to want to use it |
| □ I would use it but only after few of my peers used it |
| □ I would want to use it if it became common practice |
| □ I am unlikely to use such a test |
| □ I don't know |
| **Thank you for your participation!** |
