# Supplementary material for: Availability and use of rapid diagnostic tests for the management of acute childhood infections in Europe: A cross-sectional survey of paediatricians
Source: PLoS One. 2022 Dec 20;17(12):e0275336. doi: 10.1371/journal.pone.0275336 (PMC9767335; doi:10.1371/journal.pone.0275336)
Supplement: S3 Supplementary materials — (DOCX) [file pone.0275336.s004.docx]

# **S3 Supplementary Materials: additional explanations of the multilevel regression modelling**

Multilevel logistic regressions are useful because they allow separation of the effect, on the outcome of interest, of specific characteristics of individual observations from the effect of a setting or context, that is shared by several observations (often called ‘clusters’). We used the mixed-effects approach developed by Merlo and colleagues,^1^ based on the stepwise use of fixed effects to measure the effect of specific characteristics, and random effects to measure the effect of a setting/context.

## Multilevel analysis to identify determinants of the availability of POCTs

In the analysis to identify determinants of the availability of POCTs, each workplace (primary care practice or hospital) is an individual observation. Each observation has specific characteristics that vary between observations (for example some hospitals are private, while others are public). Each cluster (in this study, each country) share many common characteristics, some measured (i·e·, health expenditure per capita, and main financing scheme) but many unmeasured (for example laws regulating the use of commercial advertisement to promote POCTs, or national clinical guidelines). The country characteristics are the same for all observations from the same country.

To separate the effect on the availability of POCTs of specific workplace characteristics from the effect of country as a whole, we used the following mixed-effects stepwise approach: In Model 1, specific workplace characteristics were analysed using fixed effects. This model is a base that was used as a comparator of Model 2· In Model 2 we added a second level of analysis, the country of work, as a random effect (Table 4). One of the benefits of a random effect is that it allows incorporating both measured and unmeasured country characteristics. It also allows the country of work to vary while the workplace characteristics are fixed, which in turn allows understanding of the relative contributions of workplace characteristics and of country as a whole to the availability of POCTs. We assessed these contributions through the change in the area under the receiver operating characteristic curve (AUC) and the median odds ratio (MOR). The change in AUC between models 1 and 2 quantifies the added value of having information on the country as a whole when it comes to identifying the availability of POCTs· The MOR quantifies the magnitude of the random effect variance of country as a whole. The MOR is the median value of the distribution of ORs obtained when randomly picking two workplaces with the same characteristics from two different countries and comparing the one with the higher availability of POCT to the one with the lower availability· If the MOR= 1, this means that there is no variation in the outcome across countries. If the MOR> 1, there is variation across countries, and the larger the MOR the larger the variation is.

In Model 3, we sought to identify characteristics through which the country effect as a whole occurs by adding specific measured country characteristics to the model. As in models 1 and 2, the workplace characteristics were used as fixed effects, to which we added two measured country characteristics, health expenditure per capita and main financing scheme (also as fixed effects), while keeping the remaining unmeasured country characteristics as random effects. We used the 80% interval odds ratio (IOR80) to measure the fixed effects of the country characteristics· Using traditional odd ratios (ORs) for variables varying on the cluster level is incorrect, because in this case ORs only allow comparison of workplaces within a cluster (the country) but not across clusters. The IOR80 overcomes this limitation· The IOR80 is defined as the middle 80% range of the distribution of ORs formed by making random pairwise comparisons between workplaces with identical characteristics but differing on one of the country-specific characteristics (an example of this would be a comparison between a workplace from a country where the main financing scheme is through government funding, with another workplace that is identical except that it is located in a country where the financing scheme is through social health insurance). The IOR80 interval is narrow if the between-country variability is small, and it is wide if the between-country variability is large. If the IOR80 interval contains one, the variability of the country as a whole is large in comparison with the effect of the country characteristic, and the effect of the country characteristic is considered as minor. If the IOR does not contain one, then the effect of the country characteristic is large in comparison with the variability of the country as a whole, and the country characteristic is considered as important. Model 3 was also used to assess the effect of workplace characteristics adjusting for county as a whole for the two measured country characteristics, as these combined adjustments were not done in Models 1 and 2.

All the variables considered in these analyses were identified through direct acyclic graph (see section 4).

| **Table 4· The three models for the multilevel regression analysis on the availability of POCTs** | | | |
| --- | --- | --- | --- |
|  | **Model 1** | **Model 2** | **Model 3** |
| **Purpose** | Base model to be used as a comparator when assessing the effect of adding country as a whole | To assess the effect of adding country as a whole to Model 1· This allows assessment of the effect of workplace characteristics, and of country as a whole to the availability of POCTs | 1·To assess the effect of country specific characteristics·  2·To assess the effect of workplace characteristics adjusted for country as a whole and for country specific characteristics |
| **Measure of assessment** | - | 1· Change in area under the curve  2· Median odds ratios | 1· 80% interval odds ratios (for country characteristics)  2· Odds ratios (for workplace characteristics) |
| **Analysis of primary care practices** |  |  |  |
| **Levels** |  |  |  |
| Workplace (Primary care practice) | X | X | X |
| Country of work |  | X | X |
| **Primary care practice characteristics** |  |  |  |
| Sector of activity (private/public) | X | X | X |
| Practice size (solo/ group practice) | X | X | X |
| Turnaround time for diagnostics results from the external laboratory (continuous) | X | X | X |
| Distance to this laboratory (continuous) | X | X | X |
| Who takes bloods (doctor/another person) | X | X | X |
| **Country characteristics** |  |  |  |
| Health expenditure per capita (continuous) |  |  | X |
| Main financing scheme (government/mandatory social health insurance/voluntary insurance or out-of-pocket) |  |  | X |
| **Analysis of hospitals** |  |  |  |
| **Levels** |  |  |  |
| Workplace (hospital) | X | X | X |
| Country of work |  | X | X |
| **Hospital characteristics** |  |  |  |
| Sector of activity (private/public) | X | X | X |
| Level of care (secondary/tertiary hospital), | X | X | X |
| Hospital specialty (general hospital/paediatric or mother and child hospital) | X | X | X |
| Hospital lab turnaround time for routine tests (continuous) | X | X | X |
| Who takes bloods (doctor/another person | X | X | X |
| **Country characteristics** |  |  |  |
| Health expenditure per capita (continuous) |  |  | X |
| Main financing scheme (government/mandatory social health insurance/voluntary insurance or out-of-pocket) |  |  | X |

## Multilevel analysis to identify determinants of the use of POCTs in the clinical scenario

We used a similar approach for the multilevel analyses to identify determinants of the use of each POCT in the clinical scenario except that we used only two models instead of three (Table 5). Model 4 was similar to Model 1, except that it included workplace and clinician characteristics (not only workplace characteristics), as we considered that clinicians’ characteristics are important in the decision to use a diagnostic. We kept workplace and clinician characteristics as a single level, because most workplaces had only one clinician participating in the survey. To use an additional level for clinician characteristics, we would have needed several clinicians for most workplaces· Model 5 was similar to Model 2. We did not have a third model in which we would have assessed the effect of country characteristics on the outcome, because the two measured country characteristics we used in Model 3 were not considered relevant to the decision made by a clinician to use a POCT.

The workplace characteristics were the same as in Models 1, 2 and 3. The clinician characteristics in primary care were: years of clinical practice (continuous) and average consultation time (continuous). In hospitals we added clinical expertise (general paediatrician/trainee/specialist paediatrician) to these two characteristics (Table 5).

| **Table 5· The two models for the multilevel analysis on the use of POCTs in the clinical scenario** | | |
| --- | --- | --- |
|  | **Model 4** | **Model 5** |
| **Purpose** | Base model to be used as a comparator when assessing the effect of adding country as a whole | To assess the effect of adding country as a whole to Model 4. This allows assessment of the effect of workplace and clinician characteristics, and of country as a whole on the use of POCTs |
| **Measure of assessment** | - | 1· Change in area under the curve  2· Median odds ratios  3· Odds ratios |
| **Analysis of primary care practices** |  |  |
| **Levels** |  |  |
| Workplace (Primary care practice) | X | X |
| Country of work |  | X |
| **Clinicians and primary care practice characteristics** |  |  |
| Years of clinical practice (continuous) | X | X |
| Average consultation time (continuous) | X | X |
| Sector of activity (private/public) | X | X |
| Practice size (solo/ group practice) | X | X |
| Turnaround time for diagnostics results from the external laboratory (continuous) | X | X |
| Distance to this laboratory (continuous) | X | X |
| Who takes bloods (doctor/another person) | X | X |
| **Analysis of hospitals** |  |  |
| **Levels** |  |  |
| Workplace (hospital) | X | X |
| Country of work |  | X |
| **Clinicians and hospital characteristics** |  |  |
| Years of clinical practice (continuous) | X | X |
| Clinical expertise (general paediatrician/trainee/specialist paediatrician) | X | X |
| Average consultation time (continuous) | X | X |
| Sector of activity (private/public) | X | X |
| Level of care (secondary/tertiary hospital) | X | X |
| Hospital specialty (general hospital/paediatric or mother and child hospital) | X | X |
| Hospital lab turnaround time for routine tests (continuous) | X | X |
| Who takes bloods (doctor/another person | X | X |

# **References**

1. Merlo J, Wagner P, Ghith N, Leckie G. An Original Stepwise Multilevel Logistic Regression Analysis of Discriminatory Accuracy: The Case of Neighbourhoods and Health· PLoS One· 2016;11(4):e0153778.
