# Supplementary material for: Availability and use of rapid diagnostic tests for the management of acute childhood infections in Europe: A cross-sectional survey of paediatricians
Source: PLoS One. 2022 Dec 20;17(12):e0275336. doi: 10.1371/journal.pone.0275336 (PMC9767335; doi:10.1371/journal.pone.0275336)
Supplement: S4 Supplementary materials — (DOCX) [file pone.0275336.s005.docx]

# **S4 Supplementary Materials: directed acyclic graphs for the inclusion of explanatory variable in the regression models**

We used the directed acyclic graphs approach to choose which covariates to include in the multilevel analyses to minimize the magnitude of the bias in the estimate produced.

Boxes’ colour code:

- Green box: main explanatory variable
- Blue box: outcome
- Grey boxes: measured explanatory variables
- White boxes: unmeasured explanatory variables

**Availability of POCTs in primary care**

*except for UD, GAS, RSV, and influenza POCTS

**Availability of POCTs in hospitals**

*except for UD, GAS, RSV, and influenza POCTS

**Use of POCTs in an infant with undifferentiated fever in primary care**

*except for UD, RSV, and influenza POCTS

**Use of POCTs in an infant with undifferentiated fever in hospitals**

*except for UD, RSV, and influenza POCTS
