# Supplementary material for: Availability and use of rapid diagnostic tests for the management of acute childhood infections in Europe: A cross-sectional survey of paediatricians
Source: PLoS One. 2022 Dec 20;17(12):e0275336. doi: 10.1371/journal.pone.0275336 (PMC9767335; doi:10.1371/journal.pone.0275336)
Supplement: S5 Supplementary materials — (DOCX) [file pone.0275336.s006.docx]

# **S5 Supplementary Materials: flow diagram of included observations per analysis**

Only questionnaires that provided data on the outcomes and potential explanatory variables were included in the analysis. The number of questionnaires that were excluded and the reasons for exclusion are summarised below:
