# Supplementary material for: Availability and use of rapid diagnostic tests for the management of acute childhood infections in Europe: A cross-sectional survey of paediatricians
Source: PLoS One. 2022 Dec 20;17(12):e0275336. doi: 10.1371/journal.pone.0275336 (PMC9767335; doi:10.1371/journal.pone.0275336)
Supplement: S6 Supplementary materials — (DOCX) [file pone.0275336.s007.docx]

# **S6 Supplementary Materials: availability of POCTs per country**

**Primary care**

| **Country** | **Urine Dipstick** | | |
| --- | --- | --- | --- |
|  | Number of primary care practices | Proportion with POCT available | [95% Conf· Interval] |
|  |  |  |  |
| Austria | 73 | 0·986 | 0·926 1·000 |
| Belgium | 8 | 0·875 | 0·473 0·997 |
| Croatia | 10 | 0·700 | 0·348 0·933 |
| Cyprus | 22 | 0·727 | 0·498 0·893 |
| Czech Rep· | 15 | 0·867 | 0·595 0·983 |
| Finland | 11 | 1·000 | 0·715 1·000 |
| France | 156 | 0·929 | 0·877 0·964 |
| Germany | 98 | 0·990 | 0·944 1·000 |
| Greece | 63 | 0·635 | 0·504 0·753 |
| Hungary | 76 | 1·000 | 0·953 1·000 |
| Israel | 56 | 0·964 | 0·877 0·996 |
| Italy | 144 | 0·944 | 0·893 0·976 |
| Latvia | 6 | 0·667 | 0·223 0·957 |
| Lithuania | 6 | 0·667 | 0·223 0·957 |
| Poland | 35 | 0·514 | 0·340 0·686 |
| Slovenia | 88 | 0·852 | 0·761 0·919 |
| Spain | 173 | 0·988 | 0·959 0·999 |
| Switzerland | 51 | 0·980 | 0·896 1·000 |
| Ukraine | 63 | 0·683 | 0·553 0·794 |
| Total | 154 | 0·900 | 0·882 0·917 |

| **Country** | **GAS** | | |
| --- | --- | --- | --- |
|  | Number of primary care practices | Proportion with POCT available | [95% Conf· Interval] |
|  |  |  |  |
| Austria | 73 | 0·904 | 0·812 0·961 |
| Belgium | 8 | 0·625 | 0·245 0·915 |
| Croatia | 10 | 0·5 | 0·187 0·813 |
| Cyprus | 22 | 0·727 | 0·498 0·893 |
| Czech Rep· | 15 | 1 | 0·782 1·000 |
| Finland | 11 | 0·818 | 0·482 0·977 |
| France | 156 | 0·962 | 0·918 0·986 |
| Germany | 98 | 0·939 | 0·871 0·977 |
| Greece | 63 | 0·667 | 0·537 0·780 |
| Hungary | 76 | 0·5 | 0·383 0·617 |
| Israel | 56 | 0·911 | 0·804 0·970 |
| Italy | 144 | 0·951 | 0·902 0·980 |
| Latvia | 6 | 0·833 | 0·359 0·996 |
| Lithuania | 6 | 0·833 | 0·359 0·996 |
| Poland | 35 | 0·2 | 0·084 0·369 |
| Slovenia | 88 | 0·989 | 0·938 1·000 |
| Spain | 173 | 0·908 | 0·854 0·946 |
| Switzerland | 51 | 1 | 0·930 1·000* |
| Ukraine | 63 | 0·587 | 0·456 0·710 |
| Total | 1154 | 0·845 | 0·823 0·865 |

| **Country** | **RSV** | | |
| --- | --- | --- | --- |
|  | Number of primary care practices | Proportion with POCT available | [95% Conf· Interval] |
|  |  |  |  |
| Austria | 73 | 0·151 | 0·078 0·254 |
| Belgium | 8 | 0·375 | 0·085 0·755 |
| Croatia | 10 | 0·1 | 0·003 0·445 |
| Cyprus | 22 | 0·136 | 0·029 0·349 |
| Czech Rep· | 15 | 0·2 | 0·043 0·481 |
| Finland | 11 | 0·818 | 0·482 0·977 |
| France | 156 | 0·237 | 0·173 0·312 |
| Germany | 98 | 0·122 | 0·065 0·204 |
| Greece | 63 | 0·159 | 0·079 0·273 |
| Hungary | 76 | 0·066 | 0·022 0·147 |
| Israel | 56 | 0·018 | 0·000 0·096 |
| Italy | 144 | 0·076 | 0·039 0·133 |
| Latvia | 6 | 0·167 | 0·004 0·641 |
| Lithuania | 6 | 0 | 0·000 0·459 |
| Poland | 35 | 0·029 | 0·001 0·149 |
| Slovenia | 88 | 0·159 | 0·090 0·252 |
| Spain | 173 | 0·04 | 0·016 0·082 |
| Switzerland | 51 | 0·529 | 0·385 0·671 |
| Ukraine | 63 | 0·127 | 0·056 0·235 |
| Total | 1154 | 0·142 | 0·122 0·164 |

| **Country** | **Influenza** | | |
| --- | --- | --- | --- |
|  | Number of primary care practices | Proportion with POCT available | [95% Conf· Interval] |
|  |  |  |  |
| Austria | 73 | 0·301 | 0·199 0·420 |
| Belgium | 8 | 0·375 | 0·085 0·755 |
| Croatia | 10 | 0·1 | 0·003 0·445 |
| Cyprus | 22 | 0·455 | 0·244 0·678 |
| Czech Rep· | 15 | 0·133 | 0·017 0·405 |
| Finland | 11 | 0·909 | 0·587 0·998 |
| France | 156 | 0·397 | 0·320 0·479 |
| Germany | 98 | 0·286 | 0·199 0·386 |
| Greece | 63 | 0·333 | 0·220 0·463 |
| Hungary | 76 | 0·066 | 0·022 0·147 |
| Israel | 56 | 0·071 | 0·020 0·173 |
| Italy | 144 | 0·167 | 0·110 0·238 |
| Latvia | 6 | 0·333 | 0·043 0·777 |
| Lithuania | 6 | 0·5 | 0·118 0·882 |
| Poland | 35 | 0·114 | 0·032· 0·267 |
| Slovenia | 88 | 0·284 | 0·193 0·390 |
| Spain | 173 | 0·092 | 0·054 0·146 |
| Switzerland | 51 | 0·549 | 0·403 0·689 |
| Ukraine | 63 | 0·603 | 0·472 0·724 |
| Total | 1154 | 0·267 | 0·242 0·293 |

| **Country** | **CRP** | | |
| --- | --- | --- | --- |
|  | Number of primary care practices | Proportion with POCT available | [95% Conf· Interval] |
|  |  |  |  |
| Austria | 73 | 0·945 | 0·866 0·985 |
| Belgium | 8 | 0·375 | 0·085 0·755 |
| Croatia | 10 | 0·700 | 0·348 0·933 |
| Cyprus | 22 | 0·500 | 0·282 0·718 |
| Czech Rep· | 15 | 1·000 | 0·782 1·000 |
| Finland | 11 | 0·818 | 0·482 0·977 |
| France | 156 | 0·468 | 0·388 0·549 |
| Germany | 98 | 0·765 | 0·669 0·845 |
| Greece | 63 | 0·222 | 0·127 0·345 |
| Hungary | 76 | 0·737 | 0·623 0·831 |
| Israel | 56 | 0·286 | 0·173 0·422 |
| Italy | 144 | 0·444 | 0·362 0·529 |
| Latvia | 6 | 0·333 | 0·043 0·777 |
| Lithuania | 6 | 0·833 | 0·359 0·996 |
| Poland | 35 | 0·200 | 0·084 0·369 |
| Slovenia | 88 | 0·784 | 0·684 0·865 |
| Spain | 173 | 0·139 | 0·091 0·199 |
| Switzerland | 51 | 0·941 | 0·838 0·988 |
| Ukraine | 63 | 0·413 | 0·290 0·544 |
| Total | 1154 | 0·514 | 0·485 0·543 |

| **Country** | **Procalcitonin** | | |
| --- | --- | --- | --- |
|  | Number of primary care practices | Proportion with POCT available | [95% Conf· Interval] |
|  |  |  |  |
| Austria | 73 | 0·068 | 0·023 0·153 |
| Belgium | 8 | 0·125 | 0·003 0·527 |
| Croatia | 10 | 0·1 | 0·003 0·445 |
| Cyprus | 22 | 0·136 | 0·029 0·349 |
| Czech Rep· | 15 | 0·267 | 0·078 0·551 |
| Finland | 11 | 0·182 | 0·023 0·518 |
| France | 156 | 0·006 | 0·000 0·035 |
| Germany | 98 | 0·01 | 0·000 0·056 |
| Greece | 63 | 0·032 | 0·004 0·110 |
| Hungary | 76 | 0·013 | 0·000 0·071 |
| Israel | 56 | 0·071 | 0·020 0·173 |
| Italy | 144 | 0·049 | 0·020 0·098 |
| Latvia | 6 | 0·333 | 0·043 0·777 |
| Lithuania | 6 | 0 | 0·000 0·459 |
| Poland | 35 | 0·086 | 0·018 0·231 |
| Slovenia | 88 | 0·057 | 0·019 0·128 |
| Spain | 173 | 0·087 | 0·049 0·139 |
| Switzerland | 51 | 0·137 | 0·057 0·263 |
| Ukraine | 63 | 0·222 | 0·127 0·345 |
| Total | 1154 | 0·068 | 0·054 0·084 |

| **Country** | **Full blood count** | | |
| --- | --- | --- | --- |
|  | Number of primary care practices | Proportion with POCT available | [95% Conf· Interval] |
|  |  |  |  |
| Austria | 73 | 0·89 | 0·795 0·951 |
| Belgium | 8 | 0·25 | 0·032 0·651 |
| Croatia | 10 | 0·6 | 0·262 0·878 |
| Cyprus | 22 | 0·318 | 0·139 0·549 |
| Czech Rep· | 15 | 0·133 | 0·017 0·405 |
| Finland | 11 | 0·818 | 0·482 0·977 |
| France | 156 | 0·026 | 0·007 0·064 |
| Germany | 98 | 0·469 | 0·368 0·573 |
| Greece | 63 | 0·19 | 0·102 0·309 |
| Hungary | 76 | 0·158 | 0·084 0·260 |
| Israel | 56 | 0·286 | 0·173 0·422 |
| Italy | 144 | 0·146 | 0·093 0·214 |
| Latvia | 6 | 0·333 | 0·043 0·777 |
| Lithuania | 6 | 0·833 | 0·359 0·996 |
| Poland | 35 | 0·571 | 0·394 0·737 |
| Slovenia | 88 | 0·727 | 0·622 0·817 |
| Spain | 173 | 0·191 | 0·135 0·257 |
| Switzerland | 51 | 0·804 | 0·669 0·902 |
| Ukraine | 63 | 0·667 | 0·537 0·780 |
| Total | 1154 | 0·354 | 0·327 0·383 |

| **Country** | **Blood gas analysis (with or without lactate)** | | |
| --- | --- | --- | --- |
|  | Number of primary care practices | Proportion with POCT available | [95% Conf· Interval] |
|  |  |  |  |
| Austria | 73 | 0·055 | 0·015 0·134 |
| Belgium | 8 | 0·125 | 0·003 0·527 |
| Croatia | 10 | 0·1 | 0·003 0·445 |
| Cyprus | 22 | 0·045 | 0·001 0·228 |
| Czech Rep· | 15 | 0·2 | 0·043 0·481 |
| Finland | 11 | 0·182 | 0·023 0·518 |
| France | 156 | 0·071 | 0·036 0·123 |
| Germany | 98 | 0·051 | 0·017 0·115 |
| Greece | 63 | 0·079 | 0·026 0·176 |
| Hungary | 76 | 0·066 | 0·022 0·147 |
| Israel | 56 | 0·036 | 0·004 0·123 |
| Italy | 144 | 0·056 | 0·024 0·107 |
| Latvia | 6 | 0·333 | 0·043 0·777 |
| Lithuania | 6 | 0 | 0·000 0·459 |
| Poland | 35 | 0·429 | 0·263 0·606 |
| Slovenia | 88 | 0·068 | 0·025 0·143 |
| Spain | 173 | 0·052 | 0·024 0·096 |
| Switzerland | 51 | 0·098 | 0·033 0·214 |
| Ukraine | 63 | 0·175 | 0·091 0·291 |
| Total | 1154 | 0·083 | 0·068 0·101 |

| **Country** | **Lactate** | | |
| --- | --- | --- | --- |
|  | Number of primary care practices | Proportion with POCT available | [95% Conf· Interval] |
|  |  |  |  |
| Austria | 73 | 0·068 | 0·023 0·153 |
| Belgium | 8 | 0·25 | 0·032 0·651 |
| Croatia | 10 | 0 | 0·000 0·308 |
| Cyprus | 22 | 0·045 | 0·001 0·228 |
| Czech Rep· | 15 | 0 | 0·000 0·218 |
| Finland | 11 | 0·091 | 0·002 0·413 |
| France | 156 | 0·045 | 0·018 0·090 |
| Germany | 98 | 0·02 | 0·002 0·072 |
| Greece | 63 | 0·032 | 0·004 0·110 |
| Hungary | 76 | 0·066 | 0·022 0·147 |
| Israel | 56 | 0·107 | 0·040 0·219 |
| Italy | 144 | 0·042 | 0·015 0·088 |
| Latvia | 6 | 0·333 | 0·043 0·777 |
| Lithuania | 6 | 0 | 0·000 0·459 |
| Poland | 35 | 0·114 | 0·032 0·267 |
| Slovenia | 88 | 0·102 | 0·048 0·185 |
| Spain | 173 | 0·029 | 0·009 0·066 |
| Switzerland | 51 | 0·078 | 0·022 0·189 |
| Ukraine | 63 | 0·143 | 0·067 0·254 |
| Total | 1154 | 0·061 | - 1. 0·076 |

**Hospitals**

| **Country** | **Urine dipstick** | | |
| --- | --- | --- | --- |
|  | Number of hospitals | Proportion with POCT available | [95% Conf· Interval] |
| Austria | 30 | 1·000 | 0·884 1·000 |
| Belgium | 40 | 0·875 | 0·732 0·958 |
| Bulgaria | 3 | 0·667 | 0·094 0·992 |
| Croatia | 13 | 0·923 | 0·640 0·998 |
| Cyprus | 3 | 1·000 | 0·292 1·000 |
| Czech Rep· | 1 | 1·000 | 0·025 1·000 |
| Denmark | 3 | 1·000 | 0·292 1·000 |
| Finland | 12 | 1·000 | 0·735 1·000 |
| France | 46 | 0·978 | 0·885 0·999 |
| Germany | 43 | 0·907 | 0·779 0·974 |
| Greece | 29 | 0·862 | 0·683 0·961 |
| Hungary | 12 | 1·000 | 0·735 1·000 |
| Ireland | 4 | 1·000 | 0·398 1·000 |
| Israel | 4 | 1·000 | 0·398 1·000 |
| Italy | 20 | 1·000 | 0·832 1·000 |
| Latvia | 4 | 1·000 | 0·398 1·000 |
| Lithuania | 3 | 0·333 | 0·008 0·906 |
| Malta | 3 | 1·000 | 0·292 1·000 |
| The Netherlands | 27 | 0·667 | 0·460 0·835 |
| Norway | 23 | 1·000 | 0·852 1·000 |
| Poland | 11 | 0·545 | 0·234 0·833 |
| Portugal | 23 | 1·000 | 0·852 1·000 |
| Romania | 6 | 1·000 | 0·541 1·000 |
| Slovenia | 7 | 0·571 | 0·184 0·901 |
| Spain | 50 | 0·980 | 0·894 0·999 |
| Sweden | 8 | 1·000 | 0·631 1·000 |
| Switzerland | 11 | 1·000 | 0·715 1·000 |
| Ukraine | 27 | 0·741 | 0·537 0·889 |
| United Kingdom | 38 | 1·000 | 0·907 1·000 |
| Total | 504 | 0·915 | 0·887 0·938 |

| **Country** | **GAS** | | |
| --- | --- | --- | --- |
|  | Number of hospitals | Proportion with POCT available | [95% Conf· Interval] |
| Austria | 30 | 0·900 | 0·735 0·979 |
| Belgium | 40 | 0·550 | 0·385 0·707 |
| Bulgaria | 3 | 0·667 | 0·094 0·992 |
| Croatia | 13 | 0·538 | 0·251 0·808 |
| Cyprus | 3 | 0·667 | 0·094 0·992 |
| Czech Rep· | 1 | 1·000 | 0·025 1·000 |
| Denmark | 3 | 0·667 | 0·094 0·992 |
| Finland | 12 | 0·917 | 0·615 0·998 |
| France | 46 | 0·978 | 0·885 0·999 |
| Germany | 43 | 0·651 | 0·491 0·790 |
| Greece | 29 | 0·828 | 0·642 0·942 |
| Hungary | 12 | 0·167 | 0·021 0·484 |
| Ireland | 4 | 0 | 0·000 0·602 |
| Israel | 4 | 0·250 | 0·006 0·806 |
| Italy | 20 | 0·650 | 0·408 0·846 |
| Latvia | 4 | 0·500 | 0·068 0·932 |
| Lithuania | 3 | 0·667 | 0·094 0·992 |
| Malta | 3 | 0·667 | 0·094 0·992 |
| The Netherlands | 27 | 0·074 | 0·009 0·243 |
| Norway | 23 | 0·870 | 0·664 0·972 |
| Poland | 11 | 0·636 | 0·308 0·891 |
| Portugal | 23 | 0·783 | 0·563 0·925 |
| Romania | 6 | 0·667 | 0·223 0·957 |
| Slovenia | 7 | 1·000 | 0·590 1·000 |
| Spain | 50 | 0·900 | 0·782 0·967 |
| Sweden | 8 | 1·000 | 0·631 1·000 |
| Switzerland | 11 | 1·000 | 0·715 1·000 |
| Ukraine | 27 | 0·481 | 0·287 0·681 |
| United Kingdom | 38 | 0·026 | 0·001 0·138 |
| Total | 504 | 0·653 | 0·609 0·694 |

| **Country** | **RSV** | | |
| --- | --- | --- | --- |
|  | Number of hospitals | Proportion with POCT available | [95% Conf· Interval] |
| Austria | 30 | 0·767 | 0·577 0·901 |
| Belgium | 40 | 0·75 | 0·588 0·873 |
| Bulgaria | 3 | 0·333 | 0·008 0·906 |
| Croatia | 13 | 0·615 | 0·316 0·861 |
| Cyprus | 3 | 0 | 0·000 0·708 |
| Czech Rep· | 1 | 1 | 0·025 1·000 |
| Denmark | 3 | 0·667 | 0·094 0·992 |
| Finland | 12 | 0·75 | 0·428 0·945 |
| France | 46 | 0·37 | 0·232 0·525 |
| Germany | 43 | 0·767 | 0·614 0·882 |
| Greece | 29 | 0·517 | 0·325 0·706 |
| Hungary | 12 | 0·417 | 0·152 0·723 |
| Ireland | 4 | 0·25 | 0·006 0·806 |
| Israel | 4 | 0 | 0·000 0·602 |
| Italy | 20 | 0·35 | 0·154 0·592 |
| Latvia | 4 | 0·25 | 0·006 0·806 |
| Lithuania | 3 | 0 | 0·000 0·708 |
| Malta | 3 | 0·333 | 0·008 0·906 |
| The Netherlands | 27 | 0·63 | 0·424 0·806 |
| Norway | 23 | 0·565 | 0·345 0·768 |
| Poland | 11 | 0·818 | 0·482 0·977 |
| Portugal | 23 | 0·522 | 0·306 0·732 |
| Romania | 6 | 0·5 | 0·118 0·882 |
| Slovenia | 7 | 0·571 | 0·184 0·901 |
| Spain | 50 | 0·88 | 0·757 0·955 |
| Sweden | 8 | 0·75 | 0·349 0·968 |
| Switzerland | 11 | 0·818 | 0·482 0·977 |
| Ukraine | 27 | 0·148 | 0·042 0·337 |
| United Kingdom | 38 | 0·289 | 0·154 0·459 |
| Total | 504 | 0·567 | 0·523 0·611 |

| **Country** | **Influenza** | | |
| --- | --- | --- | --- |
|  | Number of hospitals | Proportion with POCT available | [95% Conf· Interval] |
| Austria | 30 | 0·800 | 0·614 0·923 |
| Belgium | 40 | 0·750 | 0·588 0·873 |
| Bulgaria | 3 | 0·667 | 0·094 0·992 |
| Croatia | 13 | 0·538 | 0·251 0·808 |
| Cyprus | 3 | 0·333 | 0·008 0·906 |
| Czech Rep· | 1 | 1·000 | 0·025 1·000 |
| Denmark | 3 | 0·667 | 0·094 0·992 |
| Finland | 12 | 0·750 | 0·428 0·945 |
| France | 46 | 0·652 | 0·498 0·786 |
| Germany | 43 | 0·651 | 0·491 0·790 |
| Greece | 29 | 0·552 | 0·357 0·736 |
| Hungary | 12 | 0·167 | 0·021 0·484 |
| Ireland | 4 | 0·250 | 0·006 0·806 |
| Israel | 4 | 0·000 | 0·000 0·602 |
| Italy | 20 | 0·100 | 0·012 0·317 |
| Latvia | 4 | 0·500 | 0·068 0·932 |
| Lithuania | 3 | 0·333 | 0·008 0·906 |
| Malta | 3 | 0·333 | 0·008 0·906 |
| The Netherlands | 27 | 0·741 | 0·537 0·889 |
| Norway | 23 | 0·522 | 0·306 0·732 |
| Poland | 11 | 0·636 | 0·308 0·891 |
| Portugal | 23 | 0·522 | 0·306 0·732 |
| Romania | 6 | 0·667 | 0·223 0·957 |
| Slovenia | 7 | 0·571 | 0·184 0·901 |
| Spain | 50 | 0·840 | 0·709 0·928 |
| Sweden | 8 | 0·750 | 0·349 0·968 |
| Switzerland | 11 | 0·727 | 0·390 0·940 |
| Ukraine | 27 | 0·519 | 0·319 0·713 |
| United Kingdom | 38 | 0·316 | 0·175 0·487 |
| Total | 504 | 0·595 | 0·551 0·638 |

| **Country** | **CRP** | | |
| --- | --- | --- | --- |
|  | Number of hospitals | Proportion with POCT available | [95% Conf· Interval] |
| Austria | 30 | 0·900 | 0·735 0·979 |
| Belgium | 40 | 0·300 | 0·166 0·465 |
| Bulgaria | 3 | 0·667 | 0·094 0·992 |
| Croatia | 13 | 0·615 | 0·316 0·861 |
| Cyprus | 3 | 0·333 | 0·008 0·906 |
| Czech Rep· | 1 | 1·000 | 0·025 1·000 |
| Denmark | 3 | 0·667 | 0·094 0·992 |
| Finland | 12 | 0·833 | 0·516 0·979 |
| France | 46 | 0·370 | 0·232 0·525 |
| Germany | 43 | 0·349 | 0·210 0·509 |
| Greece | 29 | 0·724 | 0·528 0·873 |
| Hungary | 12 | 0·583 | 0·277 0·848 |
| Ireland | 4 | 0·000 | 0·000 0·602 |
| Israel | 4 | 0·250 | 0·006 0·806 |
| Italy | 20 | 0·350 | 0·154 0·592 |
| Latvia | 4 | 0·250 | 0·006 0·806 |
| Lithuania | 3 | 0·667 | 0·094 0·992 |
| Malta | 3 | 0·333 | 0·008 0·906 |
| The Netherlands | 27 | 0·185 | 0·063 0·381 |
| Norway | 23 | 0·348 | 0·164 0·573 |
| Poland | 11 | 0·545 | 0·234 0·833 |
| Portugal | 23 | 0·522 | 0·306 0·732 |
| Romania | 6 | 0·833 | 0·359 0·996 |
| Slovenia | 7 | 0·143 | 0·004 0·579 |
| Spain | 50 | 0·700 | 0·554 0·821 |
| Sweden | 8 | 1·000 | 0·631 1·000 |
| Switzerland | 11 | 0·455 | 0·167 0·766 |
| Ukraine | 27 | 0·519 | 0·319 0·713 |
| United Kingdom | 38 | 0·053 | 0·006 0·177 |
| Total | 504 | 0·468 | 0·424 0·513 |

| **Country** | **Procalcitonin** | | |
| --- | --- | --- | --- |
|  | Number of hospitals | Proportion with POCT available | [95% Conf· Interval] |
| Austria | 30 | 0·367 | 0·199 0·561 |
| Belgium | 40 | 0·075 | 0·016 0·204 |
| Bulgaria | 3 | 0 | 0·000 0·708 |
| Croatia | 13 | 0·385 | 0·139 0·684 |
| Cyprus | 3 | 0·333 | 0·008 0·906 |
| Czech Rep· | 1 | 1 | 0·025 1·000 |
| Denmark | 3 | 0 | 0·000 0·708 |
| Finland | 12 | 0·167 | 0·021 0·484 |
| France | 46 | 0·174 | 0·078 0·314 |
| Germany | 43 | 0·256 | 0·135 0·412 |
| Greece | 29 | 0·241 | 0·103 0·435 |
| Hungary | 12 | 0·417 | 0·152 0·723 |
| Ireland | 4 | 0 | 0·000 0·602 |
| Israel | 4 | 0 | 0·000 0·602 |
| Italy | 20 | 0·2 | 0·057 0·437 |
| Latvia | 4 | 0·25 | 0·006 0·806 |
| Lithuania | 3 | 0·333 | 0·008 0·906 |
| Malta | 3 | 0·333 | 0·008 0·906 |
| The Netherlands | 27 | 0 | 0·000 0·128 |
| Norway | 23 | 0·13 | 0·028 0·336 |
| Poland | 11 | 0·545 | 0·234 0·833 |
| Portugal | 23 | 0·174 | 0·050 0·388 |
| Romania | 6 | 0·333 | 0·043 0·777 |
| Slovenia | 7 | 0 | 0·000 0·410 |
| Spain | 50 | 0·62 | 0·472 0·753 |
| Sweden | 8 | 0·25 | 0·032 0·651 |
| Switzerland | 11 | 0·091 | 0·002 0·413 |
| Ukraine | 27 | 0·407 | 0·224 0·612 |
| United Kingdom | 38 | 0·026 | 0·001 0·138 |
| Total | 504 | 0·242 | 0·205 0·282 |

| **Country** | **Full blood count** | | |
| --- | --- | --- | --- |
|  | Number of hospitals | Proportion with POCT available | [95% Conf· Interval] |
| Austria | 30 | 0·767 | 0·577 0·901 |
| Belgium | 40 | 0·25 | 0·127 0·412 |
| Bulgaria | 3 | 1 | 0·292 1·000 |
| Croatia | 13 | 0·692 | 0·386 0·909 |
| Cyprus | 3 | 0·667 | 0·094 0·992 |
| Czech Rep· | 1 | 1 | 0·025 1·000 |
| Denmark | 3 | 0 | 0·000 0·708 |
| Finland | 12 | 0·75 | 0·428 0·945 |
| France | 46 | 0·152 | 0·063 0·289 |
| Germany | 43 | 0·395 | 0·250 0·556 |
| Greece | 29 | 0·69 | 0·492 0·847 |
| Hungary | 12 | 0·417 | 0·152 0·723 |
| Ireland | 4 | 0·25 | 0·006 0·806 |
| Israel | 4 | 0·25 | 0·006 0·806 |
| Italy | 20 | 0·25 | 0·087 0·491 |
| Latvia | 4 | 0·25 | 0·006 0·806 |
| Lithuania | 3 | 0·667 | 0·094 0·992 |
| Malta | 3 | 0·333 | 0·008 0·906 |
| The Netherlands | 27 | 0·111 | 0·024 0·292 |
| Norway | 23 | 0·304 | 0·132 0·529 |
| Poland | 11 | 0·545 | 0·234 0·833 |
| Portugal | 23 | 0·522 | 0·306 0·732 |
| Romania | 6 | 1 | 0·541 1·000 |
| Slovenia | 7 | 0 | 0·000 0·410 |
| Spain | 50 | 0·7 | 0·554 0·821 |
| Sweden | 8 | 0·375 | 0·085 0·755 |
| Switzerland | 11 | 0·091 | 0·002 0·413 |
| Ukraine | 27 | 0·778 | 0·577 0·914 |
| United Kingdom | 38 | 0·105 | 0·029 0·248 |
| Total | 504 | 0·427 | 0·383 0·471 |

| **Country** | **Blood gas (with or without lactate)** | | |
| --- | --- | --- | --- |
|  | Number of hospitals | Proportion with POCT available | [95% Conf· Interval] |
| Austria | 30 | 0·967 | 0·828 0·999 |
| Belgium | 40 | 0·775 | 0·615 0·892 |
| Bulgaria | 3 | 1 | 0·292 1·000 |
| Croatia | 13 | 0·769 | 0·462 0·950 |
| Cyprus | 3 | 1 | 0·292 1·000 |
| Czech Rep· | 1 | 1 | 0·025 1·000 |
| Denmark | 3 | 0·333 | 0·008 0·906 |
| Finland | 12 | 0·75 | 0·428 0·945 |
| France | 46 | 0·565 | 0·411 0·711 |
| Germany | 43 | 0·977 | 0·877 0·999 |
| Greece | 29 | 0·897 | 0·726 0·978 |
| Hungary | 12 | 0·917 | 0·615 0·998 |
| Ireland | 4 | 1 | 0·398 1·000 |
| Israel | 4 | 0·75 | 0·194 0·994 |
| Italy | 20 | 0·95 | 0·751 0·999 |
| Latvia | 4 | 0·75 | 0·194 0·994 |
| Lithuania | 3 | 1 | 0·292 1·000 |
| Malta | 3 | 1 | 0·292 1·000 |
| The Netherlands | 27 | 0·667 | 0·460 0·835 |
| Norway | 23 | 0·957 | 0·781 0·999 |
| Poland | 11 | 0·636 | 0·308 0·891 |
| Portugal | 23 | 0·826 | 0·612 0·950 |
| Romania | 6 | 0·833 | 0·359 0·996 |
| Slovenia | 7 | 0·857 | 0·421 0·996 |
| Spain | 50 | 0·88 | 0·757 0·955 |
| Sweden | 8 | 1 | 0·631 1·000 |
| Switzerland | 11 | 1 | 0·715 1·000 |
| Ukraine | 27 | 0·481 | 0·287 0·681 |
| United Kingdom | 38 | 1 | 0·907 1·000 |
| Total | 504 | 0·829 | 0·794 0·861 |

| **Country** | **Lactate** | | |
| --- | --- | --- | --- |
|  | Number of hospitals | Proportion with POCT available | [95% Conf· Interval] |
| Austria | 30 | 0·433 | 0·255 0·626 |
| Belgium | 40 | 0·35 | 0·206 0·517 |
| Bulgaria | 3 | 0·333 | 0·008 0·906 |
| Croatia | 13 | 0·538 | 0·251 0·808 |
| Cyprus | 3 | 0·667 | 0·094 0·992 |
| Czech Rep· | 1 | 1 | 0·025 1·000 |
| Denmark | 3 | 0 | 0·000 0·708 |
| Finland | 12 | 0·25 | 0·055 0·572 |
| France | 46 | 0·457 | 0·309 0·610 |
| Germany | 43 | 0·605 | 0·444 0·750 |
| Greece | 29 | 0·241 | 0·103 0·435 |
| Hungary | 12 | 0·5 | 0·211 0·789 |
| Ireland | 4 | 0·5 | 0·068 0·932 |
| Israel | 4 | 0·5 | 0·068 0·932 |
| Italy | 20 | 0·45 | 0·231 0·685 |
| Latvia | 4 | 0·5 | 0·068 0·932 |
| Lithuania | 3 | 0·667 | 0·094 0·992 |
| Malta | 3 | 0·333 | 0·008 0·906 |
| The Netherlands | 27 | 0·222 | 0·086 0·423 |
| Norway | 23 | 0·478 | 0·268 0·694 |
| Poland | 11 | 0·455 | 0·167 0·766 |
| Portugal | 23 | 0·217 | 0·075 0·437 |
| Romania | 6 | 0·167 | 0·004 0·641 |
| Slovenia | 7 | 0 | 0·000 0·410 |
| Spain | 50 | 0·62 | 0·472 0·753 |
| Sweden | 8 | 0·375 | 0·085 0·755 |
| Switzerland | 11 | 0·364 | 0·109 0·692 |
| Ukraine | 27 | 0·296 | 0·138 0·502 |
| United Kingdom | 38 | 0·447 | 0·286 0·617 |
| Total | 504 | 0·417 | 0·373 0·461 |
