# Supplementary material for: Availability and use of rapid diagnostic tests for the management of acute childhood infections in Europe: A cross-sectional survey of paediatricians
Source: PLoS One. 2022 Dec 20;17(12):e0275336. doi: 10.1371/journal.pone.0275336 (PMC9767335; doi:10.1371/journal.pone.0275336)
Supplement: S7 Supplementary materials — (DOCX) [file pone.0275336.s008.docx]

# **S7 Supplementary Materials: use of POCTs per country**

# **Primary care**

| **Country** |  | **Urine dipstick** | | |
| --- | --- | --- | --- | --- |
|  |  | I would not use the test | I would use only the POCT version | Total |
|  |  |  |  |  |
| Austria | Proportion | 0·0102 | 0·9898 | 1 |
|  | (95%CI) | [·0014,·0701] | [·9299,·9986] |  |
|  | Number of participants | 1 | 62 | 63 |
|  |  |  |  |  |
| Belgium | Proportion | 0·1677 | 0·8323 | 1 |
|  | (95%CI) | [·0232,·6312] | [·3688,·9768] |  |
|  | Number of participants | 1 | 6 | 7 |
|  |  |  |  |  |
| Croatia | Proportion | 0·0959 | 0·9041 | 1 |
|  | (95%CI) | [·0129,·4624] | [·5376,·9871] |  |
|  | Number of participants | 1 | 8 | 9 |
|  |  |  |  |  |
| Cyprus | Proportion | 0·08 | 0·92 | 1 |
|  | (95%CI) | [·0113,·3989] | [·6011,·9887] |  |
|  | Number of participants | 1 | 16 | 17 |
|  |  |  |  |  |
| Czech Re | Proportion | 0·1845 | 0·8155 | 1 |
|  | (95%CI) | [·0505,·4906] | [·5094,·9495] |  |
|  | Number of participants | 3 | 9 | 12 |
|  |  |  |  |  |
| Finland | Proportion | 0 | 1 | 1 |
|  | (95%CI) |  |  |  |
|  | Number of participants | 0 | 10 | 10 |
|  |  |  |  |  |
| France | Proportion | 0·0712 | 0·9288 | 1 |
|  | (95%CI) | [·0317,·1521] | [·8479,·9683] |  |
|  | Number of participants | 6 | 64 | 70 |
|  |  |  |  |  |
| Germany | Proportion | 0 | 1 | 1 |
|  | (95%CI) |  |  |  |
|  | Number of participants | 0 | 59 | 59 |
|  |  |  |  |  |
| Greece | Proportion | 0·2755 | 0·7245 | 1 |
|  | (95%CI) | [·1653,·422] | [·578,·8347] |  |
|  | Number of participants | 13 | 34 | 47 |
|  |  |  |  |  |
| Hungary | Proportion | 0·0831 | 0·9169 | 1 |
|  | (95%CI) | [·0269,·2292] | [·7708,·9731] |  |
|  | Number of participants | 3 | 58 | 61 |
|  |  |  |  |  |
| Israel | Proportion | 0 | 1 | 1 |
|  | (95%CI) |  |  |  |
|  | Number of participants | 0 | 18 | 18 |
|  |  |  |  |  |
| Italy | Proportion | 0·0294 | 0·9706 | 1 |
|  | (95%CI) | [·0091,·0912] | [·9088,·9909] |  |
|  | Number of participants | 3 | 108 | 111 |
|  |  |  |  |  |
| Latvia | Proportion | 0 | 1 | 1 |
|  | (95%CI) |  |  |  |
|  | Number of participants | 0 | 3 | 3 |
|  |  |  |  |  |
| Lithuania | Proportion | 0·8577 | 0·1423 | 1 |
|  | (95%CI) | [·3519,·9853] | [·0147,·6481] |  |
|  | Number of participants | 3 | 1 | 4 |
|  |  |  |  |  |
| Poland | Proportion | 0·4163 | 0·5837 | 1 |
|  | (95%CI) | [·2067,·6613] | [·3387,·7933] |  |
|  | Number of participants | 9 | 25 | 34 |
|  |  |  |  |  |
| Slovenia | Proportion | 0·2316 | 0·7684 | 1 |
|  | (95%CI) | [·1389,·3601] | [·6399,·8611] |  |
|  | Number of participants | 15 | 53 | 68 |
|  |  |  |  |  |
| Spain | Proportion | 0·0842 | 0·9158 | 1 |
|  | (95%CI) | [·0405,·1669] | [·8331,·9595] |  |
|  | Number of participants | 8 | 118 | 126 |
|  |  |  |  |  |
| Switzerland | Proportion | 0·0218 | 0·9782 | 1 |
|  | (95%CI) | [·003,·1415] | [·8585,·997] |  |
|  | Number of participants | 1 | 31 | 32 |
|  |  |  |  |  |
| Ukraine | Proportion | 0·1534 | 0·8466 | 1 |
|  | (95%CI) | [·0662,·3164] | [·6836,·9338] |  |
|  | Number of participants | 8 | 36 | 44 |
|  |  |  |  |  |
| Total | Proportion | 0·104 | 0·896 | 1 |
|  | (95%CI) | [·0813,·1322] | [·8678,·9187] |  |
|  | Number of participants | 76 | 719 | 795 |

·

| **Country** |  | **RSV** | | | | |
| --- | --- | --- | --- | --- | --- | --- |
|  |  | I would not use the test | I would use both POCT and lab versions of the test | I would use only the lab version of the test | I would use only the POCT version | Total |
|  |  |  |  |  |  |  |
| Austria | Proportion | 0·4563 | 0·0205 | 0·1133 | 0·4098 | 1 |
|  | (95%CI) | [·317,·6029] | [·005,·0797] | [·0339,·3179] | [·2815,·5517] |  |
|  | Number of participants | 29 | 2 | 4 | 28 | 63 |
|  |  |  |  |  |  |  |
| Belgium | Proportion | 0·1994 | 0·1677 | 0·0997 | 0·5332 | 1 |
|  | (95%CI) | [·0452,·567] | [·0232,·6312] | [·0129,·4836] | [·1966,·8421] |  |
|  | Number of participants | 2 | 1 | 1 | 3 | 7 |
|  |  |  |  |  |  |  |
| Croatia | Proportion | 0·5068 | 0 | 0·0959 | 0·3973 | 1 |
|  | (95%CI) | [·2082,·8007] |  | [·0129,·4624] | [·1364,·7334] |  |
|  | Number of participants | 5 | 0 | 1 | 3 | 9 |
|  |  |  |  |  |  |  |
| Cyprus | Proportion | 0·5183 | 0 | 0·36 | 0·1217 | 1 |
|  | (95%CI) | [·2811,·7476] |  | [·1635,·6181] | [·028,·3997] |  |
|  | Number of participants | 9 | 0 | 6 | 2 | 17 |
|  |  |  |  |  |  |  |
| Czech Re· | Proportion | 0·6319 | 0 | 0·0531 | 0·315 | 1 |
|  | (95%CI) | [·2179,·9136] |  | [·0067,·317] | [·0556,·7821] |  |
|  | Number of participants | 10 | 0 | 1 | 1 | 12 |
|  |  |  |  |  |  |  |
| Finland | Proportion | 0·3772 | 0 | 0 | 0·6228 | 1 |
|  | (95%CI) | [·1215,·7262] |  |  | [·2738,·8785] |  |
|  | Number of participants | 5 | 0 | 0 | 5 | 10 |
|  |  |  |  |  |  |  |
| France | Proportion | 0·5121 | 0 | 0·0474 | 0·4404 | 1 |
|  | (95%CI) | [·3838,·6388] |  | [·0106,·1876] | [·3152,·5737] |  |
|  | Number of participants | 42 | 0 | 2 | 26 | 70 |
|  |  |  |  |  |  |  |
| Germany | Proportion | 0·5219 | 0·0764 | 0·0278 | 0·374 | 1 |
|  | (95%CI) | [·3794,·6609] | [·0288,·1876] | [·0069,·1056] | [·2398,·5307] |  |
|  | Number of participants | 34 | 4 | 2 | 19 | 59 |
|  |  |  |  |  |  |  |
| Greece | Proportion | 0·3792 | 0·0766 | 0·0533 | 0·4909 | 1 |
|  | (95%CI) | [·2512,·5264] | [·0242,·2173] | [·0127,·1976] | [·3501,·6331] |  |
|  | Number of participants | 18 | 3 | 2 | 24 | 47 |
|  |  |  |  |  |  |  |
| Hungary | Proportion | 0·3548 | 0·045 | 0·0285 | 0·5718 | 1 |
|  | (95%CI) | [·2171,·5217] | [·0134,·1407] | [·007,·1084] | [·4154,·7149] |  |
|  | Number of participants | 18 | 3 | 2 | 38 | 61 |
|  |  |  |  |  |  |  |
| Israel | Proportion | 0·5549 | 0 | 0 | 0·4451 | 1 |
|  | (95%CI) | [·3274,·7614] |  |  | [·2386,·6726] |  |
|  | Number of participants | 10 | 0 | 0 | 8 | 18 |
|  |  |  |  |  |  |  |
| Italy | Proportion | 0·5515 | 0·0184 | 0·0301 | 0·4 | 1 |
|  | (95%CI) | [·4345,·6632] | [·0059,·0564] | [·0106,·0819] | [·2915,·5193] |  |
|  | Number of participants | 59 | 3 | 4 | 45 | 111 |
|  |  |  |  |  |  |  |
| Latvia | Proportion | 1 | 0 | 0 | 0 | 1 |
|  | (95%CI) |  |  |  |  |  |
|  | Number of participants | 3 | 0 | 0 | 0 | 3 |
|  |  |  |  |  |  |  |
| Lithuania | Proportion | 1 | 0 | 0 | 0 | 1 |
|  | (95%CI) |  |  |  |  |  |
|  | Number of participants | 4 | 0 | 0 | 0 | 4 |
|  |  |  |  |  |  |  |
| Poland | Proportion | 0·1009 | 0·0519 | 0 | 0·8472 | 1 |
|  | (95%CI) | [·022,·3591] | [·0094,·2395] |  | [·6082,·9519] |  |
|  | Number of participants | 4 | 2 | 0 | 28 | 34 |
|  |  |  |  |  |  |  |
| Slovenia | Proportion | 0·5084 | 0·0562 | 0·1208 | 0·3146 | 1 |
|  | (95%CI) | [·3797,·636] | [·0165,·1748] | [·0532,·2513] | [·2099,·4424] |  |
|  | Number of participants | 35 | 3 | 6 | 24 | 68 |
|  |  |  |  |  |  |  |
| Spain | Proportion | 0·2989 | 0·0297 | 0·0226 | 0·6487 | 1 |
|  | (95%CI) | [·2219,·3894] | [·0104,·0819] | [·0048,·1001] | [·5549,·7323] |  |
|  | Number of participants | 42 | 4 | 2 | 78 | 126 |
|  |  |  |  |  |  |  |
| Switzerland | Proportion | 0·6674 | 0·0218 | 0·1561 | 0·1547 | 1 |
|  | (95%CI) | [·4653,·8223] | [·003,·1415] | [·0556,·3678] | [·0567,·3578] |  |
|  | Number of participants | 23 | 1 | 4 | 4 | 32 |
|  |  |  |  |  |  |  |
| Ukraine | Proportion | 0·3226 | 0·0678 | 0 | 0·6096 | 1 |
|  | (95%CI) | [·1824,·5041] | [·027,·16] |  | [·4344,·7605] |  |
|  | Number of participants | 13 | 5 | 0 | 26 | 44 |
|  |  |  |  |  |  |  |
| Total | Proportion | 0·4412 | 0·0355 | 0·0557 | 0·4675 | 1 |
|  | (95%CI) | [·402,·4812] | [·0243,·0516] | [·0386,·0797] | [·4274,·5081] |  |
|  | Number of participants | 365 | 31 | 37 | 362 | 795 |

| **Country** |  | **Influenza** | | | | |
| --- | --- | --- | --- | --- | --- | --- |
|  |  | I would not use the test | I would use both POCT and lab versions of the test | I would use only the lab version of the test | I would use only the POCT version | Total |
|  |  |  |  |  |  |  |
| Austria | Proportion | 0·4948 | 0·0102 | 0·027 | 0·4679 | 1 |
|  | (95%CI) | [·3516,·6389] | [·0014,·0701] | [·0061,·1117] | [·3297,·6112] |  |
|  | Number of participants | 29 | 1 | 2 | 31 | 63 |
|  |  |  |  |  |  |  |
| Belgium | Proportion | 0·3671 | 0·1677 | 0·0997 | 0·3655 | 1 |
|  | (95%CI) | [·1089,·7336] | [·0232,·6312] | [·0129,·4836] | [·0955,·7587] |  |
|  | Number of participants | 3 | 1 | 1 | 2 | 7 |
|  |  |  |  |  |  |  |
| Croatia | Proportion | 0·5068 | 0 | 0·0959 | 0·3973 | 1 |
|  | (95%CI) | [·2082,·8007] |  | [·0129,·4624] | [·1364,·7334] |  |
|  | Number of participants | 5 | 0 | 1 | 3 | 9 |
|  |  |  |  |  |  |  |
| Cyprus | Proportion | 0·36 | 0·0391 | 0·36 | 0·2409 | 1 |
|  | (95%CI) | [·1635,·6181] | [·0053,·2371] | [·1635,·6181] | [·088,·5106] |  |
|  | Number of participants | 6 | 1 | 6 | 4 | 17 |
|  |  |  |  |  |  |  |
| Czech Rep· | Proportion | 0·5005 | 0 | 0·1314 | 0·3681 | 1 |
|  | (95%CI) | [·1844,·8162] |  | [·0285,·438] | [·0864,·7821] |  |
|  | Number of participants | 8 | 0 | 2 | 2 | 12 |
|  |  |  |  |  |  |  |
| Finland | Proportion | 0·2807 | 0 | 0 | 0·7193 | 1 |
|  | (95%CI) | [·0838,·6248] |  |  | [·3752,·9162] |  |
|  | Number of participants | 4 | 0 | 0 | 6 | 10 |
|  |  |  |  |  |  |  |
| France | Proportion | 0·3763 | 0 | 0·0339 | 0·5898 | 1 |
|  | (95%CI) | [·2649,·5025] |  | [·0048,·2029] | [·4609,·7074] |  |
|  | Number of participants | 29 | 0 | 1 | 40 | 70 |
|  |  |  |  |  |  |  |
| Germany | Proportion | 0·6284 | 0·0382 | 0·014 | 0·3194 | 1 |
|  | (95%CI) | [·4705,·7629] | [·0095,·1411] | [·0019,·0936] | [·1896,·4849] |  |
|  | Number of participants | 41 | 2 | 1 | 15 | 59 |
|  |  |  |  |  |  |  |
| Greece | Proportion | 0·2522 | 0·0766 | 0·0576 | 0·6136 | 1 |
|  | (95%CI) | [·1472,·3972] | [·0242,·2173] | [·0142,·2059] | [·4634,·7448] |  |
|  | Number of participants | 12 | 3 | 2 | 30 | 47 |
|  |  |  |  |  |  |  |
| Hungary | Proportion | 0·463 | 0·045 | 0·0285 | 0·4636 | 1 |
|  | (95%CI) | [·3207,·6115] | [·0134,·1407] | [·007,·1084] | [·3193,·6142] |  |
|  | Number of participants | 27 | 3 | 2 | 29 | 61 |
|  |  |  |  |  |  |  |
| Israel | Proportion | 0·5549 | 0 | 0 | 0·4451 | 1 |
|  | (95%CI) | [·3274,·7614] |  |  | [·2386,·6726] |  |
|  | Number of participants | 10 | 0 | 0 | 8 | 18 |
|  |  |  |  |  |  |  |
| Italy | Proportion | 0·5035 | 0·0123 | 0·0184 | 0·4658 | 1 |
|  | (95%CI) | [·3885,·6181] | [·003,·0484] | [·0059,·0564] | [·3529,·5824] |  |
|  | Number of participants | 54 | 2 | 3 | 52 | 111 |
|  |  |  |  |  |  |  |
| Latvia | Proportion | 0·6853 | 0 | 0 | 0·3147 | 1 |
|  | (95%CI) | [·1638,·9603] |  |  | [·0397,·8362] |  |
|  | Number of participants | 2 | 0 | 0 | 1 | 3 |
|  |  |  |  |  |  |  |
| Lithuania | Proportion | 1 | 0 | 0 | 0 | 1 |
|  | (95%CI) |  |  |  |  |  |
|  | Number of participants | 4 | 0 | 0 | 0 | 4 |
|  |  |  |  |  |  |  |
| Poland | Proportion | 0·0161 | 0·0519 | 0 | 0·932 | 1 |
|  | (95%CI) | [·0037,·0666] | [·0094,·2395] |  | [·7688,·9826] |  |
|  | Number of participants | 2 | 2 | 0 | 30 | 34 |
|  |  |  |  |  |  |  |
| Slovenia | Proportion | 0·626 | 0·0088 | 0·0626 | 0·3026 | 1 |
|  | (95%CI) | [·4954,·7405] | [·0012,·0602] | [·0229,·1601] | [·1986,·4318] |  |
|  | Number of participants | 40 | 1 | 4 | 23 | 68 |
|  |  |  |  |  |  |  |
| Spain | Proportion | 0·21 | 0·0198 | 0·0155 | 0·7548 | 1 |
|  | (95%CI) | [·1445,·2949] | [·0057,·0657] | [·0037,·0633] | [·6671,·8254] |  |
|  | Number of participants | 29 | 3 | 2 | 92 | 126 |
|  |  |  |  |  |  |  |
| Switzerland | Proportion | 0·68 | 0·0218 | 0·2025 | 0·0956 | 1 |
|  | (95%CI) | [·4844,·8278] | [·003,·1415] | [·0834,·4149] | [·0352,·2349] |  |
|  | Number of participants | 22 | 1 | 5 | 4 | 32 |
|  |  |  |  |  |  |  |
| Ukraine | Proportion | 0·3173 | 0·0856 | 0·0125 | 0·5846 | 1 |
|  | (95%CI) | [·1744,·5057] | [·0327,·2056] | [·0017,·0853] | [·4078,·742] |  |
|  | Number of participants | 12 | 5 | 1 | 26 | 44 |
|  |  |  |  |  |  |  |
| Total | Proportion | 0·4202 | 0·0274 | 0·043 | 0·5095 | 1 |
|  | (95%CI) | [·3812,·4602] | [·018,·0415] | [·0297,·0617] | [·4692,·5496] |  |
|  | Number of participants | 339 | 25 | 33 | 398 | 795 |

| **Country** |  | **CRP** | | | | |
| --- | --- | --- | --- | --- | --- | --- |
|  |  | I would not use the test | I would use both POCT and lab versions of the test | I would use only the lab version of the test | I would use only the POCT version | Total |
|  |  |  |  |  |  |  |
| Austria | Proportion | 0·0574 | 0·0294 | 0·0769 | 0·8363 | 1 |
|  | (95%CI) | [·0205,·1504] | [·0068,·1176] | [·0327,·1702] | [·721,·9099] |  |
|  | Number of participants | 4 | 2 | 6 | 51 | 63 |
|  |  |  |  |  |  |  |
| Belgium | Proportion | 0·0997 | 0·2674 | 0·1677 | 0·4652 | 1 |
|  | (95%CI) | [·0129,·4836] | [·0622,·6678] | [·0232,·6312] | [·1529,·8074] |  |
|  | Number of participants | 1 | 2 | 1 | 3 | 7 |
|  |  |  |  |  |  |  |
| Croatia | Proportion | 0 | 0 | 0·2877 | 0·7123 | 1 |
|  | (95%CI) |  |  | [·0897,·6233] | [·3767,·9103] |  |
|  | Number of participants | 0 | 0 | 3 | 6 | 9 |
|  |  |  |  |  |  |  |
| Cyprus | Proportion | 0·08 | 0·0391 | 0·3991 | 0·4817 | 1 |
|  | (95%CI) | [·0113,·3989] | [·0053,·2371] | [·1921,·6499] | [·2524,·7189] |  |
|  | Number of participants | 1 | 1 | 7 | 8 | 17 |
|  |  |  |  |  |  |  |
| Czech Rep· | Proportion | 0 | 0·0531 | 0 | 0·9469 | 1 |
|  | (95%CI) |  | [·0067,·317] |  | [·683,·9933] |  |
|  | Number of participants | 0 | 1 | 0 | 11 | 12 |
|  |  |  |  |  |  |  |
| Finland | Proportion | 0 | 0·3421 | 0·0614 | 0·5965 | 1 |
|  | (95%CI) |  | [·0612,·8056] | [·0076,·3571] | [·1908,·9026] |  |
|  | Number of participants | 0 | 1 | 1 | 8 | 10 |
|  |  |  |  |  |  |  |
| France | Proportion | 0·1891 | 0·0476 | 0·0492 | 0·7142 | 1 |
|  | (95%CI) | [·1145,·296] | [·0107,·1878] | [·0182,·1257] | [·5901,·8127] |  |
|  | Number of participants | 15 | 2 | 4 | 49 | 70 |
|  |  |  |  |  |  |  |
| Germany | Proportion | 0·2001 | 0·1109 | 0·0798 | 0·6093 | 1 |
|  | (95%CI) | [·1162,·3224] | [·0276,·3536] | [·0328,·1815] | [·4556,·744] |  |
|  | Number of participants | 13 | 3 | 5 | 38 | 59 |
|  |  |  |  |  |  |  |
| Greece | Proportion | 0·0381 | 0·2184 | 0·303 | 0·4405 | 1 |
|  | (95%CI) | [·0095,·1407] | [·1203,·3633] | [·1869,·4512] | [·3048,·5858] |  |
|  | Number of participants | 2 | 10 | 14 | 21 | 47 |
|  |  |  |  |  |  |  |
| Hungary | Proportion | 0·1006 | 0·0835 | 0·1052 | 0·7107 | 1 |
|  | (95%CI) | [·0385,·2381] | [·0326,·1977] | [·0502,·2071] | [·5667,·8219] |  |
|  | Number of participants | 5 | 5 | 8 | 43 | 61 |
|  |  |  |  |  |  |  |
| Israel | Proportion | 0·2088 | 0·1047 | 0·2376 | 0·4489 | 1 |
|  | (95%CI) | [·079,·448] | [·0258,·3405] | [·092,·4894] | [·2414,·6758] |  |
|  | Number of participants | 4 | 2 | 4 | 8 | 18 |
|  |  |  |  |  |  |  |
| Italy | Proportion | 0·0491 | 0·0307 | 0·0787 | 0·8415 | 1 |
|  | (95%CI) | [·0241,·0973] | [·0126,·0729] | [·0407,·1469] | [·7627,·8977] |  |
|  | Number of participants | 8 | 5 | 10 | 88 | 111 |
|  |  |  |  |  |  |  |
| Latvia | Proportion | 0 | 0·6853 | 0 | 0·3147 | 1 |
|  | (95%CI) |  | [·1638,·9603] |  | [·0397,·8362] |  |
|  | Number of participants | 0 | 2 | 0 | 1 | 3 |
|  |  |  |  |  |  |  |
| Lithuania | Proportion | 0 | 0 | 0·8577 | 0·1423 | 1 |
|  | (95%CI) |  |  | [·3519,·9853] | [·0147,·6481] |  |
|  | Number of participants | 0 | 0 | 3 | 1 | 4 |
|  |  |  |  |  |  |  |
| Poland | Proportion | 0·1206 | 0·0603 | 0 | 0·8191 | 1 |
|  | (95%CI) | [·0288,·3879] | [·0134,·2327] |  | [·5754,·938] |  |
|  | Number of participants | 2 | 3 | 0 | 29 | 34 |
|  |  |  |  |  |  |  |
| Slovenia | Proportion | 0·0263 | 0·0509 | 0·5099 | 0·413 | 1 |
|  | (95%CI) | [·0083,·0796] | [·0143,·1649] | [·3814,·637] | [·2947,·5422] |  |
|  | Number of participants | 3 | 3 | 31 | 31 | 68 |
|  |  |  |  |  |  |  |
| Spain | Proportion | 0·1871 | 0·1182 | 0·0893 | 0·6055 | 1 |
|  | (95%CI) | [·1235,·2732] | [·0667,·2008] | [·0502,·1538] | [·5091,·6943] |  |
|  | Number of participants | 24 | 13 | 13 | 76 | 126 |
|  |  |  |  |  |  |  |
| Switzerland | Proportion | 0·2391 | 0 | 0·1343 | 0·6266 | 1 |
|  | (95%CI) | [·1202,·4196] |  | [·0418,·3556] | [·4336,·7862] |  |
|  | Number of participants | 9 | 0 | 3 | 20 | 32 |
|  |  |  |  |  |  |  |
| Ukraine | Proportion | 0·2265 | 0·0908 | 0·1461 | 0·5365 | 1 |
|  | (95%CI) | [·1083,·4138] | [·0353,·2144] | [·0564,·3291] | [·3641,·7006] |  |
|  | Number of participants | 9 | 5 | 5 | 25 | 44 |
|  |  |  |  |  |  |  |
| Total | Proportion | 0·1199 | 0·0819 | 0·1461 | 0·6521 | 1 |
|  | (95%CI) | [·0974,·1467] | [·0612,·1087] | [·1213,·175] | [·6135,·6889] |  |
|  | Number of participants | 100 | 60 | 118 | 517 | 795 |

| **Country** |  | **Procalcitonin** | | | | |
| --- | --- | --- | --- | --- | --- | --- |
|  |  | I would not use the test | I would use both POCT and lab versions of the test | I would use only the lab version of the test | I would use only the POCT version | Total |
|  |  |  |  |  |  |  |
| Austria | Proportion | 0·6056 | 0·0086 | 0·0171 | 0·3687 | 1 |
|  | (95%CI) | [·4654,·7303] | [·0012,·0592] | [·0042,·0673] | [·2482,·5081] |  |
|  | Number of participants | 34 | 1 | 2 | 26 | 63 |
|  |  |  |  |  |  |  |
| Belgium | Proportion | 0·6962 | 0 | 0·1677 | 0·1361 | 1 |
|  | (95%CI) | [·2988,·9249] |  | [·0232,·6312] | [·0182,·5722] |  |
|  | Number of participants | 5 | 0 | 1 | 1 | 7 |
|  |  |  |  |  |  |  |
| Croatia | Proportion | 0·6773 | 0 | 0·0959 | 0·2268 | 1 |
|  | (95%CI) | [·3357,·8971] |  | [·0129,·4624] | [·0556,·5938] |  |
|  | Number of participants | 6 | 0 | 1 | 2 | 9 |
|  |  |  |  |  |  |  |
| Cyprus | Proportion | 0·4817 | 0 | 0·2366 | 0·2817 | 1 |
|  | (95%CI) | [·2524,·7189] |  | [·0932,·4832] | [·1087,·5578] |  |
|  | Number of participants | 8 | 0 | 5 | 4 | 17 |
|  |  |  |  |  |  |  |
| Czech Rep· | Proportion | 0·9469 | 0 | 0·0531 | 0 | 1 |
|  | (95%CI) | [·683,·9933] |  | [·0067,·317] |  |  |
|  | Number of participants | 11 | 0 | 1 | 0 | 12 |
|  |  |  |  |  |  |  |
| Finland | Proportion | 0·9035 | 0 | 0 | 0·0965 | 1 |
|  | (95%CI) | [·5226,·9877] |  |  | [·0123,·4774] |  |
|  | Number of participants | 9 | 0 | 0 | 1 | 10 |
|  |  |  |  |  |  |  |
| France | Proportion | 0·3352 | 0·0449 | 0·0921 | 0·5278 | 1 |
|  | (95%CI) | [·2266,·4646] | [·0096,·186] | [·0377,·2081] | [·3991,·6528] |  |
|  | Number of participants | 24 | 2 | 6 | 38 | 70 |
|  |  |  |  |  |  |  |
| Germany | Proportion | 0·5941 | 0·0321 | 0·0737 | 0·3001 | 1 |
|  | (95%CI) | [·4554,·7193] | [·0078,·1232] | [·0302,·1688] | [·1935,·4339] |  |
|  | Number of participants | 33 | 2 | 5 | 19 | 59 |
|  |  |  |  |  |  |  |
| Greece | Proportion | 0·4147 | 0·138 | 0·1761 | 0·2712 | 1 |
|  | (95%CI) | [·2826,·5604] | [·0622,·2786] | [·0892,·318] | [·1624,·4168] |  |
|  | Number of participants | 20 | 6 | 8 | 13 | 47 |
|  |  |  |  |  |  |  |
| Hungary | Proportion | 0·3303 | 0·0285 | 0·1207 | 0·5205 | 1 |
|  | (95%CI) | [·2103,·4773] | [·007,·1084] | [·0574,·2363] | [·3734,·6641] |  |
|  | Number of participants | 22 | 2 | 8 | 29 | 61 |
|  |  |  |  |  |  |  |
| Israel | Proportion | 0·7768 | 0·0458 | 0·0639 | 0·1135 | 1 |
|  | (95%CI) | [·5305,·9147] | [·0063,·266] | [·009,·3404] | [·0282,·3611] |  |
|  | Number of participants | 14 | 1 | 1 | 2 | 18 |
|  |  |  |  |  |  |  |
| Italy | Proportion | 0·5211 | 0·0184 | 0·0603 | 0·4002 | 1 |
|  | (95%CI) | [·4052,·6347] | [·0059,·0564] | [·0275,·1272] | [·2915,·5197] |  |
|  | Number of participants | 57 | 3 | 7 | 44 | 111 |
|  |  |  |  |  |  |  |
| Latvia | Proportion | 0·6294 | 0 | 0·3706 | 0 | 1 |
|  | (95%CI) | [·1328,·9496] |  | [·0504,·8672] |  |  |
|  | Number of participants | 2 | 0 | 1 | 0 | 3 |
|  |  |  |  |  |  |  |
| Lithuania | Proportion | 1 | 0 | 0 | 0 | 1 |
|  | (95%CI) |  |  |  |  |  |
|  | Number of participants | 4 | 0 | 0 | 0 | 4 |
|  |  |  |  |  |  |  |
| Poland | Proportion | 0·1444 | 0·0603 | 0·0084 | 0·7869 | 1 |
|  | (95%CI) | [·0428,·389] | [·0134,·2327] | [·0011,·0603] | [·5553,·9161] |  |
|  | Number of participants | 5 | 3 | 1 | 25 | 34 |
|  |  |  |  |  |  |  |
| Slovenia | Proportion | 0·6597 | 0·0218 | 0·096 | 0·2225 | 1 |
|  | (95%CI) | [·5237,·7736] | [·0053,·086] | [·0348,·2381] | [·1331,·3479] |  |
|  | Number of participants | 46 | 2 | 4 | 16 | 68 |
|  |  |  |  |  |  |  |
| Spain | Proportion | 0·2625 | 0·1165 | 0·049 | 0·5719 | 1 |
|  | (95%CI) | [·1873,·3549] | [·0649,·2005] | [·0221,·105] | [·4754,·6633] |  |
|  | Number of participants | 34 | 12 | 7 | 73 | 126 |
|  |  |  |  |  |  |  |
| Switzerland | Proportion | 0·9001 | 0 | 0 | 0·0999 | 1 |
|  | (95%CI) | [·7566,·9632] |  |  | [·0368,·2434] |  |
|  | Number of participants | 28 | 0 | 0 | 4 | 32 |
|  |  |  |  |  |  |  |
| Ukraine | Proportion | 0·6221 | 0·0303 | 0·1211 | 0·2265 | 1 |
|  | (95%CI) | [·4458,·7711] | [·0072,·1179] | [·0414,·3055] | [·1157,·3958] |  |
|  | Number of participants | 27 | 2 | 4 | 11 | 44 |
|  |  |  |  |  |  |  |
| Total | Proportion | 0·4939 | 0·045 | 0·0777 | 0·3834 | 1 |
|  | (95%CI) | [·4537,·5342] | [·0315,·064] | [·0595,·1008] | [·3449,·4234] |  |
|  | Number of participants | 389 | 36 | 62 | 308 | 795 |

| **Country** |  | **Full blood count** | | | | |
| --- | --- | --- | --- | --- | --- | --- |
|  |  | I would not use the test | I would use both POCT and lab versions of the test | I would use only the lab version of the test | I would use only the POCT version | Total |
|  |  |  |  |  |  |  |
| Austria | Proportion | 0·0749 | 0·0499 | 0·1142 | 0·7611 | 1 |
|  | (95%CI) | [·0298,·1759] | [·0176,·133] | [·0567,·2164] | [·6353,·8535] |  |
|  | Number of participants | 5 | 4 | 9 | 45 | 63 |
|  |  |  |  |  |  |  |
| Belgium | Proportion | 0·0997 | 0·2674 | 0·0997 | 0·5332 | 1 |
|  | (95%CI) | [·0129,·4836] | [·0622,·6678] | [·0129,·4836] | [·1966,·8421] |  |
|  | Number of participants | 1 | 2 | 1 | 3 | 7 |
|  |  |  |  |  |  |  |
| Croatia | Proportion | 0 | 0 | 0·5145 | 0·4855 | 1 |
|  | (95%CI) |  |  | [·2126,·8061] | [·1939,·7874] |  |
|  | Number of participants | 0 | 0 | 5 | 4 | 9 |
|  |  |  |  |  |  |  |
| Cyprus | Proportion | 0·1626 | 0·0391 | 0·5574 | 0·2409 | 1 |
|  | (95%CI) | [·0419,·4628] | [·0053,·2371] | [·3115,·7781] | [·088,·5106] |  |
|  | Number of participants | 2 | 1 | 10 | 4 | 17 |
|  |  |  |  |  |  |  |
| Czech Re | Proportion | 0·5778 | 0 | 0·3691 | 0·0531 | 1 |
|  | (95%CI) | [·2547,·8456] |  | [·1314,·6935] | [·0067,·317] |  |
|  | Number of participants | 5 | 0 | 6 | 1 | 12 |
|  |  |  |  |  |  |  |
| Finland | Proportion | 0·2193 | 0·3421 | 0·0614 | 0·3772 | 1 |
|  | (95%CI) | [·058,·5619] | [·0612,·8056] | [·0076,·3571] | [·1215,·7262] |  |
|  | Number of participants | 3 | 1 | 1 | 5 | 10 |
|  |  |  |  |  |  |  |
| France | Proportion | 0·3946 | 0·011 | 0·2378 | 0·3566 | 1 |
|  | (95%CI) | [·2805,·5214] | [·0015,·0746] | [·1436,·3673] | [·2405,·4923] |  |
|  | Number of participants | 30 | 1 | 16 | 23 | 70 |
|  |  |  |  |  |  |  |
| Germany | Proportion | 0·2385 | 0·0979 | 0·1727 | 0·4909 | 1 |
|  | (95%CI) | [·1447,·367] | [·0205,·3605] | [·0955,·2921] | [·3529,·6304] |  |
|  | Number of participants | 15 | 2 | 11 | 31 | 59 |
|  |  |  |  |  |  |  |
| Greece | Proportion | 0·0571 | 0·2294 | 0·3576 | 0·3559 | 1 |
|  | (95%CI) | [·0184,·1637] | [·1266,·3794] | [·2335,·5044] | [·2316,·5032] |  |
|  | Number of participants | 3 | 10 | 17 | 17 | 47 |
|  |  |  |  |  |  |  |
| Hungary | Proportion | 0·1541 | 0·089 | 0·1906 | 0·5662 | 1 |
|  | (95%CI) | [·0727,·2975] | [·0356,·2054] | [·1103,·3091] | [·4187,·7029] |  |
|  | Number of participants | 8 | 5 | 15 | 33 | 61 |
|  |  |  |  |  |  |  |
| Israel | Proportion | 0·1592 | 0·1687 | 0·2376 | 0·4345 | 1 |
|  | (95%CI) | [·0512,·3993] | [·0547,·4155] | [·092,·4894] | [·2309,·6628] |  |
|  | Number of participants | 3 | 3 | 4 | 8 | 18 |
|  |  |  |  |  |  |  |
| Italy | Proportion | 0·206 | 0·0616 | 0·1759 | 0·5565 | 1 |
|  | (95%CI) | [·1251,·3202] | [·0308,·1193] | [·1138,·2619] | [·4416,·6657] |  |
|  | Number of participants | 22 | 9 | 23 | 57 | 111 |
|  |  |  |  |  |  |  |
| Latvia | Proportion | 0 | 0·3147 | 0·6853 | 0 | 1 |
|  | (95%CI) |  | [·0397,·8362] | [·1638,·9603] |  |  |
|  | Number of participants | 0 | 1 | 2 | 0 | 3 |
|  |  |  |  |  |  |  |
| Lithuania | Proportion | 0 | 0 | 0·8577 | 0·1423 | 1 |
|  | (95%CI) |  |  | [·3519,·9853] | [·0147,·6481] |  |
|  | Number of participants | 0 | 0 | 3 | 1 | 4 |
|  |  |  |  |  |  |  |
| Poland | Proportion | 0·0154 | 0·0687 | 0·136 | 0·78 | 1 |
|  | (95%CI) | [·0036,·0637] | [·0177,·2315] | [·0377,·3872] | [·5503,·9112] |  |
|  | Number of participants | 2 | 4 | 4 | 24 | 34 |
|  |  |  |  |  |  |  |
| Slovenia | Proportion | 0·0175 | 0·0717 | 0·552 | 0·3588 | 1 |
|  | (95%CI) | [·0043,·0683] | [·0291,·166] | [·4232,·6742] | [·2484,·4865] |  |
|  | Number of participants | 2 | 5 | 33 | 28 | 68 |
|  |  |  |  |  |  |  |
| Spain | Proportion | 0·3529 | 0·0882 | 0·2625 | 0·2963 | 1 |
|  | (95%CI) | [·2671,·4494] | [·0458,·1633] | [·1873,·3549] | [·2182,·3885] |  |
|  | Number of participants | 44 | 10 | 34 | 38 | 126 |
|  |  |  |  |  |  |  |
| Switzerland | Proportion | 0·2651 | 0 | 0·1821 | 0·5527 | 1 |
|  | (95%CI) | [·1389,·4466] |  | [·0726,·3878] | [·367,·7248] |  |
|  | Number of participants | 10 | 0 | 5 | 17 | 32 |
|  |  |  |  |  |  |  |
| Ukraine | Proportion | 0·2067 | 0·0783 | 0·3904 | 0·3246 | 1 |
|  | (95%CI) | [·0926,·3996] | [·0275,·2033] | [·244,·5596] | [·1796,·5133] |  |
|  | Number of participants | 7 | 4 | 21 | 12 | 44 |
|  |  |  |  |  |  |  |
| Total | Proportion | 0·2032 | 0·0805 | 0·2623 | 0·454 | 1 |
|  | (95%CI) | [·173,·2371] | [·0605,·1065] | [·23,·2974] | [·4139,·4946] |  |
|  | Number of participants | 162 | 62 | 220 | 351 | 795 |

| **Country** |  | **Blood gas analysis (with or without lactate)** | | |
| --- | --- | --- | --- | --- |
|  |  | I would not use the test | I would use only the POCT version | Total |
|  |  |  |  |  |
| Austria | Proportion | 0·7965 | 0·2035 | 1 |
|  | (95%CI) | [·6277,·9009] | [·0991,·3723] |  |
|  | Number of participants | 52 | 11 | 63 |
|  |  |  |  |  |
| Belgium | Proportion | 0·5965 | 0·4035 | 1 |
|  | (95%CI) | [·2371,·8755] | [·1245,·7629] |  |
|  | Number of participants | 4 | 3 | 7 |
|  |  |  |  |  |
| Croatia | Proportion | 0·6773 | 0·3227 | 1 |
|  | (95%CI) | [·3357,·8971] | [·1029,·6643] |  |
|  | Number of participants | 6 | 3 | 9 |
|  |  |  |  |  |
| Cyprus | Proportion | 0·9609 | 0·0391 | 1 |
|  | (95%CI) | [·7629,·9947] | [·0053,·2371] |  |
|  | Number of participants | 16 | 1 | 17 |
|  |  |  |  |  |
| Czech Re | Proportion | 0·8155 | 0·1845 | 1 |
|  | (95%CI) | [·5094,·9495] | [·0505,·4906] |  |
|  | Number of participants | 9 | 3 | 12 |
|  |  |  |  |  |
| Finland | Proportion | 0·9386 | 0·0614 | 1 |
|  | (95%CI) | [·6429,·9924] | [·0076,·3571] |  |
|  | Number of participants | 9 | 1 | 10 |
|  |  |  |  |  |
| France | Proportion | 0·9058 | 0·0942 | 1 |
|  | (95%CI) | [·7889,·9611] | [·0389,·2111] |  |
|  | Number of participants | 64 | 6 | 70 |
|  |  |  |  |  |
| Germany | Proportion | 0·8411 | 0·1589 | 1 |
|  | (95%CI) | [·7232,·9147] | [·0853,·2768] |  |
|  | Number of participants | 49 | 10 | 59 |
|  |  |  |  |  |
| Greece | Proportion | 0·8472 | 0·1528 | 1 |
|  | (95%CI) | [·7082,·9269] | [·0731,·2918] |  |
|  | Number of participants | 40 | 7 | 47 |
|  |  |  |  |  |
| Hungary | Proportion | 0·6304 | 0·3696 | 1 |
|  | (95%CI) | [·4698,·7666] | [·2334,·5302] |  |
|  | Number of participants | 39 | 22 | 61 |
|  |  |  |  |  |
| Israel | Proportion | 0·8721 | 0·1279 | 1 |
|  | (95%CI) | [·6095,·9675] | [·0325,·3905] |  |
|  | Number of participants | 16 | 2 | 18 |
|  |  |  |  |  |
| Italy | Proportion | 0·8322 | 0·1678 | 1 |
|  | (95%CI) | [·714,·9079] | [·0921,·286] |  |
|  | Number of participants | 96 | 15 | 111 |
|  |  |  |  |  |
| Latvia | Proportion | 0·6853 | 0·3147 | 1 |
|  | (95%CI) | [·1638,·9603] | [·0397,·8362] |  |
|  | Number of participants | 2 | 1 | 3 |
|  |  |  |  |  |
| Lithuania | Proportion | 1 | 0 | 1 |
|  | (95%CI) |  |  |  |
|  | Number of participants | 4 | 0 | 4 |
|  |  |  |  |  |
| Poland | Proportion | 0·3699 | 0·6301 | 1 |
|  | (95%CI) | [·18,·6109] | [·3891,·82] |  |
|  | Number of participants | 16 | 18 | 34 |
|  |  |  |  |  |
| Slovenia | Proportion | 0·9151 | 0·0849 | 1 |
|  | (95%CI) | [·7835,·9698] | [·0302,·2165] |  |
|  | Number of participants | 64 | 4 | 68 |
|  |  |  |  |  |
| Spain | Proportion | 0·8693 | 0·1307 | 1 |
|  | (95%CI) | [·7842,·924] | [·076,·2158] |  |
|  | Number of participants | 112 | 14 | 126 |
|  |  |  |  |  |
| Switzerland | Proportion | 0·91 | 0·09 | 1 |
|  | (95%CI) | [·7378,·9732] | [·0268,·2622] |  |
|  | Number of participants | 29 | 3 | 32 |
|  |  |  |  |  |
| Ukraine | Proportion | 0·778 | 0·222 | 1 |
|  | (95%CI) | [·6053,·889] | [·111,·3947] |  |
|  | Number of participants | 33 | 10 | 43 |
|  |  |  |  |  |
| Total | Proportion | 0·8162 | 0·1838 | 1 |
|  | (95%CI) | [·7801,·8475] | [·1525,·2199] |  |
|  | Number of participants | 660 | 134 | 794 |

| **Country** |  | **Lactate** | | | | |
| --- | --- | --- | --- | --- | --- | --- |
|  |  | I would not use the test | I would use both POCT and lab versions of the test | I would use only the lab version of the test | I would use only the POCT version | Total |
|  |  |  |  |  |  |  |
| Austria | Proportion | 0·8981 | 0·0277 | 0·0468 | 0·0274 | 1 |
|  | (95%CI) | [·7967,·952] | [·0062,·1149] | [·0142,·1438] | [·0086,·0837] |  |
|  | Number of participants | 55 | 2 | 3 | 3 | 63 |
|  |  |  |  |  |  |  |
| Belgium | Proportion | 0·8323 | 0 | 0·1677 | 0 | 1 |
|  | (95%CI) | [·3688,·9768] |  | [·0232,·6312] |  |  |
|  | Number of participants | 6 | 0 | 1 | 0 | 7 |
|  |  |  |  |  |  |  |
| Croatia | Proportion | 0·8082 | 0 | 0·0959 | 0·0959 | 1 |
|  | (95%CI) | [·4621,·9539] |  | [·0129,·4624] | [·0129,·4624] |  |
|  | Number of participants | 7 | 0 | 1 | 1 | 9 |
|  |  |  |  |  |  |  |
| Cyprus | Proportion | 0·9609 | 0 | 0·0391 | 0 | 1 |
|  | (95%CI) | [·7629,·9947] |  | [·0053,·2371] |  |  |
|  | Number of participants | 16 | 0 | 1 | 0 | 17 |
|  |  |  |  |  |  |  |
| Czech Re | Proportion | 0·9469 | 0·0531 | 0 | 0 | 1 |
|  | (95%CI) | [·683,·9933] | [·0067,·317] |  |  |  |
|  | Number of participants | 11 | 1 | 0 | 0 | 12 |
|  |  |  |  |  |  |  |
| Finland | Proportion | 1 | 0 | 0 | 0 | 1 |
|  | (95%CI) |  |  |  |  |  |
|  | Number of participants | 10 | 0 | 0 | 0 | 10 |
|  |  |  |  |  |  |  |
| France | Proportion | 0·9416 | 0 | 0·0474 | 0·011 | 1 |
|  | (95%CI) | [·8132,·9835] |  | [·0106,·1876] | [·0015,·0746] |  |
|  | Number of participants | 67 | 0 | 2 | 1 | 70 |
|  |  |  |  |  |  |  |
| Germany | Proportion | 0·8881 | 0·0331 | 0·0788 | 0 | 1 |
|  | (95%CI) | [·6464,·9718] | [·0081,·1257] | [·0117,·3828] |  |  |
|  | Number of participants | 56 | 2 | 1 | 0 | 59 |
|  |  |  |  |  |  |  |
| Greece | Proportion | 0·8663 | 0·0343 | 0·0614 | 0·0381 | 1 |
|  | (95%CI) | [·7281,·94] | [·0049,·2056] | [·0197,·1752] | [·0095,·1407] |  |
|  | Number of participants | 41 | 1 | 3 | 2 | 47 |
|  |  |  |  |  |  |  |
| Hungary | Proportion | 0·865 | 0 | 0·0725 | 0·0625 | 1 |
|  | (95%CI) | [·7477,·9327] |  | [·0266,·1831] | [·0238,·1537] |  |
|  | Number of participants | 52 | 0 | 4 | 5 | 61 |
|  |  |  |  |  |  |  |
| Israel | Proportion | 0·9542 | 0 | 0·0458 | 0 | 1 |
|  | (95%CI) | [·734,·9937] |  | [·0063,·266] |  |  |
|  | Number of participants | 17 | 0 | 1 | 0 | 18 |
|  |  |  |  |  |  |  |
| Italy | Proportion | 0·8849 | 0·0123 | 0·0362 | 0·0667 | 1 |
|  | (95%CI) | [·8068,·934] | [·003,·0484] | [·0143,·0884] | [·0307,·1389] |  |
|  | Number of participants | 97 | 2 | 5 | 7 | 111 |
|  |  |  |  |  |  |  |
| Latvia | Proportion | 1 | 0 | 0 | 0 | 1 |
|  | (95%CI) |  |  |  |  |  |
|  | Number of participants | 3 | 0 | 0 | 0 | 3 |
|  |  |  |  |  |  |  |
| Lithuania | Proportion | 1 | 0 | 0 | 0 | 1 |
|  | (95%CI) |  |  |  |  |  |
|  | Number of participants | 4 | 0 | 0 | 0 | 4 |
|  |  |  |  |  |  |  |
| Poland | Proportion | 0·8374 | 0·0519 | 0·0771 | 0·0336 | 1 |
|  | (95%CI) | [·6037,·9457] | [·0094,·2395] | [·0109,·388] | [·0114,·0948] |  |
|  | Number of participants | 27 | 2 | 1 | 4 | 34 |
|  |  |  |  |  |  |  |
| Slovenia | Proportion | 0·891 | 0·0131 | 0·0829 | 0·0131 | 1 |
|  | (95%CI) | [·7721,·9517] | [·0018,·0876] | [·0309,·2044] | [·0018,·0876] |  |
|  | Number of participants | 62 | 1 | 4 | 1 | 68 |
|  |  |  |  |  |  |  |
| Spain | Proportion | 0·9319 | 0·01 | 0·0055 | 0·0526 | 1 |
|  | (95%CI) | [·8705,·9654] | [·0014,·0675] | [7·7e-04,·0386] | [·0243,·1099] |  |
|  | Number of participants | 117 | 1 | 1 | 7 | 126 |
|  |  |  |  |  |  |  |
| Switzerland | Proportion | 0·884 | 0 | 0·0218 | 0·0942 | 1 |
|  | (95%CI) | [·7138,·9588] |  | [·003,·1415] | [·0286,·2689] |  |
|  | Number of participants | 28 | 0 | 1 | 3 | 32 |
|  |  |  |  |  |  |  |
| Ukraine | Proportion | 0·8413 | 0 | 0 | 0·1587 | 1 |
|  | (95%CI) | [·6624,·9348] |  |  | [·0652,·3376] |  |
|  | Number of participants | 38 | 0 | 0 | 6 | 44 |
|  |  |  |  |  |  |  |
| Total | Proportion | 0·8973 | 0·0141 | 0·0448 | 0·0439 | 1 |
|  | (95%CI) | [·8696,·9196] | [·0077,·0259] | [·0291,·0683] | [·0311,·0615] |  |
|  | Number of participants | 714 | 12 | 29 | 40 | 795 |

# **Hospitals**

| **Country** |  | **Urine dipstick** | | | | |
| --- | --- | --- | --- | --- | --- | --- |
|  |  | I would not use the test | I would use both POCT and lab versions of the test | I would use only the lab version of the test | I would use only the POCT version | Total |
| Austria | Proportion | 0·108 | 0·8116 | 0·0152 | 0·0651 | 1 |
|  | (95%CI) | [·054,·2044] | [·6859,·8948] | [·0021,·1011] | [·0193,·1976] |  |
|  | Number of participants | 11 | 60 | 1 | 3 | 75 |
|  |  |  |  |  |  |  |
| Belgium | Proportion | 0·1071 | 0·4579 | 0·2574 | 0·1777 | 1 |
|  | (95%CI) | [·0467,·227] | [·3005,·6241] | [·1448,·415] | [·0918,·316] |  |
|  | Number of participants | 6 | 22 | 12 | 11 | 51 |
|  |  |  |  |  |  |  |
| Bulgaria | Proportion | 0·44 | 0·12 | 0 | 0·44 | 1 |
|  | (95%CI) | [·0975,·8511] | [·0143,·5616] | | [·0975,·8511] |  |
|  | Number of participants | 2 | 1 | 0 | 2 | 5 |
|  |  |  |  |  |  |  |
| Croatia | Proportion | 0·1307 | 0·3322 | 0·1307 | 0·4064 | 1 |
|  | (95%CI) | [·0409,·3468] | [·1832,·5244] | [·0517,·2932] | [·2302,·6104] |  |
|  | Number of participants | 4 | 11 | 5 | 9 | 29 |
|  |  |  |  |  |  |  |
| Cyprus | Proportion | 0 | 0·5437 | 0 | 0·4563 | 1 |
|  | (95%CI) |  | [·2253,·83] |  | [·17,·7747] |  |
|  | Number of participants | 0 | 5 | 0 | 7 | 12 |
|  |  |  |  |  |  |  |
| Czech Re | Proportion | 0 | 0 | 1 | 0 | 1 |
|  | (95%CI) |  |  |  |  |  |
|  | Number of participants | 0 | 0 | 1 | 0 | 1 |
|  |  |  |  |  |  |  |
| Denmark | Proportion | 0 | 1 | 0 | 0 | 1 |
|  | (95%CI) |  |  |  |  |  |
|  | Number of participants | 0 | 5 | 0 | 0 | 5 |
|  |  |  |  |  |  |  |
| Finland | Proportion | 0·2321 | 0·4852 | 0·0549 | 0·2278 | 1 |
|  | (95%CI) | [·0992,·4534] | [·2927,·6822] | [·0077,·3018] | [·0972,·4472] |  |
|  | Number of participants | 5 | 13 | 1 | 5 | 24 |
|  |  |  |  |  |  |  |
| France | Proportion | 0·0208 | 0·9792 | 0 | 0 | 1 |
|  | (95%CI) | [·0028,·1369] | [·8631,·9972] | |  |  |
|  | Number of participants | 1 | 30 | 0 | 0 | 31 |
|  |  |  |  |  |  |  |
| Germany | Proportion | 0·1043 | 0·7791 | 0 | 0·1166 | 1 |
|  | (95%CI) | [·023,·3653] | [·5359,·9151] | | [·03,·3601] |  |
|  | Number of participants | 3 | 43 | 0 | 5 | 51 |
|  |  |  |  |  |  |  |
| Greece | Proportion | 0·0574 | 0·4147 | 0·2137 | 0·3142 | 1 |
|  | (95%CI) | [·0179,·1689] | [·2755,·569] | [·1202,·3509] | [·1975,·4603] |  |
|  | Number of participants | 4 | 24 | 14 | 22 | 64 |
|  |  |  |  |  |  |  |
| Hungary | Proportion | 0·1864 | 0·7119 | 0·0508 | 0·0508 | 1 |
|  | (95%CI) | [·046,·5214] | [·4169,·8951] | [·0069,·2931] | [·0069,·2931] |  |
|  | Number of participants | 2 | 10 | 1 | 1 | 14 |
|  |  |  |  |  |  |  |
| Ireland | Proportion | 0 | 1 | 0 | 0 | 1 |
|  | (95%CI) |  |  |  |  |  |
|  | Number of participants | 0 | 3 | 0 | 0 | 3 |
|  |  |  |  |  |  |  |
| Israel | Proportion | 0·8421 | 0·1579 | 0 | 0 | 1 |
|  | (95%CI) | [·2492,·9885] | [·0115,·7508] | |  |  |
|  | Number of participants | 1 | 1 | 0 | 0 | 2 |
|  |  |  |  |  |  |  |
| Italy | Proportion | 0·2617 | 0·7383 | 0 | 0 | 1 |
|  | (95%CI) | [·0895,·5612] | [·4388,·9105] | |  |  |
|  | Number of participants | 7 | 20 | 0 | 0 | 27 |
|  |  |  |  |  |  |  |
| Latvia | Proportion | 0·0444 | 0·6222 | 0·2333 | 0·1 | 1 |
|  | (95%CI) | [·0055,·2798] | [·2471,·8921] | [·035,·7184] | [·0212,·3628] |  |
|  | Number of participants | 1 | 5 | 1 | 2 | 9 |
|  |  |  |  |  |  |  |
| Lithuania | Proportion | 0 | 0·3646 | 0·1562 | 0·4792 | 1 |
|  | (95%CI) |  | [·1305,·6869] | [·0423,·4368] | [·1616,·8146] |  |
|  | Number of participants | 0 | 7 | 3 | 2 | 12 |
|  |  |  |  |  |  |  |
| Malta | Proportion | 0·1671 | 0·7062 | 0·0728 | 0·0539 | 1 |
|  | (95%CI) | [·0793,·3185] | [·544,·8289] | [·0235,·2039] | [·0127,·2013] |  |
|  | Number of participants | 7 | 27 | 3 | 2 | 39 |
|  |  |  |  |  |  |  |
| Netherlands | Proportion | 0·0702 | 0·4048 | 0·2828 | 0·2421 | 1 |
|  | (95%CI) | [·0212,·2083] | [·2722,·5529] | [·1666,·4375] | [·1395,·3864] |  |
|  | Number of participants | 3 | 22 | 12 | 13 | 50 |
|  |  |  |  |  |  |  |
| Norway | Proportion | 0·1511 | 0·7127 | 0·0448 | 0·0914 | 1 |
|  | (95%CI) | [·0789,·27] | [·5701,·8227] | [·0104,·1723] | [·0331,·2285] |  |
|  | Number of participants | 9 | 38 | 2 | 4 | 53 |
|  |  |  |  |  |  |  |
| Poland | Proportion | 0·458 | 0·2465 | 0·1667 | 0·1289 | 1 |
|  | (95%CI) | [·3264,·5957] | [·1433,·3901] | [·0907,·2861] | [·0578,·263] |  |
|  | Number of participants | 39 | 17 | 20 | 8 | 84 |
|  |  |  |  |  |  |  |
| Portugal | Proportion | 0·125 | 0·5848 | 0·2321 | 0·058 | 1 |
|  | (95%CI) | [·0545,·2615] | [·3975,·7505] | [·0995,·4528] | [·0175,·1757] |  |
|  | Number of participants | 8 | 29 | 7 | 4 | 48 |
|  |  |  |  |  |  |  |
| Romania | Proportion | 0·1071 | 0·7857 | 0 | 0·1071 | 1 |
|  | (95%CI) | [·0141,·5011] | [·4143,·95] |  | [·0141,·5011] |  |
|  | Number of participants | 1 | 5 | 0 | 1 | 7 |
|  |  |  |  |  |  |  |
| Slovenia | Proportion | 0·1522 | 0·3602 | 0·2547 | 0·2329 | 1 |
|  | (95%CI) | [·0668,·3105] | [·198,·5622] | [·1171,·468] | [·101,·4506] |  |
|  | Number of participants | 6 | 12 | 8 | 7 | 33 |
|  |  |  |  |  |  |  |
| Spain | Proportion | 0·0963 | 0·8182 | 0·0393 | 0·0461 | 1 |
|  | (95%CI) | [·0474,·186] | [·7146,·89] | [·0122,·1193] | [·0188,·1089] |  |
|  | Number of participants | 9 | 53 | 3 | 5 | 70 |
|  |  |  |  |  |  |  |
| Sweden | Proportion | 0·04 | 0·96 | 0 | 0 | 1 |
|  | (95%CI) | [·0049,·2591] | [·7409,·9951] | |  |  |
|  | Number of participants | 1 | 11 | 0 | 0 | 12 |
|  |  |  |  |  |  |  |
| Switzerland | Proportion | 0·1512 | 0·7512 | 0·0976 | 0 | 1 |
|  | (95%CI) | [·0527,·3633] | [·52,·8938] | [·0238,·3237] | |  |
|  | Number of participants | 4 | 16 | 2 | 0 | 22 |
|  |  |  |  |  |  |  |
| Ukraine | Proportion | 0·1213 | 0·6255 | 0·0983 | 0·1548 | 1 |
|  | (95%CI) | [·0465,·2809] | [·4527,·7713] | [·035,·2471] | [·0626,·3342] |  |
|  | Number of participants | 7 | 33 | 7 | 6 | 53 |
|  |  |  |  |  |  |  |
| United K | Proportion | 0·0764 | 0·8701 | 0·0535 | 0 | 1 |
|  | (95%CI) | [·0321,·1711] | [·7697,·9306] | [·0211,·1291] | |  |
|  | Number of participants | 6 | 65 | 5 | 0 | 76 |
|  |  |  |  |  |  |  |
| Total | Proportion | 0·1421 | 0·6233 | 0·1086 | 0·126 | 1 |
|  | (95%CI) | [·1178,·1703] | [·586,·6593] | [·0876,·134] | [·1029,·1534] |  |
|  | Number of participants | 147 | 588 | 108 | 119 | 962 |

| **Country** |  | **RSV** | | | | |
| --- | --- | --- | --- | --- | --- | --- |
|  |  | I would not use the test | I would use both POCT and lab versions of the test | I would use only the lab version of the test | I would use only the POCT version | Total |
| Austria | Proportion | 0·5069 | 0·0803 | 0·1025 | 0·3102 | 1 |
|  | (95%CI) | [·3695,·6433] | [·0284,·2072] | [·0439,·2211] | [·1996,·448] |  |
|  | Number of participants | 37 | 5 | 8 | 25 | 75 |
|  |  |  |  |  |  |  |
| Belgium | Proportion | 0·5057 | 0·041 | 0·0661 | 0·3872 | 1 |
|  | (95%CI) | [·3463,·6639] | [·0101,·1515] | [·0186,·2089] | [·2496,·5456] |  |
|  | Number of participants | 23 | 2 | 3 | 23 | 51 |
|  |  |  |  |  |  |  |
| Bulgaria | Proportion | 0·12 | 0 | 0·12 | 0·76 | 1 |
|  | (95%CI) | [·0143,·5616] | | [·0143,·5616] | [·3343,·9523] |  |
|  | Number of participants | 1 | 0 | 1 | 3 | 5 |
|  |  |  |  |  |  |  |
| Croatia | Proportion | 0·4594 | 0·0106 | 0·1131 | 0·417 | 1 |
|  | (95%CI) | [·2773,·6529] | [·0014,·0736] | [·0358,·3047] | [·2435,·6138] |  |
|  | Number of participants | 12 | 1 | 3 | 13 | 29 |
|  |  |  |  |  |  |  |
| Cyprus | Proportion | 0·3689 | 0·1262 | 0·3883 | 0·1165 | 1 |
|  | (95%CI) | [·0886,·7785] | [·028,·4205] | [·1411,·7105] | [·0257,·397] |  |
|  | Number of participants | 2 | 2 | 6 | 2 | 12 |
|  |  |  |  |  |  |  |
| Czech Rep· | Proportion | 1 | 0 | 0 | 0 | 1 |
|  | (95%CI) |  |  |  |  |  |
|  | Number of participants | 1 | 0 | 0 | 0 | 1 |
|  |  |  |  |  |  |  |
| Denmark | Proportion | 0·2 | 0 | 0 | 0·8 | 1 |
|  | (95%CI) | [·0264,·6975] | |  | [·3025,·9736] |  |
|  | Number of participants | 1 | 0 | 0 | 4 | 5 |
|  |  |  |  |  |  |  |
| Finland | Proportion | 0·4346 | 0·1055 | 0 | 0·4599 | 1 |
|  | (95%CI) | [·2499,·6394] | [·0268,·3356] | | [·2721,·6598] |  |
|  | Number of participants | 10 | 2 | 0 | 12 | 24 |
|  |  |  |  |  |  |  |
| France | Proportion | 0·5117 | 0 | 0·0779 | 0·4104 | 1 |
|  | (95%CI) | [·307,·7125] |  | [·0168,·2943] | [·226,·6239] |  |
|  | Number of participants | 17 | 0 | 2 | 12 | 31 |
|  |  |  |  |  |  |  |
| Germany | Proportion | 0·319 | 0·0184 | 0·2945 | 0·3681 | 1 |
|  | (95%CI) | [·1743,·5097] | [·0025,·1222] | [·1303,·5376] | [·2104,·5601] |  |
|  | Number of participants | 22 | 1 | 8 | 20 | 51 |
|  |  |  |  |  |  |  |
| Greece | Proportion | 0·2823 | 0·0159 | 0·0447 | 0·6571 | 1 |
|  | (95%CI) | [·176,·42] | [·0022,·1057] | [·0151,·1244] | [·5162,·7749] |  |
|  | Number of participants | 23 | 1 | 4 | 36 | 64 |
|  |  |  |  |  |  |  |
| Hungary | Proportion | 0·2966 | 0·2373 | 0·1695 | 0·2966 | 1 |
|  | (95%CI) | [·1204,·565] | [·0644,·5843] | [·0392,·5052] | [·1202,·5656] |  |
|  | Number of participants | 5 | 2 | 2 | 5 | 14 |
|  |  |  |  |  |  |  |
| Ireland | Proportion | 0 | 0 | 0 | 1 | 1 |
|  | (95%CI) |  |  |  |  |  |
|  | Number of participants | 0 | 0 | 0 | 3 | 3 |
|  |  |  |  |  |  |  |
| Israel | Proportion | 0·8421 | 0 | 0 | 0·1579 | 1 |
|  | (95%CI) | [·2492,·9885] | |  | [·0115,·7508] |  |
|  | Number of participants | 1 | 0 | 0 | 1 | 2 |
|  |  |  |  |  |  |  |
| Italy | Proportion | 0·5 | 0 | 0·0973 | 0·4027 | 1 |
|  | (95%CI) | [·2481,·7519] | | [·0182,·3854] | [·1806,·6734] |  |
|  | Number of participants | 14 | 0 | 2 | 11 | 27 |
|  |  |  |  |  |  |  |
| Latvia | Proportion | 0·2 | 0 | 0·2333 | 0·5667 | 1 |
|  | (95%CI) | [·039,·6062] |  | [·035,·7184] | [·2088,·8663] |  |
|  | Number of participants | 2 | 0 | 1 | 6 | 9 |
|  |  |  |  |  |  |  |
| Lithuania | Proportion | 0·8958 | 0 | 0 | 0·1042 | 1 |
|  | (95%CI) | [·6302,·9775] | |  | [·0225,·3698] |  |
|  | Number of participants | 10 | 0 | 0 | 2 | 12 |
|  |  |  |  |  |  |  |
| Malta | Proportion | 0·2049 | 0·0728 | 0·1509 | 0·5714 | 1 |
|  | (95%CI) | [·1004,·373] | [·0235,·2039] | [·0633,·3187] | [·4075,·7211] |  |
|  | Number of participants | 7 | 3 | 5 | 24 | 39 |
|  |  |  |  |  |  |  |
| Netherlands | Proportion | 0·7246 | 0·0388 | 0·0222 | 0·2144 | 1 |
|  | (95%CI) | [·5792,·8341] | [·0121,·1178] | [·0054,·0859] | [·1163,·3615] |  |
|  | Number of participants | 35 | 3 | 2 | 10 | 50 |
|  |  |  |  |  |  |  |
| Norway | Proportion | 0·403 | 0·056 | 0·1847 | 0·3563 | 1 |
|  | (95%CI) | [·2763,·5441] | [·0179,·1619] | [·0938,·3314] | [·2371,·4965] |  |
|  | Number of participants | 22 | 3 | 8 | 20 | 53 |
|  |  |  |  |  |  |  |
| Poland | Proportion | 0·1036 | 0·0084 | 0·0112 | 0·8768 | 1 |
|  | (95%CI) | [·0431,·2291] | [·0021,·0338] | [·0026,·0464] | [·7567,·9421] |  |
|  | Number of participants | 8 | 2 | 2 | 72 | 84 |
|  |  |  |  |  |  |  |
| Portugal | Proportion | 0·7589 | 0 | 0·0491 | 0·192 | 1 |
|  | (95%CI) | [·5983,·8694] | | [·0126,·1733] | [·0969,·3447] |  |
|  | Number of participants | 34 | 0 | 3 | 11 | 48 |
|  |  |  |  |  |  |  |
| Romania | Proportion | 0·3214 | 0 | 0 | 0·6786 | 1 |
|  | (95%CI) | [·0827,·7134] | |  | [·2866,·9173] |  |
|  | Number of participants | 2 | 0 | 0 | 5 | 7 |
|  |  |  |  |  |  |  |
| Slovenia | Proportion | 0·6522 | 0·0497 | 0 | 0·2981 | 1 |
|  | (95%CI) | [·4683,·7997] | [·012,·1836] |  | [·1649,·4775] |  |
|  | Number of participants | 19 | 2 | 0 | 12 | 33 |
|  |  |  |  |  |  |  |
| Spain | Proportion | 0·2877 | 0·0163 | 0·0638 | 0·6323 | 1 |
|  | (95%CI) | [·1877,·4138] | [·004,·0637] | [·0248,·1545] | [·5044,·7439] |  |
|  | Number of participants | 22 | 2 | 5 | 41 | 70 |
|  |  |  |  |  |  |  |
| Sweden | Proportion | 0·59 | 0 | 0·33 | 0·08 | 1 |
|  | (95%CI) | [·2101,·8862] | | [·0673,·7708] | [·0168,·307] |  |
|  | Number of participants | 8 | 0 | 2 | 2 | 12 |
|  |  |  |  |  |  |  |
| Switzerland | Proportion | 0·7415 | 0 | 0 | 0·2585 | 1 |
|  | (95%CI) | [·5201,·8836] | |  | [·1164,·4799] |  |
|  | Number of participants | 14 | 0 | 0 | 8 | 22 |
|  |  |  |  |  |  |  |
| Ukraine | Proportion | 0·2029 | 0·0418 | 0·0167 | 0·7385 | 1 |
|  | (95%CI) | [·1019,·3637] | [·0167,·1009] | [·0041,·0663] | [·5825,·8511] |  |
|  | Number of participants | 11 | 5 | 2 | 35 | 53 |
|  |  |  |  |  |  |  |
| UK | Proportion | 0·2701 | 0·1185 | 0·1758 | 0·4357 | 1 |
|  | (95%CI) | [·1751,·392] | [·0595,·2222] | [·0993,·2921] | [·3212,·5574] |  |
|  | Number of participants | 20 | 9 | 12 | 35 | 76 |
|  |  |  |  |  |  |  |
| Total | Proportion | 0·4001 | 0·0415 | 0·0931 | 0·4653 | 1 |
|  | (95%CI) | [·3632,·4382] | [·03,·0571] | [·0719,·1198] | [·4277,·5033] |  |
|  | Number of participants | 383 | 45 | 81 | 453 | 962 |

| **Country** |  | **Influenza** | | | | |
| --- | --- | --- | --- | --- | --- | --- |
|  |  | I would not use the test | I would use both POCT and lab versions of the test | I would use only the lab version of the test | I would use only the POCT version | Total |
|  |  |  |  |  |  |  |
| Austria | Proportion | 0·4474 | 0·0457 | 0·1787 | 0·3283 | 1 |
|  | (95%CI) | [·3168,·5856] | [·015,·1312] | [·0846,·3386] | [·2115,·471] |  |
|  | Number of participants | 36 | 4 | 11 | 24 | 75 |
|  |  |  |  |  |  |  |
| Belgium | Proportion | 0·4943 | 0·041 | 0·0661 | 0·3986 | 1 |
|  | (95%CI) | [·3353,·6545] | [·0101,·1515] | [·0186,·2089] | [·2585,·5576] |  |
|  | Number of participants | 23 | 2 | 3 | 23 | 51 |
|  |  |  |  |  |  |  |
| Bulgaria | Proportion | 0·12 | 0 | 0·12 | 0·76 | 1 |
|  | (95%CI) | [·0143,·5616] | | [·0143,·5616] | [·3343,·9523] |  |
|  | Number of participants | 1 | 0 | 1 | 3 | 5 |
|  |  |  |  |  |  |  |
| Croatia | Proportion | 0·53 | 0·0106 | 0·0636 | 0·3958 | 1 |
|  | (95%CI) | [·3378,·7137] | [·0014,·0736] | [·0157,·2242] | [·2234,·5986] |  |
|  | Number of participants | 15 | 1 | 2 | 11 | 29 |
|  |  |  |  |  |  |  |
| Cyprus | Proportion | 0·3689 | 0·1262 | 0·3204 | 0·1845 | 1 |
|  | (95%CI) | [·0886,·7785] | [·028,·4205] | [·1108,·6408] | [·0515,·4852] |  |
|  | Number of participants | 2 | 2 | 5 | 3 | 12 |
|  |  |  |  |  |  |  |
| Czech Rep· | Proportion | 1 | 0 | 0 | 0 | 1 |
|  | (95%CI) |  |  |  |  |  |
|  | Number of participants | 1 | 0 | 0 | 0 | 1 |
|  |  |  |  |  |  |  |
| Denmark | Proportion | 0·2 | 0 | 0 | 0·8 | 1 |
|  | (95%CI) | [·0264,·6975] | |  | [·3025,·9736] |  |
|  | Number of participants | 1 | 0 | 0 | 4 | 5 |
|  |  |  |  |  |  |  |
| Finland | Proportion | 0·308 | 0·1055 | 0·0549 | 0·5316 | 1 |
|  | (95%CI) | [·1526,·5239] | [·0268,·3356] | [·0077,·3018] | [·3307,·7229] |  |
|  | Number of participants | 7 | 2 | 1 | 14 | 24 |
|  |  |  |  |  |  |  |
| France | Proportion | 0·5117 | 0 | 0·0571 | 0·4312 | 1 |
|  | (95%CI) | [·307,·7125] |  | [·008,·3119] | [·2426,·6421] |  |
|  | Number of participants | 17 | 0 | 1 | 13 | 31 |
|  |  |  |  |  |  |  |
| Germany | Proportion | 0·4785 | 0·0368 | 0·1227 | 0·362 | 1 |
|  | (95%CI) | [·2928,·6704] | [·0089,·1398] | [·0331,·3638] | [·1962,·5686] |  |
|  | Number of participants | 28 | 2 | 4 | 17 | 51 |
|  |  |  |  |  |  |  |
| Greece | Proportion | 0·1834 | 0·075 | 0·0064 | 0·7352 | 1 |
|  | (95%CI) | [·1024,·3067] | [·0236,·2137] | [8·8e-04,·0447] | [·5949,·84] |  |
|  | Number of participants | 17 | 3 | 1 | 43 | 64 |
|  |  |  |  |  |  |  |
| Hungary | Proportion | 0·2288 | 0·2373 | 0·1695 | 0·3644 | 1 |
|  | (95%CI) | [·0832,·4925] | [·0644,·5843] | [·0392,·5052] | [·1605,·6322] |  |
|  | Number of participants | 4 | 2 | 2 | 6 | 14 |
|  |  |  |  |  |  |  |
| Ireland | Proportion | 0 | 0 | 0 | 1 | 1 |
|  | (95%CI) |  |  |  |  |  |
|  | Number of participants | 0 | 0 | 0 | 3 | 3 |
|  |  |  |  |  |  |  |
| Israel | Proportion | 0·8421 | 0 | 0 | 0·1579 | 1 |
|  | (95%CI) | [·2492,·9885] | |  | [·0115,·7508] |  |
|  | Number of participants | 1 | 0 | 0 | 1 | 2 |
|  |  |  |  |  |  |  |
| Italy | Proportion | 0·5034 | 0 | 0·0805 | 0·4161 | 1 |
|  | (95%CI) | [·2507,·7543] | | [·0112,·4041] | [·1902,·6837] |  |
|  | Number of participants | 14 | 0 | 1 | 12 | 27 |
|  |  |  |  |  |  |  |
| Latvia | Proportion | 0 | 0 | 0·2333 | 0·7667 | 1 |
|  | (95%CI) |  |  | [·035,·7184] | [·2816,·965] |  |
|  | Number of participants | 0 | 0 | 1 | 8 | 9 |
|  |  |  |  |  |  |  |
| Lithuania | Proportion | 0·7396 | 0·0521 | 0·1042 | 0·1042 | 1 |
|  | (95%CI) | [·4325,·9137] | [·0066,·3136] | [·0225,·3698] | [·0225,·3698] |  |
|  | Number of participants | 7 | 1 | 2 | 2 | 12 |
|  |  |  |  |  |  |  |
| Malta | Proportion | 0·2237 | 0·0728 | 0·1509 | 0·5526 | 1 |
|  | (95%CI) | [·1147,·3906] | [·0235,·2039] | [·0633,·3187] | [·3906,·7041] |  |
|  | Number of participants | 8 | 3 | 5 | 23 | 39 |
|  |  |  |  |  |  |  |
| Netherlands | Proportion | 0·6691 | 0·0388 | 0·0222 | 0·2699 | 1 |
|  | (95%CI) | [·5196,·7909] | [·0121,·1178] | [·0054,·0859] | [·1574,·4223] |  |
|  | Number of participants | 33 | 3 | 2 | 12 | 50 |
|  |  |  |  |  |  |  |
| Norway | Proportion | 0·4683 | 0·0354 | 0·1903 | 0·306 | 1 |
|  | (95%CI) | [·3341,·6073] | [·0087,·1336] | [·1001,·3318] | [·1948,·4455] |  |
|  | Number of participants | 25 | 2 | 9 | 17 | 53 |
|  |  |  |  |  |  |  |
| Poland | Proportion | 0·0084 | 0·0084 | 0·0182 | 0·965 | 1 |
|  | (95%CI) | [·0021,·0338] | [·0021,·0338] | [·0056,·0577] | [·9241,·9842] |  |
|  | Number of participants | 2 | 2 | 3 | 77 | 84 |
|  |  |  |  |  |  |  |
| Portugal | Proportion | 0·75 | 0 | 0·0402 | 0·2098 | 1 |
|  | (95%CI) | [·5878,·8632] | | [·0081,·1764] | [·1095,·3645] |  |
|  | Number of participants | 34 | 0 | 2 | 12 | 48 |
|  |  |  |  |  |  |  |
| Romania | Proportion | 0·3214 | 0 | 0 | 0·6786 | 1 |
|  | (95%CI) | [·0827,·7134] | |  | [·2866,·9173] |  |
|  | Number of participants | 2 | 0 | 0 | 5 | 7 |
|  |  |  |  |  |  |  |
| Slovenia | Proportion | 0·4224 | 0·0217 | 0·0217 | 0·5342 | 1 |
|  | (95%CI) | [·2417,·6264] | [·003,·1416] | [·003,·1416] | [·3387,·7197] |  |
|  | Number of participants | 12 | 1 | 1 | 19 | 33 |
|  |  |  |  |  |  |  |
| Spain | Proportion | 0·1696 | 0·0081 | 0·1153 | 0·7069 | 1 |
|  | (95%CI) | [·0964,·2812] | [·0011,·0561] | [·0556,·2239] | [·5821,·8069] |  |
|  | Number of participants | 14 | 1 | 8 | 47 | 70 |
|  |  |  |  |  |  |  |
| Sweden | Proportion | 0·63 | 0 | 0·33 | 0·04 | 1 |
|  | (95%CI) | [·2222,·9103] | | [·0673,·7708] | [·0049,·2591] |  |
|  | Number of participants | 9 | 0 | 2 | 1 | 12 |
|  |  |  |  |  |  |  |
| Switzerland | Proportion | 0·6049 | 0 | 0 | 0·3951 | 1 |
|  | (95%CI) | [·3708,·7991] | |  | [·2009,·6292] |  |
|  | Number of participants | 11 | 0 | 0 | 11 | 22 |
|  |  |  |  |  |  |  |
| Ukraine | Proportion | 0·1569 | 0·0502 | 0·0251 | 0·7678 | 1 |
|  | (95%CI) | [·0735,·3041] | [·0193,·1244] | [·0078,·0776] | [·6225,·8689] |  |
|  | Number of participants | 10 | 5 | 3 | 35 | 53 |
|  |  |  |  |  |  |  |
| UK | Proportion | 0·2892 | 0·1108 | 0·1261 | 0·4739 | 1 |
|  | (95%CI) | [·192,·4105] | [·0536,·2152] | [·0642,·2329] | [·3559,·5948] |  |
|  | Number of participants | 22 | 8 | 9 | 37 | 76 |
|  |  |  |  |  |  |  |
| Total | Proportion | 0·3642 | 0·0414 | 0·0875 | 0·5069 | 1 |
|  | (95%CI) | [·3282,·4019] | [·0299,·057] | [·0676,·1126] | [·4688,·5449] |  |
|  | Number of participants | 356 | 44 | 79 | 483 | 962 |

| **Country** |  | **CRP** | | | | |
| --- | --- | --- | --- | --- | --- | --- |
|  |  | I would not use the test | I would use both POCT and lab versions of the test | I would use only the lab version of the test | I would use only the POCT version | Total |
|  |  |  |  |  |  |  |
| Austria | Proportion | 0·1219 | 0·0762 | 0·205 | 0·597 | 1 |
|  | (95%CI) | [·0618,·2262] | [·0322,·1696] | [·1057,·36] | [·4543,·7249] |  |
|  | Number of participants | 11 | 7 | 14 | 43 | 75 |
|  |  |  |  |  |  |  |
| Belgium | Proportion | 0·1913 | 0·0501 | 0·2528 | 0·5057 | 1 |
|  | (95%CI) | [·1031,·3275] | [·0151,·154] | [·1175,·4623] | [·3456,·6647] |  |
|  | Number of participants | 13 | 3 | 8 | 27 | 51 |
|  |  |  |  |  |  |  |
| Bulgaria | Proportion | 0 | 0 | 0·56 | 0·44 | 1 |
|  | (95%CI) |  |  | [·1489,·9025] | [·0975,·8511] |  |
|  | Number of participants | 0 | 0 | 3 | 2 | 5 |
|  |  |  |  |  |  |  |
| Croatia | Proportion | 0·1696 | 0·1095 | 0·583 | 0·1378 | 1 |
|  | (95%CI) | [·063,·3831] | [·0314,·3184] | [·3862,·7565] | [·0575,·2952] |  |
|  | Number of participants | 4 | 3 | 16 | 6 | 29 |
|  |  |  |  |  |  |  |
| Cyprus | Proportion | 0 | 0·0583 | 0·3883 | 0·5534 | 1 |
|  | (95%CI) |  | [·0074,·3376] | [·1411,·7105] | [·2344,·8338] |  |
|  | Number of participants | 0 | 1 | 6 | 5 | 12 |
|  |  |  |  |  |  |  |
| Czech Rep· | Proportion | 0 | 0 | 1 | 0 | 1 |
|  | (95%CI) |  |  |  |  |  |
|  | Number of participants | 0 | 0 | 1 | 0 | 1 |
|  |  |  |  |  |  |  |
| Denmark | Proportion | 0 | 0·2 | 0·2857 | 0·5143 | 1 |
|  | (95%CI) |  | [·0264,·6975] | [·0614,·7098] | [·1454,·8682] |  |
|  | Number of participants | 0 | 1 | 2 | 2 | 5 |
|  |  |  |  |  |  |  |
| Finland | Proportion | 0 | 0·0506 | 0·3882 | 0·5612 | 1 |
|  | (95%CI) |  | [·0071,·2843] | [·2129,·5981] | [·3566,·7469] |  |
|  | Number of participants | 0 | 1 | 9 | 14 | 24 |
|  |  |  |  |  |  |  |
| France | Proportion | 0·3143 | 0 | 0·1429 | 0·5429 | 1 |
|  | (95%CI) | [·1682,·5095] | | [·0459,·3659] | [·3376,·7345] |  |
|  | Number of participants | 15 | 0 | 4 | 12 | 31 |
|  |  |  |  |  |  |  |
| Germany | Proportion | 0·2086 | 0·0491 | 0·2577 | 0·4847 | 1 |
|  | (95%CI) | [·0902,·412] | [·0157,·143] | [·1124,·4874] | [·2978,·6759] |  |
|  | Number of participants | 10 | 4 | 10 | 27 | 51 |
|  |  |  |  |  |  |  |
| Greece | Proportion | 0·1021 | 0·2201 | 0·3142 | 0·3636 | 1 |
|  | (95%CI) | [·0507,·1949] | [·1227,·3629] | [·1975,·4603] | [·2286,·5243] |  |
|  | Number of participants | 10 | 13 | 22 | 19 | 64 |
|  |  |  |  |  |  |  |
| Hungary | Proportion | 0 | 0·1186 | 0·4831 | 0·3983 | 1 |
|  | (95%CI) |  | [·0171,·51] | [·2339,·7409] | [·1833,·6612] |  |
|  | Number of participants | 0 | 1 | 6 | 7 | 14 |
|  |  |  |  |  |  |  |
| Ireland | Proportion | 0·4667 | 0 | 0 | 0·5333 | 1 |
|  | (95%CI) | [·0695,·9111] | |  | [·0889,·9305] |  |
|  | Number of participants | 1 | 0 | 0 | 2 | 3 |
|  |  |  |  |  |  |  |
| Israel | Proportion | 0 | 0 | 0·1579 | 0·8421 | 1 |
|  | (95%CI) |  |  | [·0115,·7508] | [·2492,·9885] |  |
|  | Number of participants | 0 | 0 | 1 | 1 | 2 |
|  |  |  |  |  |  |  |
| Italy | Proportion | 0·1644 | 0·1074 | 0·2919 | 0·4362 | 1 |
|  | (95%CI) | [·0329,·5326] | [·0231,·3793] | [·1098,·5795] | [·2041,·7001] |  |
|  | Number of participants | 3 | 3 | 9 | 12 | 27 |
|  |  |  |  |  |  |  |
| Latvia | Proportion | 0·2889 | 0 | 0·1 | 0·6111 | 1 |
|  | (95%CI) | [·0603,·7202] | | [·0212,·3628] | [·2408,·8861] |  |
|  | Number of participants | 2 | 0 | 2 | 5 | 9 |
|  |  |  |  |  |  |  |
| Lithuania | Proportion | 0·5313 | 0 | 0·2604 | 0·2083 | 1 |
|  | (95%CI) | [·2079,·8303] | | [·0863,·5675] | [·064,·5033] |  |
|  | Number of participants | 3 | 0 | 5 | 4 | 12 |
|  |  |  |  |  |  |  |
| Malta | Proportion | 0·1752 | 0·2022 | 0·1402 | 0·4825 | 1 |
|  | (95%CI) | [·0844,·3286] | [·1026,·3596] | [·0582,·3007] | [·3279,·6405] |  |
|  | Number of participants | 7 | 8 | 5 | 19 | 39 |
|  |  |  |  |  |  |  |
| Netherlands | Proportion | 0·1294 | 0·0425 | 0·2847 | 0·5434 | 1 |
|  | (95%CI) | [·0569,·268] | [·0103,·1595] | [·1702,·4357] | [·3958,·6838] |  |
|  | Number of participants | 6 | 2 | 14 | 28 | 50 |
|  |  |  |  |  |  |  |
| Norway | Proportion | 0 | 0·0802 | 0·4907 | 0·4291 | 1 |
|  | (95%CI) |  | [·0331,·1817] | [·3544,·6283] | [·299,·5698] |  |
|  | Number of participants | 0 | 5 | 25 | 23 | 53 |
|  |  |  |  |  |  |  |
| Poland | Proportion | 0·0042 | 0·1303 | 0·049 | 0·8165 | 1 |
|  | (95%CI) | [5·8e-04,·0297] | [·059,·2634] | [·0153,·1457] | [·6824,·9021] |  |
|  | Number of participants | 1 | 9 | 5 | 69 | 84 |
|  |  |  |  |  |  |  |
| Portugal | Proportion | 0·3259 | 0·058 | 0·2589 | 0·3571 | 1 |
|  | (95%CI) | [·1902,·4988] | [·0163,·1862] | [·1405,·4274] | [·1904,·5676] |  |
|  | Number of participants | 18 | 3 | 14 | 13 | 48 |
|  |  |  |  |  |  |  |
| Romania | Proportion | 0·1786 | 0 | 0·25 | 0·5714 | 1 |
|  | (95%CI) | [·0253,·6455] | | [·0595,·6372] | [·2245,·8599] |  |
|  | Number of participants | 1 | 0 | 2 | 4 | 7 |
|  |  |  |  |  |  |  |
| Slovenia | Proportion | 0 | 0·0745 | 0·3758 | 0·5497 | 1 |
|  | (95%CI) |  | [·0234,·2134] | [·2108,·5756] | [·3561,·7293] |  |
|  | Number of participants | 0 | 3 | 13 | 17 | 33 |
|  |  |  |  |  |  |  |
| Spain | Proportion | 0·4396 | 0·0163 | 0·2374 | 0·3066 | 1 |
|  | (95%CI) | [·3192,·5676] | [·004,·0637] | [·1404,·3726] | [·2,·439] |  |
|  | Number of participants | 35 | 2 | 13 | 20 | 70 |
|  |  |  |  |  |  |  |
| Sweden | Proportion | 0·12 | 0 | 0 | 0·88 | 1 |
|  | (95%CI) | [·0314,·3649] | |  | [·6351,·9686] |  |
|  | Number of participants | 3 | 0 | 0 | 9 | 12 |
|  |  |  |  |  |  |  |
| Switzerland | Proportion | 0·2537 | 0·0439 | 0·239 | 0·4634 | 1 |
|  | (95%CI) | [·1086,·4868] | [·006,·2591] | [·0773,·5407] | [·2402,·7023] |  |
|  | Number of participants | 6 | 1 | 4 | 11 | 22 |
|  |  |  |  |  |  |  |
| Ukraine | Proportion | 0·228 | 0·0669 | 0·1213 | 0·5837 | 1 |
|  | (95%CI) | [·1171,·3969] | [·0297,·144] | [·0458,·2844] | [·4179,·7325] |  |
|  | Number of participants | 13 | 7 | 6 | 27 | 53 |
|  |  |  |  |  |  |  |
| UK | Proportion | 0·065 | 0·149 | 0·321 | 0·465 | 1 |
|  | (95%CI) | [·0273,·147] | [·0774,·2677] | [·22,·4421] | [·3476,·5863] |  |
|  | Number of participants | 6 | 9 | 26 | 35 | 76 |
|  |  |  |  |  |  |  |
| Total | Proportion | 0·1583 | 0·0852 | 0·2585 | 0·498 | 1 |
|  | (95%CI) | [·1334,·1869] | [·0673,·1074] | [·2265,·2932] | [·46,·536] |  |
|  | Number of participants | 168 | 86 | 245 | 463 | 962 |

| **Country** |  | **Procalcitonin** | | | | |
| --- | --- | --- | --- | --- | --- | --- |
|  |  | I would not use the test | I would use both POCT and lab versions of the test | I would use only the lab version of the test | I would use only the POCT version | Total |
|  |  |  |  |  |  |  |
| Austria | Proportion | 0·6316 | 0·0346 | 0·1856 | 0·1482 | 1 |
|  | (95%CI) | [·4766,·7634] | [·0049,·2068] | [·0854,·3573] | [·072,·2808] |  |
|  | Number of participants | 58 | 1 | 8 | 8 | 75 |
|  |  |  |  |  |  |  |
| Belgium | Proportion | 0·7836 | 0 | 0·1321 | 0·0843 | 1 |
|  | (95%CI) | [·5667,·9093] | | [·0343,·3947] | [·0287,·223] |  |
|  | Number of participants | 44 | 0 | 3 | 4 | 51 |
|  |  |  |  |  |  |  |
| Bulgaria | Proportion | 0·44 | 0 | 0·12 | 0·44 | 1 |
|  | (95%CI) | [·0975,·8511] | | [·0143,·5616] | [·0975,·8511] |  |
|  | Number of participants | 2 | 0 | 1 | 2 | 5 |
|  |  |  |  |  |  |  |
| Croatia | Proportion | 0·3887 | 0·0601 | 0·3675 | 0·1837 | 1 |
|  | (95%CI) | [·226,·5807] | [·0115,·2597] | [·1995,·5753] | [·0724,·3936] |  |
|  | Number of participants | 13 | 2 | 9 | 5 | 29 |
|  |  |  |  |  |  |  |
| Cyprus | Proportion | 0·4951 | 0 | 0·3204 | 0·1845 | 1 |
|  | (95%CI) | [·183,·8112] |  | [·1108,·6408] | [·0515,·4852] |  |
|  | Number of participants | 4 | 0 | 5 | 3 | 12 |
|  |  |  |  |  |  |  |
| Czech Rep· | Proportion | 0 | 0 | 1 | 0 | 1 |
|  | (95%CI) |  |  |  |  |  |
|  | Number of participants | 0 | 0 | 1 | 0 | 1 |
|  |  |  |  |  |  |  |
| Denmark | Proportion | 0·6857 | 0·3143 | 0 | 0 | 1 |
|  | (95%CI) | [·1945,·9517] | [·0483,·8055] | |  |  |
|  | Number of participants | 4 | 1 | 0 | 0 | 5 |
|  |  |  |  |  |  |  |
| Finland | Proportion | 0·8228 | 0 | 0·0506 | 0·1266 | 1 |
|  | (95%CI) | [·6067,·9332] | | [·0071,·2843] | [·04,·3354] |  |
|  | Number of participants | 20 | 0 | 1 | 3 | 24 |
|  |  |  |  |  |  |  |
| France | Proportion | 0·5558 | 0 | 0·1351 | 0·3091 | 1 |
|  | (95%CI) | [·3409,·7517] | | [·0409,·3637] | [·1423,·5468] |  |
|  | Number of participants | 20 | 0 | 3 | 8 | 31 |
|  |  |  |  |  |  |  |
| Germany | Proportion | 0·7546 | 0·0368 | 0·0982 | 0·1104 | 1 |
|  | (95%CI) | [·5562,·883] | [·0089,·1398] | [·0198,·3702] | [·0493,·2293] |  |
|  | Number of participants | 39 | 2 | 2 | 8 | 51 |
|  |  |  |  |  |  |  |
| Greece | Proportion | 0·4386 | 0·1244 | 0·1898 | 0·2472 | 1 |
|  | (95%CI) | [·3039,·583] | [·0534,·2636] | [·0972,·3377] | [·1295,·4203] |  |
|  | Number of participants | 38 | 6 | 9 | 11 | 64 |
|  |  |  |  |  |  |  |
| Hungary | Proportion | 0·0593 | 0·1186 | 0·3559 | 0·4661 | 1 |
|  | (95%CI) | [·0081,·3281] | [·0171,·51] | [·1477,·638] | [·2233,·7261] |  |
|  | Number of participants | 1 | 1 | 5 | 7 | 14 |
|  |  |  |  |  |  |  |
| Ireland | Proportion | 0·4667 | 0 | 0·3667 | 0·1667 | 1 |
|  | (95%CI) | [·0695,·9111] | | [·0457,·8751] | [·0176,·6902] |  |
|  | Number of participants | 1 | 0 | 1 | 1 | 3 |
|  |  |  |  |  |  |  |
| Israel | Proportion | 1 | 0 | 0 | 0 | 1 |
|  | (95%CI) |  |  |  |  |  |
|  | Number of participants | 2 | 0 | 0 | 0 | 2 |
|  |  |  |  |  |  |  |
| Italy | Proportion | 0·3658 | 0·0268 | 0·1745 | 0·4329 | 1 |
|  | (95%CI) | [·1434,·6651] | [·0061,·11] | [·0589,·4167] | [·2018,·6974] |  |
|  | Number of participants | 7 | 2 | 6 | 12 | 27 |
|  |  |  |  |  |  |  |
| Latvia | Proportion | 0·9 | 0 | 0 | 0·1 | 1 |
|  | (95%CI) | [·6372,·9788] | |  | [·0212,·3628] |  |
|  | Number of participants | 7 | 0 | 0 | 2 | 9 |
|  |  |  |  |  |  |  |
| Lithuania | Proportion | 0·6667 | 0·0521 | 0·1771 | 0·1042 | 1 |
|  | (95%CI) | [·3032,·9019] | [·0066,·3136] | [·025,·6438] | [·0225,·3698] |  |
|  | Number of participants | 8 | 1 | 1 | 2 | 12 |
|  |  |  |  |  |  |  |
| Malta | Proportion | 0·3342 | 0·1105 | 0·1078 | 0·4474 | 1 |
|  | (95%CI) | [·2013,·5] | [·0413,·2639] | [·0404,·2573] | [·2974,·6077] |  |
|  | Number of participants | 13 | 4 | 4 | 18 | 39 |
|  |  |  |  |  |  |  |
| Netherlands | Proportion | 0·7135 | 0·0407 | 0 | 0·2458 | 1 |
|  | (95%CI) | [·5628,·8281] | [·0089,·1671] | | [·1404,·3942] |  |
|  | Number of participants | 36 | 2 | 0 | 12 | 50 |
|  |  |  |  |  |  |  |
| Norway | Proportion | 0·6213 | 0·0653 | 0·166 | 0·1474 | 1 |
|  | (95%CI) | [·4789,·7454] | [·0203,·1903] | [·0865,·2951] | [·073,·2751] |  |
|  | Number of participants | 33 | 3 | 9 | 8 | 53 |
|  |  |  |  |  |  |  |
| Poland | Proportion | 0·0714 | 0·0728 | 0·0546 | 0·8011 | 1 |
|  | (95%CI) | [·0246,·1898] | [·0257,·1893] | [·0192,·1459] | [·6688,·8893] |  |
|  | Number of participants | 5 | 6 | 7 | 66 | 84 |
|  |  |  |  |  |  |  |
| Portugal | Proportion | 0·625 | 0·0402 | 0·125 | 0·2098 | 1 |
|  | (95%CI) | [·4519,·7711] | [·0081,·1764] | [·0545,·2615] | [·1095,·3645] |  |
|  | Number of participants | 26 | 2 | 8 | 12 | 48 |
|  |  |  |  |  |  |  |
| Romania | Proportion | 1 | 0 | 0 | 0 | 1 |
|  | (95%CI) |  |  |  |  |  |
|  | Number of participants | 7 | 0 | 0 | 0 | 7 |
|  |  |  |  |  |  |  |
| Slovenia | Proportion | 0·6677 | 0·0217 | 0·1615 | 0·1491 | 1 |
|  | (95%CI) | [·4867,·8098] | [·003,·1416] | [·0745,·3154] | [·0653,·3051] |  |
|  | Number of participants | 19 | 1 | 7 | 6 | 33 |
|  |  |  |  |  |  |  |
| Spain | Proportion | 0·4437 | 0·0081 | 0·2469 | 0·3012 | 1 |
|  | (95%CI) | [·3234,·571] | [·0011,·0561] | [·1476,·3831] | [·1945,·4349] |  |
|  | Number of participants | 37 | 1 | 13 | 19 | 70 |
|  |  |  |  |  |  |  |
| Sweden | Proportion | 0·41 | 0 | 0·12 | 0·47 | 1 |
|  | (95%CI) | [·1281,·7667] | | [·0314,·3649] | [·1495,·8173] |  |
|  | Number of participants | 6 | 0 | 3 | 3 | 12 |
|  |  |  |  |  |  |  |
| Switzerland | Proportion | 0·6439 | 0 | 0·0634 | 0·2927 | 1 |
|  | (95%CI) | [·3801,·8421] | | [·0138,·2469] | [·112,·5759] |  |
|  | Number of participants | 15 | 0 | 2 | 5 | 22 |
|  |  |  |  |  |  |  |
| Ukraine | Proportion | 0·4686 | 0·0418 | 0·0816 | 0·4079 | 1 |
|  | (95%CI) | [·31,·6339] | [·0167,·1009] | [·0235,·2468] | [·2627,·5713] |  |
|  | Number of participants | 22 | 5 | 4 | 22 | 53 |
|  |  |  |  |  |  |  |
| UK | Proportion | 0·6178 | 0·0611 | 0·0535 | 0·2675 | 1 |
|  | (95%CI) | [·4942,·7279] | [·0218,·1597] | [·0195,·1381] | [·1736,·3884] |  |
|  | Number of participants | 47 | 4 | 5 | 20 | 76 |
|  |  |  |  |  |  |  |
| Total | Proportion | 0·531 | 0·046 | 0·1351 | 0·2879 | 1 |
|  | (95%CI) | [·4928,·5689] | [·0328,·0641] | [·1101,·1647] | [·2544,·3239] |  |
|  | Number of participants | 534 | 44 | 117 | 267 | 962 |

| **Country** |  | **Full blood count** | | | | |
| --- | --- | --- | --- | --- | --- | --- |
|  |  | I would not use the test | I would use both POCT and lab versions of the test | I would use only the lab version of the test | I would use only the POCT version | Total |
|  |  |  |  |  |  |  |
| Austria | Proportion | 0·1274 | 0·0762 | 0·277 | 0·5194 | 1 |
|  | (95%CI) | [·0661,·2314] | [·0322,·1696] | [·1654,·4255] | [·3812,·6547] |  |
|  | Number of participants | 12 | 7 | 21 | 35 | 75 |
|  |  |  |  |  |  |  |
| Belgium | Proportion | 0·2005 | 0·041 | 0·2961 | 0·4624 | 1 |
|  | (95%CI) | [·1091,·3392] | [·0101,·1515] | [·1546,·4919] | [·3094,·6228] |  |
|  | Number of participants | 13 | 2 | 12 | 24 | 51 |
|  |  |  |  |  |  |  |
| Bulgaria | Proportion | 0 | 0·12 | 0·32 | 0·56 | 1 |
|  | (95%CI) |  | [·0143,·5616] | [·0472,·8172] | [·1489,·9025] |  |
|  | Number of participants | 0 | 1 | 1 | 3 | 5 |
|  |  |  |  |  |  |  |
| Croatia | Proportion | 0·0318 | 0·1201 | 0·4735 | 0·3746 | 1 |
|  | (95%CI) | [·0044,·196] | [·0379,·3211] | [·2902,·6642] | [·2071,·5785] |  |
|  | Number of participants | 1 | 4 | 14 | 10 | 29 |
|  |  |  |  |  |  |  |
| Cyprus | Proportion | 0 | 0 | 0·8738 | 0·1262 | 1 |
|  | (95%CI) |  |  | [·5795,·972] | [·028,·4205] |  |
|  | Number of participants | 0 | 0 | 10 | 2 | 12 |
|  |  |  |  |  |  |  |
| Czech Rep· | Proportion | 0 | 0 | 1 | 0 | 1 |
|  | (95%CI) |  |  |  |  |  |
|  | Number of participants | 0 | 0 | 1 | 0 | 1 |
|  |  |  |  |  |  |  |
| Denmark | Proportion | 0 | 0 | 0·8 | 0·2 | 1 |
|  | (95%CI) |  |  | [·3025,·9736] | [·0264,·6975] |  |
|  | Number of participants | 0 | 0 | 4 | 1 | 5 |
|  |  |  |  |  |  |  |
| Finland | Proportion | 0·0802 | 0·0802 | 0·519 | 0·3207 | 1 |
|  | (95%CI) | [·0191,·2807] | [·0191,·2807] | [·3211,·7111] | [·1646,·5307] |  |
|  | Number of participants | 2 | 2 | 12 | 8 | 24 |
|  |  |  |  |  |  |  |
| France | Proportion | 0·5792 | 0 | 0·2571 | 0·1636 | 1 |
|  | (95%CI) | [·365,·7672] |  | [·1114,·4887] | [·0612,·3698] |  |
|  | Number of participants | 20 | 0 | 6 | 5 | 31 |
|  |  |  |  |  |  |  |
| Germany | Proportion | 0·2086 | 0·0491 | 0·4601 | 0·2822 | 1 |
|  | (95%CI) | [·0902,·412] | [·0157,·143] | [·2744,·6576] | [·1459,·475] |  |
|  | Number of participants | 10 | 4 | 19 | 18 | 51 |
|  |  |  |  |  |  |  |
| Greece | Proportion | 0·1021 | 0·2265 | 0·4577 | 0·2137 | 1 |
|  | (95%CI) | [·0507,·1949] | [·1253,·3743] | [·3175,·6051] | [·1069,·3817] |  |
|  | Number of participants | 10 | 12 | 29 | 13 | 64 |
|  |  |  |  |  |  |  |
| Hungary | Proportion | 0·0593 | 0 | 0·7034 | 0·2373 | 1 |
|  | (95%CI) | [·0081,·3281] | | [·435,·8796] | [·0866,·5052] |  |
|  | Number of participants | 1 | 0 | 9 | 4 | 14 |
|  |  |  |  |  |  |  |
| Ireland | Proportion | 0·4667 | 0 | 0·3667 | 0·1667 | 1 |
|  | (95%CI) | [·0695,·9111] | | [·0457,·8751] | [·0176,·6902] |  |
|  | Number of participants | 1 | 0 | 1 | 1 | 3 |
|  |  |  |  |  |  |  |
| Israel | Proportion | 0 | 0 | 0·1579 | 0·8421 | 1 |
|  | (95%CI) |  |  | [·0115,·7508] | [·2492,·9885] |  |
|  | Number of participants | 0 | 0 | 1 | 1 | 2 |
|  |  |  |  |  |  |  |
| Italy | Proportion | 0·2785 | 0 | 0·4329 | 0·2886 | 1 |
|  | (95%CI) | [·094,·5897] |  | [·2021,·697] | [·1076,·5771] |  |
|  | Number of participants | 5 | 0 | 13 | 9 | 27 |
|  |  |  |  |  |  |  |
| Latvia | Proportion | 0·2889 | 0 | 0·3 | 0·4111 | 1 |
|  | (95%CI) | [·0603,·7202] | | [·0744,·6957] | [·1306,·7644] |  |
|  | Number of participants | 2 | 0 | 3 | 4 | 9 |
|  |  |  |  |  |  |  |
| Lithuania | Proportion | 0·0521 | 0 | 0·7396 | 0·2083 | 1 |
|  | (95%CI) | [·0066,·3136] | | [·4325,·9137] | [·064,·5033] |  |
|  | Number of participants | 1 | 0 | 7 | 4 | 12 |
|  |  |  |  |  |  |  |
| Malta | Proportion | 0·1509 | 0·1779 | 0·3181 | 0·3531 | 1 |
|  | (95%CI) | [·0681,·3019] | [·0855,·3336] | [·1874,·4854] | [·2176,·5172] |  |
|  | Number of participants | 6 | 7 | 12 | 14 | 39 |
|  |  |  |  |  |  |  |
| Netherlands | Proportion | 0·488 | 0·0166 | 0·3216 | 0·1738 | 1 |
|  | (95%CI) | [·3448,·6332] | [·0023,·1098] | [·2003,·473] | [·0889,·3118] |  |
|  | Number of participants | 24 | 1 | 16 | 9 | 50 |
|  |  |  |  |  |  |  |
| Norway | Proportion | 0·0448 | 0·0802 | 0·5672 | 0·3078 | 1 |
|  | (95%CI) | [·0143,·1312] | [·0331,·1817] | [·4283,·6962] | [·1968,·4467] |  |
|  | Number of participants | 3 | 5 | 28 | 17 | 53 |
|  |  |  |  |  |  |  |
| Poland | Proportion | 0·0042 | 0·0462 | 0·1765 | 0·7731 | 1 |
|  | (95%CI) | [5·8e-04,·0297] | [·0138,·1437] | [·1007,·2907] | [·6509,·8616] |  |
|  | Number of participants | 1 | 5 | 22 | 56 | 84 |
|  |  |  |  |  |  |  |
| Portugal | Proportion | 0·3259 | 0·058 | 0·3259 | 0·2902 | 1 |
|  | (95%CI) | [·1902,·4988] | [·0163,·1862] | [·1751,·5241] | [·1469,·4925] |  |
|  | Number of participants | 18 | 3 | 15 | 12 | 48 |
|  |  |  |  |  |  |  |
| Romania | Proportion | 0·1786 | 0 | 0·1071 | 0·7143 | 1 |
|  | (95%CI) | [·0253,·6455] | | [·0141,·5011] | [·3168,·9309] |  |
|  | Number of participants | 1 | 0 | 1 | 5 | 7 |
|  |  |  |  |  |  |  |
| Slovenia | Proportion | 0·087 | 0·028 | 0·6366 | 0·2484 | 1 |
|  | (95%CI) | [·0127,·4136] | [·0039,·1759] | [·4348,·7996] | [·1303,·4219] |  |
|  | Number of participants | 1 | 1 | 21 | 10 | 33 |
|  |  |  |  |  |  |  |
| Spain | Proportion | 0·4464 | 0·0231 | 0·3392 | 0·1913 | 1 |
|  | (95%CI) | [·3259,·5736] | [·0032,·1461] | [·2262,·4741] | [·1057,·3215] |  |
|  | Number of participants | 38 | 1 | 20 | 11 | 70 |
|  |  |  |  |  |  |  |
| Sweden | Proportion | 0·28 | 0 | 0·51 | 0·21 | 1 |
|  | (95%CI) | [·0964,·5864] | | [·1777,·8337] | [·0303,·6934] |  |
|  | Number of participants | 7 | 0 | 4 | 1 | 12 |
|  |  |  |  |  |  |  |
| Switzerland | Proportion | 0·3902 | 0·1073 | 0·3024 | 0·2 | 1 |
|  | (95%CI) | [·1824,·6475] | [·0315,·3079] | [·1202,·5791] | [·0806,·4164] |  |
|  | Number of participants | 7 | 3 | 6 | 6 | 22 |
|  |  |  |  |  |  |  |
| Ukraine | Proportion | 0·0816 | 0·2448 | 0·4623 | 0·2113 | 1 |
|  | (95%CI) | [·0228,·2525] | [·1301,·4126] | [·306,·6265] | [·1085,·371] |  |
|  | Number of participants | 3 | 14 | 24 | 12 | 53 |
|  |  |  |  |  |  |  |
| UK | Proportion | 0·1185 | 0·1949 | 0·3516 | 0·335 | 1 |
|  | (95%CI) | [·0606,·2186] | [·1133,·3145] | [·2454,·4748] | [·2318,·4569] |  |
|  | Number of participants | 10 | 13 | 27 | 26 | 76 |
|  |  |  |  |  |  |  |
| Total | Proportion | 0·1934 | 0·0844 | 0·3862 | 0·336 | 1 |
|  | (95%CI) | [·1655,·2247] | [·0665,·1064] | [·3495,·4243] | [·301,·3728] |  |
|  | Number of participants | 197 | 85 | 359 | 321 | 962 |

| **Country** |  | **Blood gas analysis (with or without lactate)** | | | | |
| --- | --- | --- | --- | --- | --- | --- |
|  |  | I would not use the test | I would use both POCT and lab versions of the test | I would use only the lab version of the test | I would use only the POCT version | Total |
|  |  |  |  |  |  |  |
| Austria | Proportion | 0·331 | 0·0249 | 0·169 | 0·4751 | 1 |
|  | (95%CI) | [·2127,·4755] | [·0051,·1138] | [·0807,·32] | [·3409,·613] |  |
|  | Number of participants | 25 | 2 | 12 | 36 | 75 |
|  |  |  |  |  |  |  |
| Belgium | Proportion | 0·7267 | 0·0205 | 0·0569 | 0·1959 | 1 |
|  | (95%CI) | [·5703,·8419] | [·0028,·133] | [·0135,·2098] | [·1038,·3389] |  |
|  | Number of participants | 37 | 1 | 2 | 11 | 51 |
|  |  |  |  |  |  |  |
| Bulgaria | Proportion | 0·12 | 0·12 | 0·32 | 0·44 | 1 |
|  | (95%CI) | [·0143,·5616] | [·0143,·5616] | [·0472,·8172] | [·0975,·8511] |  |
|  | Number of participants | 1 | 1 | 1 | 2 | 5 |
|  |  |  |  |  |  |  |
| Croatia | Proportion | 0·5548 | 0·1095 | 0·1555 | 0·1802 | 1 |
|  | (95%CI) | [·3605,·7336] | [·0314,·3184] | [·0575,·357] | [·0773,·3658] |  |
|  | Number of participants | 16 | 3 | 4 | 6 | 29 |
|  |  |  |  |  |  |  |
| Cyprus | Proportion | 0·8058 | 0 | 0·068 | 0·1262 | 1 |
|  | (95%CI) | [·4981,·9455] | | [·0088,·3757] | [·028,·4205] |  |
|  | Number of participants | 9 | 0 | 1 | 2 | 12 |
|  |  |  |  |  |  |  |
| Czech Rep· | Proportion | 1 | 0 | 0 | 0 | 1 |
|  | (95%CI) |  |  |  |  |  |
|  | Number of participants | 1 | 0 | 0 | 0 | 1 |
|  |  |  |  |  |  |  |
| Denmark | Proportion | 0 | 0 | 0·2857 | 0·7143 | 1 |
|  | (95%CI) |  |  | [·0614,·7098] | [·2902,·9386] |  |
|  | Number of participants | 0 | 0 | 2 | 3 | 5 |
|  |  |  |  |  |  |  |
| Finland | Proportion | 0·5232 | 0 | 0·3629 | 0·1139 | 1 |
|  | (95%CI) | [·3242,·7151] | | [·1923,·5766] | [·0362,·3054] |  |
|  | Number of participants | 13 | 0 | 8 | 3 | 24 |
|  |  |  |  |  |  |  |
| France | Proportion | 0·9143 | 0 | 0·0571 | 0·0286 | 1 |
|  | (95%CI) | [·7093,·979] |  | [·008,·3119] | [·006,·1258] |  |
|  | Number of participants | 28 | 0 | 1 | 2 | 31 |
|  |  |  |  |  |  |  |
| Germany | Proportion | 0·362 | 0·0184 | 0·0429 | 0·5767 | 1 |
|  | (95%CI) | [·1967,·568] | [·0025,·1222] | [·0122,·1402] | [·3788,·7527] |  |
|  | Number of participants | 19 | 1 | 3 | 28 | 51 |
|  |  |  |  |  |  |  |
| Greece | Proportion | 0·6507 | 0·0351 | 0·177 | 0·1372 | 1 |
|  | (95%CI) | [·4962,·779] | [·0068,·1624] | [·0858,·3302] | [·063,·2731] |  |
|  | Number of participants | 46 | 2 | 8 | 8 | 64 |
|  |  |  |  |  |  |  |
| Hungary | Proportion | 0·2881 | 0·1186 | 0·0508 | 0·5424 | 1 |
|  | (95%CI) | [·1051,·5826] | [·0171,·51] | [·0069,·2931] | [·2776,·7852] |  |
|  | Number of participants | 4 | 1 | 1 | 8 | 14 |
|  |  |  |  |  |  |  |
| Ireland | Proportion | 0·4667 | 0 | 0 | 0·5333 | 1 |
|  | (95%CI) | [·0695,·9111] | |  | [·0889,·9305] |  |
|  | Number of participants | 1 | 0 | 0 | 2 | 3 |
|  |  |  |  |  |  |  |
| Israel | Proportion | 1 | 0 | 0 | 0 | 1 |
|  | (95%CI) |  |  |  |  |  |
|  | Number of participants | 2 | 0 | 0 | 0 | 2 |
|  |  |  |  |  |  |  |
| Italy | Proportion | 0·6678 | 0·0302 | 0·094 | 0·2081 | 1 |
|  | (95%CI) | [·4222,·8468] | [·0068,·1234] | [·0168,·3864] | [·09,·4111] |  |
|  | Number of participants | 14 | 2 | 2 | 9 | 27 |
|  |  |  |  |  |  |  |
| Latvia | Proportion | 0·8 | 0 | 0·2 | 0 | 1 |
|  | (95%CI) | [·3213,·9713] | | [·0287,·6787] | |  |
|  | Number of participants | 8 | 0 | 1 | 0 | 9 |
|  |  |  |  |  |  |  |
| Lithuania | Proportion | 0·8958 | 0 | 0 | 0·1042 | 1 |
|  | (95%CI) | [·6302,·9775] | |  | [·0225,·3698] |  |
|  | Number of participants | 10 | 0 | 0 | 2 | 12 |
|  |  |  |  |  |  |  |
| Malta | Proportion | 0·345 | 0·0431 | 0·0377 | 0·5741 | 1 |
|  | (95%CI) | [·2121,·5075] | [·0106,·1596] | [·0053,·223] | [·4127,·7212] |  |
|  | Number of participants | 14 | 2 | 1 | 22 | 39 |
|  |  |  |  |  |  |  |
| Netherlands | Proportion | 0·9224 | 0·0111 | 0·0259 | 0·0407 | 1 |
|  | (95%CI) | [·796,·9731] | [·0015,·0755] | [·0036,·1622] | [·0089,·1671] |  |
|  | Number of participants | 46 | 1 | 1 | 2 | 50 |
|  |  |  |  |  |  |  |
| Norway | Proportion | 0·4851 | 0·0205 | 0·2687 | 0·2257 | 1 |
|  | (95%CI) | [·3493,·6231] | [·0029,·1322] | [·1619,·4113] | [·1324,·3578] |  |
|  | Number of participants | 25 | 1 | 14 | 13 | 53 |
|  |  |  |  |  |  |  |
| Poland | Proportion | 0·4118 | 0·0112 | 0·0784 | 0·4986 | 1 |
|  | (95%CI) | [·2851,·5514] | [·0026,·0464] | [·0325,·1776] | [·3631,·6343] |  |
|  | Number of participants | 39 | 2 | 9 | 34 | 84 |
|  |  |  |  |  |  |  |
| Portugal | Proportion | 0·9062 | 0 | 0·0089 | 0·0848 | 1 |
|  | (95%CI) | [·7831,·9628] | | [·0012,·0623] | [·0314,·2096] |  |
|  | Number of participants | 42 | 0 | 1 | 5 | 48 |
|  |  |  |  |  |  |  |
| Romania | Proportion | 0·4643 | 0 | 0·1071 | 0·4286 | 1 |
|  | (95%CI) | [·1593,·7985] | | [·0141,·5011] | [·1401,·7755] |  |
|  | Number of participants | 3 | 0 | 1 | 3 | 7 |
|  |  |  |  |  |  |  |
| Slovenia | Proportion | 0·8416 | 0 | 0·0497 | 0·1087 | 1 |
|  | (95%CI) | [·679,·9303] |  | [·012,·1836] | [·04,·2628] |  |
|  | Number of participants | 27 | 0 | 2 | 4 | 33 |
|  |  |  |  |  |  |  |
| Spain | Proportion | 0·635 | 0·0312 | 0·1289 | 0·2049 | 1 |
|  | (95%CI) | [·5002,·7515] | [·0067,·1331] | [·0608,·2528] | [·1166,·3348] |  |
|  | Number of participants | 49 | 2 | 7 | 12 | 70 |
|  |  |  |  |  |  |  |
| Sweden | Proportion | 0·67 | 0 | 0 | 0·33 | 1 |
|  | (95%CI) | [·2292,·9327] | |  | [·0673,·7708] |  |
|  | Number of participants | 10 | 0 | 0 | 2 | 12 |
|  |  |  |  |  |  |  |
| Switzerland | Proportion | 0·9171 | 0 | 0 | 0·0829 | 1 |
|  | (95%CI) | [·7435,·9769] | |  | [·0231,·2565] |  |
|  | Number of participants | 19 | 0 | 0 | 3 | 22 |
|  |  |  |  |  |  |  |
| Ukraine | Proportion | 0·6485 | 0·113 | 0·1234 | 0·1151 | 1 |
|  | (95%CI) | [·4794,·7871] | [·0408,·2763] | [·0506,·2712] | [·0447,·2654] |  |
|  | Number of participants | 32 | 6 | 8 | 7 | 53 |
|  |  |  |  |  |  |  |
| UK | Proportion | 0·3503 | 0·042 | 0·0153 | 0·5924 | 1 |
|  | (95%CI) | [·2435,·4746] | [·0105,·1531] | [·0021,·101] | [·468,·7059] |  |
|  | Number of participants | 26 | 2 | 1 | 47 | 76 |
|  |  |  |  |  |  |  |
| Total | Proportion | 0·588 | 0·0289 | 0·099 | 0·2841 | 1 |
|  | (95%CI) | [·5504,·6247] | [·0189,·044] | [·0786,·1239] | [·2516,·3191] |  |
|  | Number of participants | 566 | 29 | 91 | 276 | 962 |

| **Country** |  | **Lactate** | | | | |
| --- | --- | --- | --- | --- | --- | --- |
|  |  | I would not use the test | I would use both POCT and lab versions of the test | I would use only the lab version of the test | I would use only the POCT version | Total |
|  |  |  |  |  |  |  |
| Austria | Proportion | 0·8158 | 0 | 0·0346 | 0·1496 | 1 |
|  | (95%CI) | [·6739,·9047] | | [·0049,·2068] | [·0739,·2793] |  |
|  | Number of participants | 65 | 0 | 1 | 9 | 75 |
|  |  |  |  |  |  |  |
| Belgium | Proportion | 0·795 | 0·0205 | 0·1116 | 0·0729 | 1 |
|  | (95%CI) | [·5731,·9181] | [·0028,·133] | [·0231,·4006] | [·022,·2154] |  |
|  | Number of participants | 45 | 1 | 2 | 3 | 51 |
|  |  |  |  |  |  |  |
| Bulgaria | Proportion | 0·44 | 0·12 | 0·12 | 0·32 | 1 |
|  | (95%CI) | [·0975,·8511] | [·0143,·5616] | [·0143,·5616] | [·0472,·8172] |  |
|  | Number of participants | 2 | 1 | 1 | 1 | 5 |
|  |  |  |  |  |  |  |
| Croatia | Proportion | 0·8622 | 0·0106 | 0·0989 | 0·0283 | 1 |
|  | (95%CI) | [·6611,·9525] | [·0014,·0736] | [·0252,·3184] | [·0039,·1775] |  |
|  | Number of participants | 25 | 1 | 2 | 1 | 29 |
|  |  |  |  |  |  |  |
| Cyprus | Proportion | 0·8738 | 0·0583 | 0 | 0·068 | 1 |
|  | (95%CI) | [·5795,·972] | [·0074,·3376] | | [·0088,·3757] |  |
|  | Number of participants | 10 | 1 | 0 | 1 | 12 |
|  |  |  |  |  |  |  |
| Czech Rep· | Proportion | 1 | 0 | 0 | 0 | 1 |
|  | (95%CI) |  |  |  |  |  |
|  | Number of participants | 1 | 0 | 0 | 0 | 1 |
|  |  |  |  |  |  |  |
| Denmark | Proportion | 1 | 0 | 0 | 0 | 1 |
|  | (95%CI) |  |  |  |  |  |
|  | Number of participants | 5 | 0 | 0 | 0 | 5 |
|  |  |  |  |  |  |  |
| Finland | Proportion | 0·8397 | 0 | 0·1055 | 0·0549 | 1 |
|  | (95%CI) | [·6079,·9465] | | [·0268,·3356] | [·0077,·3018] |  |
|  | Number of participants | 21 | 0 | 2 | 1 | 24 |
|  |  |  |  |  |  |  |
| France | Proportion | 0·8571 | 0 | 0·0571 | 0·0857 | 1 |
|  | (95%CI) | [·6341,·9541] | | [·008,·3119] | [·021,·2907] |  |
|  | Number of participants | 27 | 0 | 1 | 3 | 31 |
|  |  |  |  |  |  |  |
| Germany | Proportion | 0·7791 | 0·0184 | 0·0184 | 0·184 | 1 |
|  | (95%CI) | [·5341,·9157] | [·0025,·1222] | [·0025,·1222] | [·0587,·4494] |  |
|  | Number of participants | 45 | 1 | 1 | 4 | 51 |
|  |  |  |  |  |  |  |
| Greece | Proportion | 0·8246 | 0·0287 | 0·0383 | 0·1085 | 1 |
|  | (95%CI) | [·6805,·9121] | [·004,·1773] | [·0114,·121] | [·0427,·249] |  |
|  | Number of participants | 55 | 1 | 3 | 5 | 64 |
|  |  |  |  |  |  |  |
| Hungary | Proportion | 0·661 | 0·1186 | 0 | 0·2203 | 1 |
|  | (95%CI) | [·3776,·8624] | [·0171,·51] |  | [·0793,·4812] |  |
|  | Number of participants | 9 | 1 | 0 | 4 | 14 |
|  |  |  |  |  |  |  |
| Ireland | Proportion | 0·4667 | 0 | 0 | 0·5333 | 1 |
|  | (95%CI) | [·0695,·9111] | |  | [·0889,·9305] |  |
|  | Number of participants | 1 | 0 | 0 | 2 | 3 |
|  |  |  |  |  |  |  |
| Israel | Proportion | 1 | 0 | 0 | 0 | 1 |
|  | (95%CI) |  |  |  |  |  |
|  | Number of participants | 2 | 0 | 0 | 0 | 2 |
|  |  |  |  |  |  |  |
| Italy | Proportion | 0·8423 | 0·0168 | 0·1141 | 0·0268 | 1 |
|  | (95%CI) | [·5948,·951] | [·0022,·1163] | [·025,·393] | [·0061,·11] |  |
|  | Number of participants | 22 | 1 | 2 | 2 | 27 |
|  |  |  |  |  |  |  |
| Latvia | Proportion | 0·4889 | 0 | 0·2 | 0·3111 | 1 |
|  | (95%CI) | [·1691,·8181] | | [·0287,·6787] | [·0781,·7066] |  |
|  | Number of participants | 6 | 0 | 1 | 2 | 9 |
|  |  |  |  |  |  |  |
| Lithuania | Proportion | 0·9479 | 0 | 0 | 0·0521 | 1 |
|  | (95%CI) | [·6864,·9934] | |  | [·0066,·3136] |  |
|  | Number of participants | 11 | 0 | 0 | 1 | 12 |
|  |  |  |  |  |  |  |
| Malta | Proportion | 0·628 | 0 | 0·0997 | 0·2722 | 1 |
|  | (95%CI) | [·4623,·7683] | | [·0322,·2693] | [·1545,·4337] |  |
|  | Number of participants | 25 | 0 | 3 | 11 | 39 |
|  |  |  |  |  |  |  |
| Netherlands | Proportion | 0·8854 | 0 | 0 | 0·1146 | 1 |
|  | (95%CI) | [·7537,·9512] | |  | [·0488,·2463] |  |
|  | Number of participants | 44 | 0 | 0 | 6 | 50 |
|  |  |  |  |  |  |  |
| Norway | Proportion | 0·8526 | 0·0205 | 0·0821 | 0·0448 | 1 |
|  | (95%CI) | [·7153,·9302] | [·0029,·1322] | [·0266,·2267] | [·0143,·1312] |  |
|  | Number of participants | 46 | 1 | 3 | 3 | 53 |
|  |  |  |  |  |  |  |
| Poland | Proportion | 0·7577 | 0·0644 | 0·0728 | 0·105 | 1 |
|  | (95%CI) | [·6278,·8529] | [·0225,·1711] | [·0257,·1893] | [·0514,·2028] |  |
|  | Number of participants | 61 | 5 | 6 | 12 | 84 |
|  |  |  |  |  |  |  |
| Portugal | Proportion | 0·9063 | 0 | 0 | 0·0937 | 1 |
|  | (95%CI) | [·7446,·9698] | |  | [·0302,·2554] |  |
|  | Number of participants | 45 | 0 | 0 | 3 | 48 |
|  |  |  |  |  |  |  |
| Romania | Proportion | 1 | 0 | 0 | 0 | 1 |
|  | (95%CI) |  |  |  |  |  |
|  | Number of participants | 7 | 0 | 0 | 0 | 7 |
|  |  |  |  |  |  |  |
| Slovenia | Proportion | 1 | 0 | 0 | 0 | 1 |
|  | (95%CI) |  |  |  |  |  |
|  | Number of participants | 33 | 0 | 0 | 0 | 33 |
|  |  |  |  |  |  |  |
| Spain | Proportion | 0·7368 | 0·0543 | 0·0719 | 0·137 | 1 |
|  | (95%CI) | [·6001,·8393] | [·0163,·1661] | [·0259,·1843] | [·0655,·2647] |  |
|  | Number of participants | 56 | 3 | 4 | 7 | 70 |
|  |  |  |  |  |  |  |
| Sweden | Proportion | 0·71 | 0 | 0 | 0·29 | 1 |
|  | (95%CI) | [·2289,·9528] | |  | [·0472,·7711] |  |
|  | Number of participants | 11 | 0 | 0 | 1 | 12 |
|  |  |  |  |  |  |  |
| Switzerland | Proportion | 0·9366 | 0 | 0 | 0·0634 | 1 |
|  | (95%CI) | [·7531,·9862] | |  | [·0138,·2469] |  |
|  | Number of participants | 20 | 0 | 0 | 2 | 22 |
|  |  |  |  |  |  |  |
| Ukraine | Proportion | 0·659 | 0·0167 | 0·0418 | 0·2824 | 1 |
|  | (95%CI) | [·4908,·7948] | [·0041,·0663] | [·0144,·1154] | [·1558,·4564] |  |
|  | Number of participants | 34 | 2 | 4 | 13 | 53 |
|  |  |  |  |  |  |  |
| UK | Proportion | 0·549 | 0·0191 | 0·0153 | 0·4166 | 1 |
|  | (95%CI) | [·4273,·6652] | [·0027,·1235] | [·0038,·0598] | [·3034,·5393] |  |
|  | Number of participants | 42 | 1 | 2 | 31 | 76 |
|  |  |  |  |  |  |  |
| Total | Proportion | 0·784 | 0·0209 | 0·0489 | 0·1463 | 1 |
|  | (95%CI) | [·7502,·8143] | [·0126,·0344] | [·0337,·0704] | [·121,·1757] |  |
|  | Number of participants | 776 | 20 | 38 | 128 | 962 |
